# Safety, tolerability, and *Plasmodium falciparum* transmission-reducing activity of monoclonal antibody TB31F: a single-centre, open-label, first-in-human, dose-escalation, phase 1 trial in healthy malaria-naive adults

**DOI:** 10.1016/S1473-3099(22)00428-5

**Published:** 2022-11

**Authors:** Saskia C van der Boor, Merel J Smit, Stijn W van Beek, Jordache Ramjith, Karina Teelen, Marga van de Vegte-Bolmer, Geert-Jan van Gemert, Peter Pickkers, Yimin Wu, Emily Locke, Shwu-Maan Lee, John Aponte, C Richter King, Ashley J Birkett, Kazutoyo Miura, Morolayo A Ayorinde, Robert W Sauerwein, Rob ter Heine, Christian F Ockenhouse, Teun Bousema, Matthijs M Jore, Matthew B B McCall

**Affiliations:** a Department of Medical Microbiology, Radboud University Medical Center, Nijmegen, Netherlands; a Department of Pharmacy, Radboud University Medical Center, Nijmegen, Netherlands; c Department for Health Evidence, Biostatistics Section, Radboud University Medical Center, Nijmegen, Netherlands; d Department of Intensive Care, Radboud University Medical Center, Nijmegen, Netherlands; e Radboud Institute for Molecular Life Sciences, Radboud University Medical Center, Nijmegen, Netherlands; f Radboud Institute for Health Sciences, Radboud University Medical Center, Nijmegen, Netherlands; g PATH's Malaria Vaccine Initiative, PATH, Seattle, WA, USA; h Laboratory of Malaria and Vector Research, National Institute of Allergy and Infectious Diseases, National Institutes of Health, Rockville, MD, USA; i Human Immunology Laboratory, Imperial College London, London, UK; j TropIQ Health Sciences, Nijmegen, Netherlands; k Institut für Tropenmedizin, Universitätsklinikum Tübingen, Tübingen, Germany; l Centre de Recherches Médicales de Lambaréné, Lambaréné, Gabon

## Abstract

**Background:**

Malaria elimination requires interruption of the highly efficient transmission of *Plasmodium* parasites by mosquitoes. TB31F is a humanised monoclonal antibody that binds the gamete surface protein Pfs48/45 and inhibits fertilisation, thereby preventing further parasite development in the mosquito midgut and onward transmission. We aimed to evaluate the safety and efficacy of TB31F in malaria-naive participants.

**Methods:**

In this open-label, first-in-human, dose-escalation, phase 1 clinical trial, healthy, malaria-naive, adult participants were administered a single intravenous dose of 0·1, 1, 3, or 10 mg/kg TB31F or a subcutaneous dose of 100 mg TB31F, and monitored until day 84 after administration at a single centre in the Netherlands. The primary outcome was the frequency and magnitude of adverse events. Additionally, TB31F serum concentrations were measured by ELISA. Transmission-reducing activity (TRA) of participant sera was assessed by standard membrane feeding assays with *Anopheles stephensi* mosquitoes and cultured *Plasmodium falciparum* gametocytes. The trial is registered with Clinicaltrials.gov, NCT04238689.

**Findings:**

Between Feb 17 and Dec 10, 2020, 25 participants were enrolled and sequentially assigned to each dose (n=5 per group). No serious or severe adverse events occurred. In total, 33 grade 1 and six grade 2 related adverse events occurred in 20 (80%) of 25 participants across all groups. Serum of all participants administered 1 mg/kg, 3 mg/kg, or 10 mg/kg TB31F intravenously had more than 80% TRA for 28 days or more, 56 days or more, and 84 days or more, respectively. The TB31F serum concentration reaching 80% TRA was 2·1 μg/mL (95% CI 1·9–2·3). Extrapolating the duration of TRA from antibody kinetics suggests more than 80% TRA is maintained for 160 days (95% CI 136–193) following a single intravenous 10 mg/kg dose.

**Interpretation:**

TB31F is a well tolerated and highly potent monoclonal antibody capable of completely blocking transmission of *P falciparum* parasites from humans to mosquitoes. In areas of seasonal transmission, a single dose might cover an entire malaria season.

**Funding:**

PATH's Malaria Vaccine Initiative.

## Introduction

Malaria is a global health priority with over 200 million yearly cases worldwide, including more than half a million deaths resulting primarily from *Plasmodium falciparum* infections.^1^ Malaria infections and deaths have been rising over the past five years.^1^ The emergence and spread of artemisinin resistance in Africa warns that gains in reducing malaria burden, which were attributed in part to access to efficacious artemisinin-based treatment, are under threat.^2^, ^3^

In 2021, landmark progress was achieved in the field of malaria vaccines. WHO issued a recommendation for widespread use of RTS,S/AS01, the world's first approved malaria vaccine, among children living in regions with moderate to high *P falciparum* malaria transmission. Despite its important value in reducing malaria morbidity and mortality, deployment of RTS,S/AS01 will be insufficient to prevent all episodes of malaria. Moreover, this vaccine does not directly prevent onward transmission of parasites to mosquitoes and remains susceptible to selection and spread of escape mutants. The high efficacy of monoclonal antibody CIS43LS in preventing infections in a human challenge model underscores the clinical potential of monoclonal antibodies as a complementary tool to combat malaria, as shown for other infectious diseases.^4^, ^5^, ^6^, ^7^

Malaria transmission depends on the uptake of male and female gametocytes by blood feeding *Anopheles* mosquitoes in whose midgut gametocytes activate into gametes, fertilise, and ultimately render the mosquito infectious to the next human host. The long duration of (asymptomatic) *P falciparum* infections in humans, the abundance of mosquitoes that bite multiple hosts, and efficient transmission of parasites from humans to mosquitoes and vice versa, all contribute to reproduction rates (R_0_) for malaria that exceed 100 in many African settings.^8^

**Research in context**

**Evidence before this study**

Malaria is a global health priority with over 200 million cases and over 500 000 deaths that result primarily from *Plasmodium falciparum* infections. In addition to strategies focusing on preventing and treating individual cases of malaria, interrupting the highly efficient transmission of malaria is key to reducing the global burden of this disease. We searched PubMed on March 8, 2022, for clinical trials testing transmission blocking vaccines and monoclonal antibodies against *P falciparum* malaria, using the search terms: (falciparum OR malaria) AND ((transmission blocking) OR (transmission-blocking) OR (transmission reducing) OR (transmission-reducing)) AND (vaccine OR monoclonal). No language or date restrictions were applied. No records were present of clinical trials involving transmission-blocking monoclonal antibodies; although, one study reports the clinical testing of a monoclonal antibody that targets the pre-erythrocytic stage of the parasite and hence can prevent infection. Among transmission-blocking monoclonal antibodies evaluated to date in preclinical studies, the humanised antibody TB31F has shown the most potent functional activity.

**Added value of this study**

This is the first clinical trial assessing the safety and efficacy of a *P falciparum* transmission blocking monoclonal antibody (TB31F). We report TB31F administration to malaria-naive healthy participants to be safe and well tolerated at all doses tested. TB31F in participants' serum showed potent activity, reaching the benchmark of at least 80% reduction in transmission of parasites to mosquitoes in the standard membrane feeding assay at serum concentrations as low as 2·1 μg/mL. Moreover, such activity was estimated to last for up to 5 months after a single administration.

**Implications of all the available evidence**

The preliminary safety and highly promising efficacy profile of TB31F, in particular its ability to potently block transmission of *P falciparum* parasites to mosquitoes, underscore its clinical potential. Current malaria control measures, even when complemented by the widespread deployment of the pre-erythrocytic malaria vaccine RTS,S/AS01, as recommended by WHO in October, 2021, will be insufficient to prevent all episodes of malaria and subsequent onward transmission of parasites. TB31F represents a promising complementary tool to be deployed in several use-scenarios, including in areas of seasonal malaria transmission, where a single administration might have transmission-blocking activity for the duration of an entire season.

Malaria transmission-blocking vaccines aim to induce antibodies that target the surface of sexual stage parasites and are coingested with gametocytes during the mosquito bloodmeal, consequently preventing parasite development in the mosquito midgut and hence onward transmission.^9^ The leading transmission-blocking vaccine targets are Pfs48/45 and Pfs230, two surface proteins expressed on *P falciparum* gametocytes and early gametes, that are being evaluated in phase 1 (NCT04862416) and phase 2 (NCT03917654) clinical trials, respectively. The effect of these vaccines will depend on their capacity to induce and maintain effective antibody titres against specific key epitopes,^10^ whereby the titre required for functional activity is target dependent and antibody dependent. The most potent transmission-blocking antibody described to date is the rat monoclonal antibody 85RF45.1, which was derived from rats immunised with *P falciparum* NF54 gametocytes.^11^ Antibody 85RF45.1 targets a highly conserved epitope on Pfs48/45 and blocks onward transmission of genetically diverse parasites to other humans.^9^, ^11^, ^12^, ^13^ This monoclonal antibody has been humanised as TB31F, which maintains the original binding characteristics and potency.^12^ A safe and highly efficacious transmission-blocking monoclonal antibody for administration in humans, when used in combination with other malaria control measures, would be a valuable tool for malaria elimination. Here, we report the safety, tolerability, pharmacokinetics, and functional transmission-reducing activity (TRA) of the humanised monoclonal antibody TB31F.

## Methods

### Study design and participants

This single-centre, open-label, first-in-human, dose-escalation, phase 1 clinical study evaluating the safety and efficacy of humanised monoclonal antibody TB31F (appendix p 2) was done at the Radboud University Medical Center (Radboudumc) in Nijmegen, the Netherlands, between Jan 23, 2020, and March 4, 2021. The study received approval from the Arnhem–Nijmegen Committee on Research Involving Human Subjects (NL69779.091.19). The study was done in accordance with the latest Fortaleza revision of the Declaration of Helsinki (2013), the Netherlands' Medical Research Involving Human Subjects Act, ICH Good Clinical Practice standards, and local regulatory requirements. The study population was composed of healthy adults aged 18–35 years who are malaria-naive. Upon informed written consent, participants were screened for eligibility as described in the protocol (appendix pp 32–117).

### Study procedures

**Figure 1:**
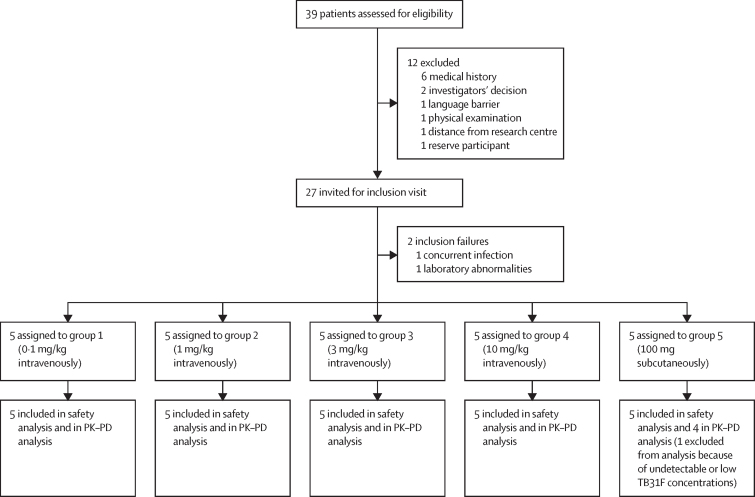
Study profile PK–PD analysis=pharmacokinetic and pharmacodynamic analysis.

25 eligible participants were included and upon enrolment sequentially allocated from group 1 to group 5 (n=5 per group; figure 1). Participants in groups 1–4 received 0·1, 1, 3, and 10 mg/kg humanised monoclonal antibody TB31F by intravenous infusion, respectively. Group 5 participants (n=5) received 100 mg TB31F subcutaneously. TB31F administration took place in the medium care unit under direct clinical supervision. Before administration, all participants received premedication consisting of 1000 mg paracetamol orally and 2 mg clemastine intravenously as a safety precaution. TB31F was administered via slow intravenous drip to participants of groups 1–4, at increasing infusion rates per dose group (appendix pp 10–11) as an additional precautionary measure against infusion reactions. TB31F was administered subcutaneously (abdomen) to participants of group 5, divided equally in volumes of 1 mL between two injection sites.

On the day of administration, collection of vital signs, safety data, and serum for pharmacokinetic and pharmacodynamic measurements was done before infusion or administration, upon end of infusion or administration, and 1, 3, and 6 h after end of infusion or administration. All participants were observed for 6 h after end of infusion or administration. Escalation to the next higher dose group was dependent on the absence of safety signals and on positive approval by an independent safety monitoring committee.

Follow-up visits were done on days 1, 2, 7, 14, 21, 28, 56, and 84 after TB31F administration. Group 5 participants were additionally seen on days 4 and 10 after administration to detect peak TB31F concentrations. During follow-up visits, clinical safety data and blood samples for safety, pharmacokinetic, and pharmacodynamic analyses were collected. Due to COVID-19-related restrictions in force at the time, the routine follow-up visit scheduled 56 days after administration could not be done for group 1 participants.

### Outcomes

Primary outcomes included the number and severity of solicited local and systemic adverse events, unsolicited adverse events, serious adverse events, and clinically significant laboratory abnormalities. Secondary outcomes included serum TB31F monoclonal antibody concentration over time to determine pharmacokinetic parameters, and functional TRA as assessed by standard membrane feeding assay (SMFA).

Adverse events were recorded and graded by the attending clinical investigator as mild (grade 1; easily tolerated), moderate (grade 2; interferes with daily activity) or severe (grade 3; prevents normal activity; appendix pp 12–13, and in the protocol). All participants received a memory aid booklet to register symptoms throughout the study and an oral thermometer to register their daily body temperature for 6 consecutive days after TB31F administration. Solicited local adverse events were defined as pain, redness, and swelling at the injection site up to 7 days after TB31F administration. Solicited systemic adverse events were defined as fever, headache, myalgia, fatigue, chills, and rash up to 28 days after TB31F administration. Unsolicited and serious adverse events were recorded through to the end of the study (day 84). Clinical laboratory results assessed as adverse events were scored for severity with the adapted Food and Drug Administration Toxicity Tables. Antidrug antibody levels in participants were assessed by sandwich ELISA at the Human Immunology Laboratory in London, UK, as an exploratory outcome (appendix p 3).

Serum concentrations of TB31F were measured by ELISA against the recombinant protein R0·6C, which contains the 6C fragment of the Pfs48/45 antigen to which TB31F binds.^14^ Antibody concentrations were quantified with ADAMSEL software (version 1.1) with a TB31F standard curve and three duplicate serum dilutions. The limit of detection was determined by the serum dilutions tested, and therefore, differed per group; the detection limit was 0·039 μg/mL for group 1 (0·1 mg/kg intravenously), 0·39 μg/mL for group 2 (1 mg/kg intravenously), 1·17 μg/mL for group 3 (3 mg/kg intravenously), 3·91 μg/mL for group 4 (10 mg/kg intravenously), and 0·39 μg/mL for group 5 (100 mg subcutaneously) samples.

For the intravenous and subcutaneous groups, the following pharmacokinetic parameters were determined by non-compartmental analysis for each individual curve: the maximum concentration (C_max_), the area under the concentration-time curve from 0 to 12 weeks, and from time zero to infinity with the trapezoidal rule, and the elimination half-life. The apparent clearance and volume of distribution were additionally determined for individuals receiving intravenous TB31F administration. The bioavailability and the time of C_max_ (T_max_) were additionally determined for individuals in the subcutaneous administration group. Projected TB31F concentrations beyond day 84 were estimated for each participant with concentrations from day 7 to day 84 after administration assuming first-order kinetics.

TRA was determined at predefined intervals by SMFA with participants' sera.^15^ In short, 90 μL of participant's serum was mixed with 150 μL packed red blood cells and cultured *P falciparum* NF54 gametocytes, and 30 μL naive human serum containing active complement, before feeding to *Anopheles stephensi* (Sind-Kasur Nijmegen strain) mosquitoes. Time series of individual participants were grouped in the same SMFA experiment. Additional SMFA with sera from participants receiving 10 mg/kg TB31F was done as a post-hoc analysis to determine TRA against the genetically distant Asian *P falciparum* isolate NF135.^16^ TRA was expressed as the reduction of oocyst count in mosquitoes fed on gametocytes in the presence of participants' serum (or purified IgG) compared with mosquitoes fed on gametocytes in the presence of pooled naive serum (or IgG).^15^, ^17^ Given the lower precision of low TRA estimates and the historical threshold value of more than 80% TRA to support clinical development of transmission-blocking vaccines, TRA of more than 80% was predefined as the efficacy threshold of interest.^18^, ^19^

### Statistical analysis

Study sample size was chosen pragmatically on the basis of the assessment of safety and in line with similar first-in-human dose-escalation trials. The study's original sample size of 20 (four intravenous groups of five participants each) was determined to allow a power of 90% to observe at least one event if the true rate of such an event is 10·9% or more; and a 90% chance of observing no events if the true rate is 0·5% or less. The fifth (subcutaneous) group was added later and pragmatically included the same number of participants as the other groups. Safety data are shown as frequencies and percentages are tabulated. TB31F pharmacokinetic parameters were assessed by standard non-compartmental methods, with multiple observations per group and per study participant. TB31F serum concentration (ie, concentration in the total liquid volume of the bloodmeal) at which 80% TRA is expected (IC_80_) was estimated by linear regression, regressing the square root of serum concentration on the log-mean oocyst ratio^17^—ie,

$$IC80={\left(\frac{\left(\log(\frac{100}{100-80})-{\hat{\beta }}_{0}\right)}{{\hat{\beta }}_{1}}\right)}^{2}$$

where ${\hat{\beta }}_{0}$ and ${\hat{\beta }}_{1}$ are the estimated regression coefficients for the intercept and slope, respectively. The delta method was used to calculate 95% CIs for the IC_80_.

Safety data analysis was done in IBM SPSS Statistics (version 25.0.0.1) and Prism 9 (version 9.2.0). SMFA data analysis was done in R (version 4.1.1) and R studio (version 1.4.1717). Pharmacokinetic parameters were determined with non-compartmental analysis in Phoenix WinNonlin (version 8.3). The trial is registered with ClinicalTrials.gov, NCT04238689.

### Role of the funding source

This study was funded by PATH's Malaria Vaccine Initiative, Washington, DC, USA. The funder was involved in study design, data collection, analysis, and interpretations, and contributed to the writing of the report.

## Results

From Jan 23 to Dec 8, 2020, 39 participants were screened for eligibility. In total, 25 participants were enrolled between Feb 17 and Dec 10, 2020 (figure 1). Baseline characteristics were similar between the five study groups (table 1). Overall, 13 (52%) of 25 participants were women. The mean age was 23·5 years (range 19·0–34·0), the mean bodyweight was 74·2 kg (range 53·0–93·0), and the mean body-mass index was 24·1 kg/m^2^ (range 19·5–29·5). All participants remained in follow-up until the end of the study and were included in the safety analysis.

**Table 1:** Baseline characteristics

		**Group 1 (n=5)**	**Group 2 (n=5)**	**Group 3 (n=5)**	**Group 4 (n=5)**	**Group 5 (n=5)**	**Total (n=25)**
Sex							
	Women	3 (60%)	3 (60%)	2 (40%)	4 (80%)	1 (20%)	13 (52%)
	Men	2 (40%)	2 (40%)	3 (60%)	1 (20%)	4 (80%)	12 (48%)
Age, years		22·0 (21·0–24·0)	24·8 (22·0–26·0)	26·8 (24·0–34·0)	21·6 (19·0–25·0)	22·2 (19·0–25·0)	23·5 (19·0–34·0)
Weight, kg		73·6 (60·4–84·8)	75·5 (68·8–91·2)	72·0 (53·0–86·0)	71·5 (63·2–91·8)	78·4 (63.2–93·0)	74·2 (53·0–93·0)
Body-mass index, kg/m^2^		24·3 (19·5–29·5)	25·0 (22·0–27·8)	23·4 (21·6–26·4)	23·9 (21·7–29·0)	23·9 (21·7–25·0)	24·1 (19·5–29·5)

Data are n (%) or mean (range).

Both intravenous and subcutaneous administrations of TB31F were well tolerated. No serious or grade 3 (severe) adverse events occurred. Furthermore, no infusion reactions occurred and no dose adjustments or temporary interruptions of TB31F administration were necessary. Overall, 20 (80%) of 25 participants had at least one adverse event that was possibly, probably, or definitely related to TB31F administration. Most solicited local adverse events took place in the subcutaneous group in which four (80%) of five participants had grade 1 (mild) pain of short duration (≤2 min) during subcutaneous administration (table 2, appendix p 13). 13 (52%) of 25 participants had fatigue, which was the most common solicited systemic adverse event. Most of these episodes occurred on the day of TB31F administration. Additionally, one otherwise asymptomatic participant reported a grade 1 fever (38·1°C) on the day of TB31F administration, which resolved the same day and was assessed as possibly related to study product administration. Our trial was not powered to allow meaningful comparisons of the occurrence of individual adverse events between groups. Unsolicited adverse events that occurred during the study are summarised in the appendix (p 14) All adverse events are listed in the appendix (pp 15–19). No severe laboratory abnormalities occurred; all laboratory abnormalities are listed in the appendix (pp 20–30). Antidrug antibody responses were not detected in any participant at any timepoint (data not shown).

**Table 2:** Solicited adverse events

	**Group 1 (n=5)**		**Group 2 (n=5)**		**Group 3 (n=5)**		**Group 4 (n=5)**		**Group 5 (n=5)**		**All (n=25)**	
	Participants	Events	Participants	Events	Participants	Events	Participants	Events	Participants	Events	Participants	Events
**Pain**												
Grade 1	0	0	0	0	1 (20%)	1	0	0	4 (80%)	4	5 (20%)	5
Grade 2	0	0	0	0	0	0	0	0	0	0	0	0
Grade 3	0	0	0	0	0	0	0	0	0	0	0	0
Any	0	0	0	0	1 (20%)	1	0	0	4 (80%)	4	5 (20%)	5
**Redness**												
Grade 1	0	0	1 (20%)	1	0	0	0	0	0	0	1 (4%)	1
Grade 2	0	0	0	0	0	0	0	0	0	0	0	0
Grade 3	0	0	0	0	0	0	0	0	0	0	0	0
Any	0	0	1 (20%)	1	0	0	0	0	0	0	1 (4%)	1
**Fever**												
Grade 1	0	0	0	0	0	0	1 (20%)	1	0	0	1 (4%)	1
Grade 2	0	0	0	0	0	0	0	0	0	0	0	0
Grade 3	0	0	0	0	0	0	0	0	0	0	0	0
Any	0	0	0	0	0	0	1 (20%)	1	0	0	1 (4%)	1
**Headache**												
Grade 1	1 (20%)	1	1 (20%)	1	0	0	2 (40%)	6	0	0	4 (16%)	8
Grade 2	0	0	0	0	0	0	1 (20%)	1	0	0	1 (4%)	1
Grade 3	0	0	0	0	0	0	0	0	0	0	0	0
Any	1 (20%)	1	1 (20%)	1	0	0	3 (60%)	7	0	0	5 (20%)	9
**Myalgia**												
Grade 1	1 (20%)	1	0	0	0	0	0	0	0	0	1 (4%)	1
Grade 2	0	0	0	0	0	0	0	0	0	0	0	0
Grade 3	0	0	0	0	0	0	0	0	0	0	0	0
Any	1 (20%)	1	0	0	0	0	0	0	0	0	1 (4%)	1
**Fatigue**												
Grade 1	2 (40%)	4	2 (40%)	2	1 (20%)	1	2 (40%)	2	2 (40%)	2	9 (36%)	11
Grade 2	3 (60%)	3	0	0	0	0	1 (20%)	1	0	0	4 (16%)	4
Grade 3	0	0	0	0	0	0	0	0	0	0	0	0
Any	5 (100%)	7	2 (40%)	2	1 (20%)	1	3 (60%)	3	2 (40%)	2	13 (52%)	15

Possibly, probably, and definitely related adverse events are reported per group. Local adverse events (pain, redness, and local swelling) are shown from administration to day 7. No local swelling was reported in any of the participants. Solicited systemic adverse events (fever, headache, myalgia, fatigue, chills, or rash) are shown from administration to day 28. No chills or rash were reported in any of the participants.

**Figure 2:**
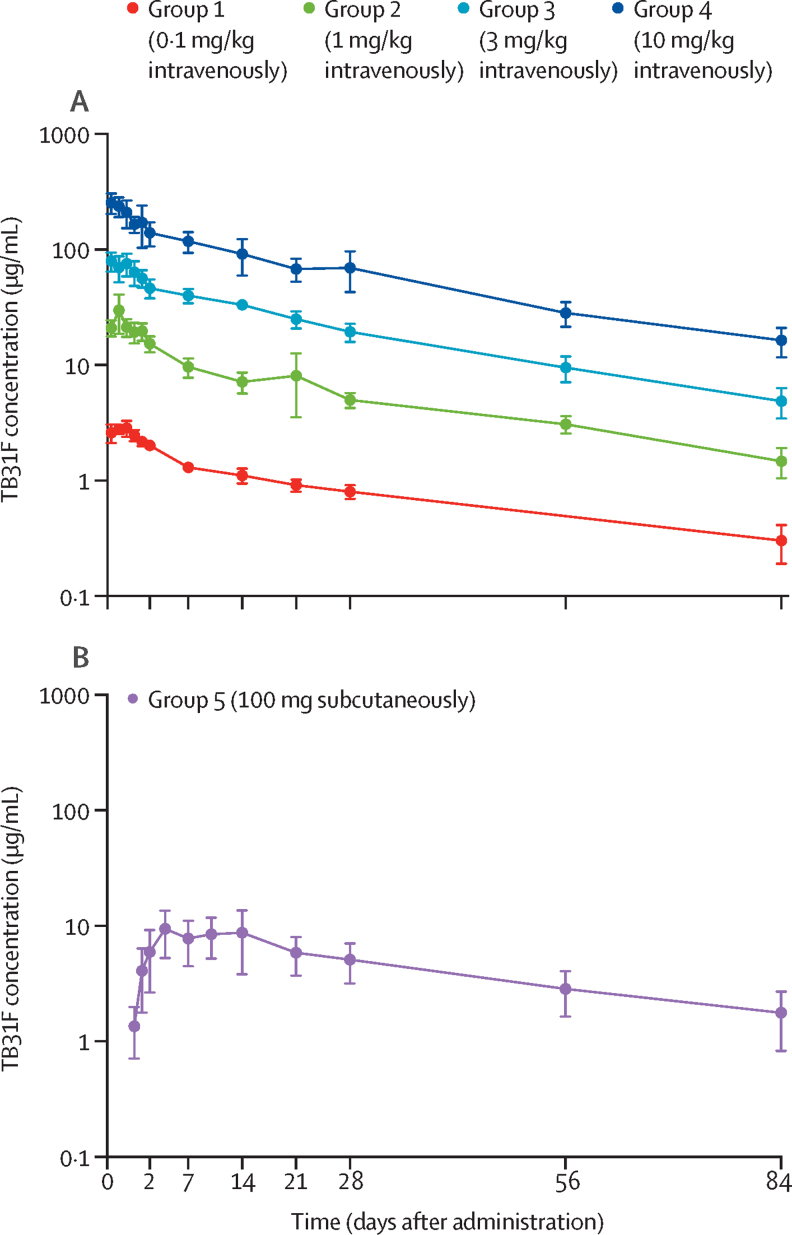
TB31F concentrations per dose group over time (A) Groups 1–4 (intravenous infusion, n=5 per group). (B) Group 5 (subcutaneous administration, n=4). Datapoints and error bars represent group mean and standard deviation, respectively. For group 1, serum TB31F concentrations were unavailable for one participant at day 28 and all five participants at day 56. One participant in group 5 had no detectable or very low concentrations of TB31F at any timepoint (appendix p 6).

TB31F displayed dose-dependent peak concentrations and a dose-independent terminal half-life (figure 2, appendix p 4). Dose-proportional pharmacokinetics were observed (appendix p 5). Pharmacokinetic parameters, as determined by non-compartmental analysis, are summarised in the appendix (p 31). TB31F administered intravenously at doses of 0·1, 1, 3, and 10 mg/kg resulted in dose-proportional maximum concentrations (C_max_, reported as geometric mean and 95% CI) of 2·94 μg/mL (95% CI 2·34–3·69), 28·3 μg/mL (14·4–55·5), 80·8 μg/mL (55·2–118), and 255 μg/mL (177–367), respectively, at end of infusion or administration (=T_max_). Subcutaneous administration of 100 mg TB31F resulted in a maximum concentration of 9·28 μg/mL (3·49–24·7) at day 4·3 (1·15–16·0), with a bioavailability of 52% (0·17–1·61), all reported as geometric mean and 95% CI. The overall terminal half-life was estimated to be 32·2 days.

TB31F remained detectable in serum until end of study at day 84 in all study groups, except for one participant in the subcutaneous group. In this participant, in contrast to other group 5 participants, no or very low concentrations of TB31F were detected in serum at any timepoint. Therefore, the data from this participant were only included in the safety analyses (antibody concentrations, where measurable, and TRA are provided for reference in the appendix p 6).

**Figure 3:**
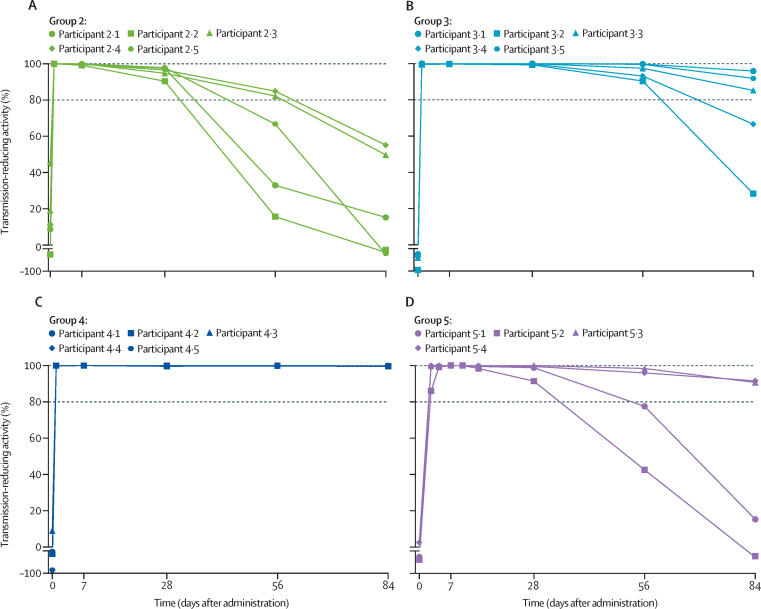
TRA of TB31F per dose group over time (A) Group 2 (1 mg/kg intravenously). (B) Group 3 (3 mg/kg intravenously). (C) Group 4 (10 mg/kg intravenously). (D) Group 5 (100 mg subcutaneously). TRA was calculated by comparing the reduction of oocyst counts of participant sera compared with pooled naive serum in standard membrane feeding assay. Each line represents a participant. The horizontal dashed lines represent the 100% and 80% TRA threshold. No participants in the lowest dose group (group 1, 0·1 mg/kg) showed TRA >80% at any time point (data not shown). One participant in group 5 had no detectable or very low concentrations of TB31F, and TRA data are shown in the appendix (p 6). TRA=transmission-reducing activity.

The functional activity of TB31F in participants' serum samples in reducing transmission to mosquitoes was expressed as TRA (figure 3). A single intravenous dose of 1 mg/kg, 3 mg/kg, or 10 mg/kg was sufficient to reach more than 80% TRA against the west African *P falciparum* strain NF54 for 28 days or more, 56 days or more, and 84 days or more, respectively, in all participants. A single intravenous dose of 10 mg/kg was also sufficient to maintain more than 80% TRA against the genetically distant Asian *P falciparum* isolate NF135 for 84 days or more (appendix p 7). Following a subcutaneous dose of 100 mg, more than 80% TRA was maintained for 28 days or more in four of five participants. The potency of administered TB31F was maintained over time (appendix p 8).

**Figure 4:**
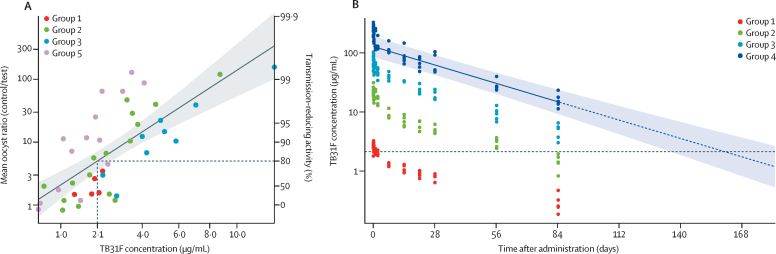
Estimation of IC_80_ and predicted time of more than 80% TRA (A) TB31F concentration in serum at which 80% TRA (horizontal dashed line) is expected (IC_80_) was calculated by linear regression to be 2·1 μg/mL (vertical dashed line). Datapoints represent measured serum TB31F concentrations and respective TRA values for individual participants in groups 1 to 5, including only those timepoints where TRA was less than 99·5%. Shaded areas represent the pointwise 95% CI for the linear association. Given that all group 4 volunteers had TRA values of more than 99·5%, no points were included in this analysis. (B) The observed TB31F concentrations of individuals (circles) were used to extrapolate TB31F concentrations over time. For each individual in group 4 (10 mg/kg), the observed concentrations in samples from day 7 to day 84 were used to extrapolate concentrations up to month 6 via linear regression. The solid line represents the geometric mean of the observed data and the dashed line represents the extrapolated linear regressions. The coloured band represents the 95% CI for the extrapolated data. The horizontal dashed black line represents the IC_80_ of 2·1 μg/mL, which is crossed on day 160 (95% CI 136–193). The confidence intervals were calculated with the geometric standard deviation assuming lognormality. TRA=transmission-reducing activity.

Combining TB31F concentrations and the corresponding TRA measured in individual participants' sera at various timepoints, the concentration of TB31F reaching 80% TRA (IC_80_) was determined to be 2·1 μg/mL (95% CI 1·9–2·3; figure 4A). Sera from participants that received 10 mg/kg TB31F (group 4) showed more than 80% TRA at the end of the follow-up (day 84). Therefore, we projected TB31F concentrations over time and extrapolated that a single intravenous dose of 10 mg/kg TB31F maintains more than 80% TRA for 160 days (95% CI 136–193; figure 4B).

## Discussion

Here, we did the first clinical evaluation of a *P falciparum* transmission-blocking monoclonal antibody. TB31F administration was well tolerated and reached a high TRA. All participants who received 1 mg/kg or more of TB31F intravenously displayed TRA above the 80% benchmark. At a single intravenous dose of 10 mg/kg, this functional activity was maintained throughout the 12 weeks of follow-up and was predicted to persist for 5 months. Interrupting the highly efficient transmission of *Plasmodium* parasites has the potential to reduce the global burden of malaria, and a safe and potent transmission-blocking monoclonal antibody could provide the means to achieve this.

Although this study was not powered to detect less frequent adverse events, the excellent safety profile of TB31F observed here is consistent with that of other humanised antibodies against infectious diseases.^5^, ^6^, ^7^ The precautionary administration of premedication was specifically intended to dampen potential allergic and infusion reactions in this first-in-human trial; the short half-lives of paracetamol and clemastine relative to that of TB31F are unlikely to have masked clinically relevant allergic reactions to TB31F. Notably, the most frequently observed adverse event, fatigue, is most likely a side-effect of the histamine antagonist clemastine. Larger trials are required to confirm this safety profile and to rule out less frequent side-effects and might also assess administration in absence of premedication. This modestly sized trial did allow detailed assessments of functional activity. By reaching TRA of 80% or more at a concentration of 2·1 μg/mL, TB31F is the most potent antimalarial monoclonal antibody described to date, confirming IC_80_ values of preclinical studies.^12^, ^20^

A few limitations of our study warrant consideration. First, one participant in the subcutaneous group appeared to exhibit no or very low concentrations of TB31F in serum and a corresponding absence of TRA. A clear explanation was not identified, but human error in study product preparation or administration cannot be fully ruled out. More generally, bioavailability after subcutaneous monoclonal antibody administration might be affected by biophysical properties of the monoclonal antibody interacting with characteristics of the subcutaneous tissue at the chosen injection site.^21^ Future clinical trials should continue to evaluate whether this is a tangible issue for subcutaneous administration of TB31F.

Second, our findings in healthy malaria-naive adult participants require confirmation in populations that might be a future target population for TB31F administration—eg, children in malaria-endemic settings who constitute the largest source of malaria transmission.^22^ Pharmacokinetics of monoclonal antibodies in children of different age groups might vary from those in adults and from each other.^23^ Additionally, protein malnutrition is more common in many resource-poor malaria-endemic regions and might affect monoclonal antibody pharmacokinetics, as observed in their use in patients with cancer.^24^, ^25^

TB31F targets a highly conserved epitope on Pfs48/45 that contains only three very rare polymorphisms.^12^ 85RF45.1, the precursor of TB31F, retains low nanomolar affinity for the recombinant protein containing these single nucleotide polymorphisms.^12^ We previously showed that 85RF45.1 has cross-strain functional activity in endemic settings.^20^ A post-hoc SMFA showed that sera with TB31F retained strong TRA against the genetically distant Asian isolate NF135. For these reasons, we predict that the efficacy of TB31F will not be affected by genetic variation in circulating field strains. This also makes TB31F a valuable tool to compare prevailing functional assays for transmission-blocking vaccines by determining the association between TB31F efficacy estimates in the in-vitro SMFA and ex-vivo mosquito membrane feedings and direct skin feeding assays, in naturally infected gametocyte carriers who receive different doses of TB31F. As a next step, it is important to consider half-life extension strategies,^26^, ^27^, ^28^ which are of particular interest to support dose-reduction, resulting in a more cost-effective intervention and lower administration volumes that facilitate subcutaneous administration for mass administration in malaria-endemic settings. Assuming an extension of TB31F half-life by two to four times, 80% TRA after a single dose of 10 mg/kg could be maintained for nearly 1 or 2 years, respectively (appendix p 9). Future studies might also administer TB31F to naturally infected gametocyte carriers to confirm its potency against genetically complex infections and directly examine the reductions that can be achieved in the proportion of mosquitoes that become infected.

Our findings support further development of TB31F as a promising cointervention tool to be studied in malaria-endemic populations, where it might be considered in seasonal settings, in mass administration campaigns to eliminate (drug-resistant) malaria, or in outbreak suppression. Mathematical modelling can inform future studies investigating the population-wide transmission-reducing potential of TB31F as an intervention strategy against malaria in different population groups at varying dosages. This includes standalone TB31F administration, and use in combination with interventions targeting other stages of the parasite lifecycle, including the recently reported CIS43LS monoclonal antibody for preventing *P falciparum* infections.^4^ Given the heterogeneity of transmission, such models should address the effect of TB31F administration to not only different demographic groups (eg, distinct age categories), but also in regions of different transmission intensity and seasonality, to further guide the effective deployment of TB31F.

In summary, this trial provides highly promising initial data on TB31F, the first malaria transmission-blocking monoclonal antibody to be assessed in humans. Its safety, tolerability, and high efficacy at interrupting human-to-mosquito transmission mark TB31F out to be an important potential tool for malaria control and elimination, advocating for its further assessment in naturally infected gametocyte carriers.

## Data sharing

The study protocol and statistical analysis plan can be accessed via ClinicalTrials.gov, NCT04238689. Deidentified participant data that underlie the results reported in this Article will be made available upon request. Proposals should be directed to the corresponding author. Proposals will be reviewed and approved by the sponsor, investigators, and collaborators on the basis of scientific merit. After approval of a proposal, data requestors will need to sign a data access agreement. Data can be requested indefinitely.

## Declaration of interests

We declare no competing interests.
