# Supplementary material for: Safety, tolerability, and Plasmodium falciparum transmission-reducing activity of monoclonal antibody TB31F: a single-centre, open-label, first-in-human, dose-escalation, phase 1 trial in healthy malaria-naive adults
Source: Lancet Infect Dis. 2022 Nov;22(11):1596–605. doi: 10.1016/S1473-3099(22)00428-5 (PMC9605874; doi:10.1016/S1473-3099(22)00428-5)
Supplement: Supplementary appendix [file mmc1.pdf]

THE LANCET
Infectious Diseases

Supplementary appendix

This appendix formed part of the original submission and has been peer reviewed. We post it as supplied by the authors.

Supplement to: van der Boor SC, Smit MJ, van Beek SW, et al. Safety, tolerability, and Plasmodium falciparum transmission-reducing activity of monoclonal antibody TB31F: a single-centre, open-label, first-in-human, dose-escalation, phase 1 trial in healthy malaria-naive adults. *Lancet Infect Dis* 2022; published online Aug 10. https://doi.org/10.1016/S1473-3099(22)00428-5.

Supplementary appendix

Safety, tolerability and Plasmodium falciparum transmission-reducing activity of monoclonal antibody TB31F: an open-label, first-in-human, trial in healthy malaria-naïve adults

Table of Contents

APPENDIX FILE S1: AMINO ACID SEQUENCE OF TB31F HEAVY CHAIN AND LIGHT CHAIN 2
APPENDIX FILE S2: ANTI-DRUG ANTIBODY LEVELS 3
APPENDIX FIGURE 1 – TB31F CONCENTRATION PER DOSAGE GROUP OVER TIME. 4
APPENDIX FIGURE 2 – DOSE-PROPORTIONALITY TB31F. 5
APPENDIX FIGURE 3 – DEVIANT DATA OF PARTICIPANT 5·5. 6
APPENDIX FIGURE 4 – TRANSMISSION-REDUCING ACTIVITY OF 10 MG/KG TB31F IV AGAINST P. FALCIPARUM ISOLATE NF135 OVER TIME.. 7
APPENDIX FIGURE 5 – FUNCTIONAL ACTIVITY OF TB31F OVER TIME PERFORMED AT LABORATORY OF MALARIA AND VECTOR RESEARCH, NIAID, NIH. 8
APPENDIX FIGURE 6 – PROJECTED IMPACT OF TB31F HALF-LIFE EXTENSION.................................. 9
APPENDIX TABLE 1 – TB31F INFUSION RATES 10
APPENDIX TABLE 2 – TB31F INFUSION TIME 11
APPENDIX TABLE 3 – GRADING OF LOCAL SOLICITED ADVERSE EVENTS FOLLOWING MAB TB31F ADMINISTRATION.. 12
APPENDIX TABLE 4 – GRADING OF SYSTEMIC SOLICITED ADVERSE EVENTS FOLLOWING MAB TB31F ADMINISTRATION.. 12
APPENDIX TABLE 5 – GRADING OF LABORATORY ABNORMALITIES.. 13
APPENDIX TABLE 6 – ANY ADVERSE EVENT, PER GROUP 13
APPENDIX TABLE 7 – UNSOLICITED ADVERSE EVENTS, PER GROUP 14
APPENDIX TABLE 8 – POSSIBLY, PROBABLY, DEFINITELY RELATED ADVERSE EVENTS 15
APPENDIX TABLE 9 – UNLIKELY RELATED, NOT RELATED ADVERSE EVENTS.................................... 17
APPENDIX TABLE 10 – LABORATORY ABNORMALITIES 20
APPENDIX TABLE 11 – TB31F PHARMACOKINETIC PARAMETERS 31

Appendix file S1: Amino Acid Sequence of TB31F Heavy Chain and Light Chain

The amino acid sequences of TB31F reported here was described in Kundu et al[14] (2018) as version "HC2LC1". The TB31F drug product is formulated in 10 mM histidine, 150 mM sodium chloride, 10% (w/v) sucrose, 25 mM arginine, 0.01% (w/v) polysorbate 20, pH 6.0 at a target concentration of 50 mg/mL and met a set of pre-determined specifications for safety, purity, identity, quantity, potency, stability, and toxicity measurements.

Heavy chain

EVQLVESGGGLVQPGGSLRLSCAASGFTFNNYWMSWVRQAPGKGLEWVASISNIGGTIYYPDSVKGRFTISRDS
AKNSLYLQMNSLRAEDTAVYYCTRDLRMSDYFDYWGQGTLVTVSSASTKGPSVFPLAPSSKSTSGGTAALGCL
VKDYFPEPVTVSWNSGALTSGVHTFPAVLQSSGLYSLSSVVTVPSSSLGTQTYICNVNHKPSNTKVDKKVEPKS
CDKTHTCPPCPAPELLGGPSVFLFPPKPKDTLMISRTPEVTCVVVDVSHEDPEVKFNWYVDGVEVHNAKTKPRE
EQYNSTYRVVSVLTVLHQDWLNGKEYKCKVSNKALPAPIEKTISKAKGQPREPQVYTLPPSRDELTKNQVSLTC
LVKGFYPSDIAVEWESNGQPENNYKTTPPVLDSDGSFFLYSKLTVDKSRWQQGNVFSCSVMHEALHNHYTQKS
LSLSPG

Light chain

NFMLTQPHSVSESPGKTVTISCTRSTGNIGSNYVSWYQQRPGSSPTTVIYRDDQRPSGVPDRFSGSIDRSSNSASL
TISGLKTEDEADYYCHSYSTGMYIFGGGTKLTVLGQPKAAPSVTLFPPSSEELQANKATLVCLVSDFYPGAVTV
AWKADGSPVKVGVETTKPSKQSNNKYAASSYLSLTPEQWKSHRSYSCRVTHEGSTVEKTVAPAECS

N = N-linked glycosylation site

Appendix file S2: Anti-drug antibody levels

Anti-Drug antibodies (ADA) assay utilized a tiered approach where samples were first screened to determine whether they were positive or negative for the presence of ADA (Tier 1). The assay format was a bridging ELISA where the ELISA microplate wells were coated with 1·25 µg/mL purified TB31F followed by blocking wells with PBS/Casein. The formation of the Anti-TB31F "bridge" was assessed by the sequential incubation of serum test samples or controls with TB31F that had been conjugated to Biotin. Then, wells were incubated with Streptavidin-HRP and finally developed with TMB substrate before stopping and reading absorbance at 450nm on a plate reader. Samples that had been identified as positive in Tier 1 screening assay were then confirmed as being specific to the drug product in a Tier 2 confirmatory assay. This assay was performed using the same Tier 1 protocol with the exception that the serum test samples were incubated with unlabeled TB31F and tested in a 5-point 2-fold dilution series (TB31F concentration range 0·78-1·25µg/mL). If anti-TB31F antibodies had been present in the test samples the positive signal should have been competed away in the presence of increasing concentrations of TB31F. Affinity-purified rabbit-anti-TB31F polyclonal antibodies were used as the positive control.

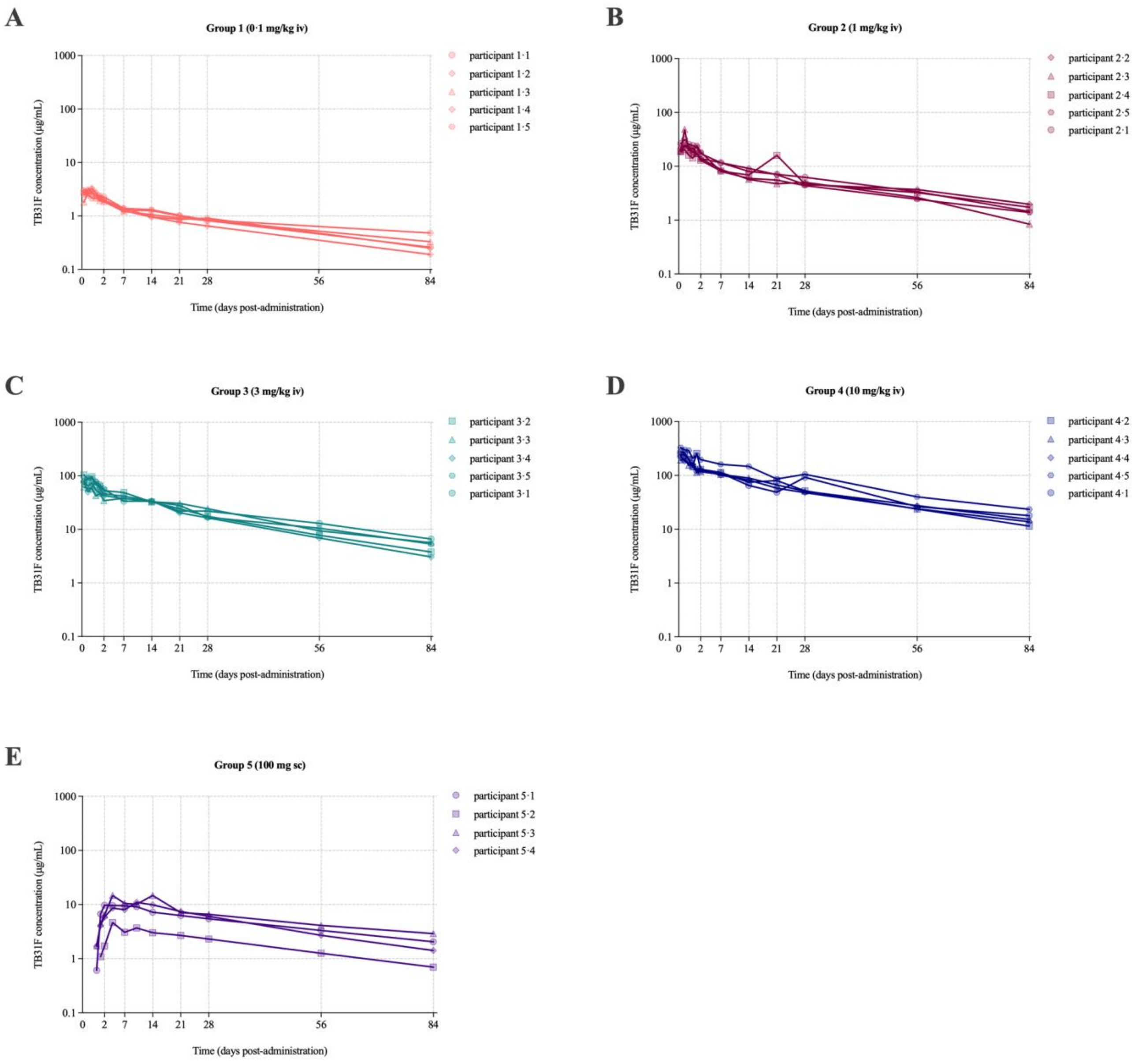

Appendix figure 1 – TB31F concentration per dosage group over time. Each line represents a participant. A: group 1 (0·1 mg/kg iv). B: group 2 (1 mg/kg iv). C: group 3 (3 mg/kg iv). D: group 4 (10 mg/kg iv). E: group 5 (100 mg sc). For group 1, serum TB31F concentrations were unavailable for one participant at day 28 and all five participants at day 56. One participant in group 5 had no detectable or very low concentrations of TB31F at any time point (appendix figure 3A). iv = intravenous. sc = subcutaneous.

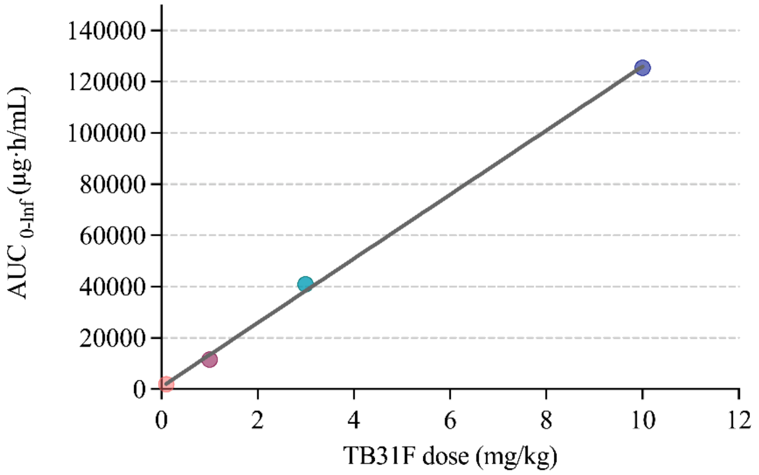

Appendix figure 2 – Dose-proportionality TB31F. Geometric means of the intravenous groups (0·1 mg/kg. 1 mg/kg, 3 mg/kg and 10 mg/kg) are shown. The administered doses are accompanied by proportional increases in measure of exposure ($AUC_{0\text{-Inf}}$). $AUC_{0\text{-Inf}}$ = area under the concentration-time curve from time zero to infinity.

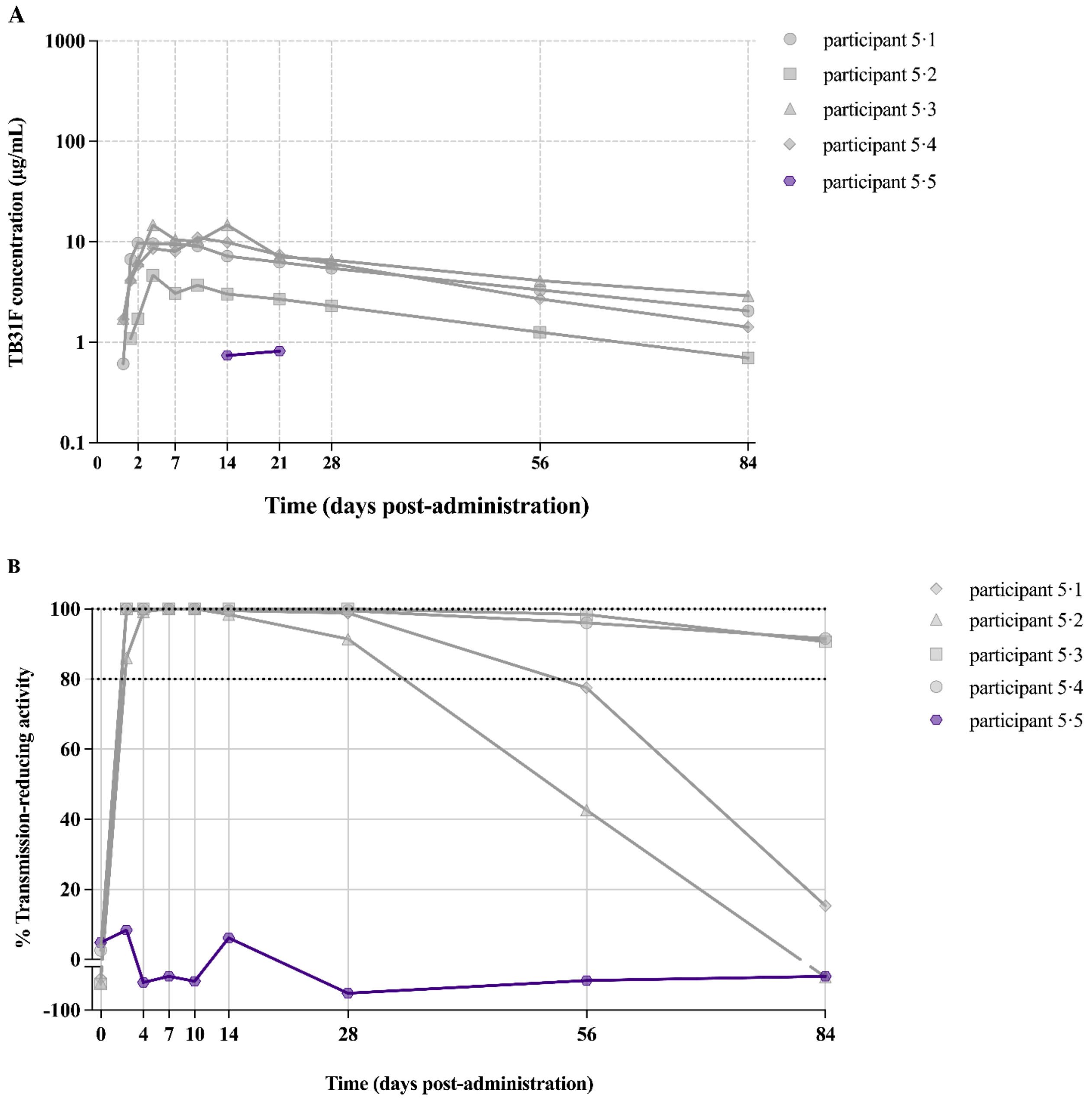

Appendix figure 3 – Deviant data of participant 5·5. A: TB31F concentration over time in participants of group 5 (100 mg sc). Each line represents a participant. Deviant data of participant 5·5 is shown in purple. B: Transmission-reducing activity of TB31F sc against *P. falciparum* isolate NF54 over time in participants of group 5. Transmission-reducing activity (TRA) was calculated by comparing the reduction of oocyst counts using participant sera compared to pooled naïve serum in SMFA. The horizontal dashed lines represent the 100% and 80% TRA threshold. Deviant data of participant 5·5 is shown in purple. iv = intravenous. EOI = end of infusion.

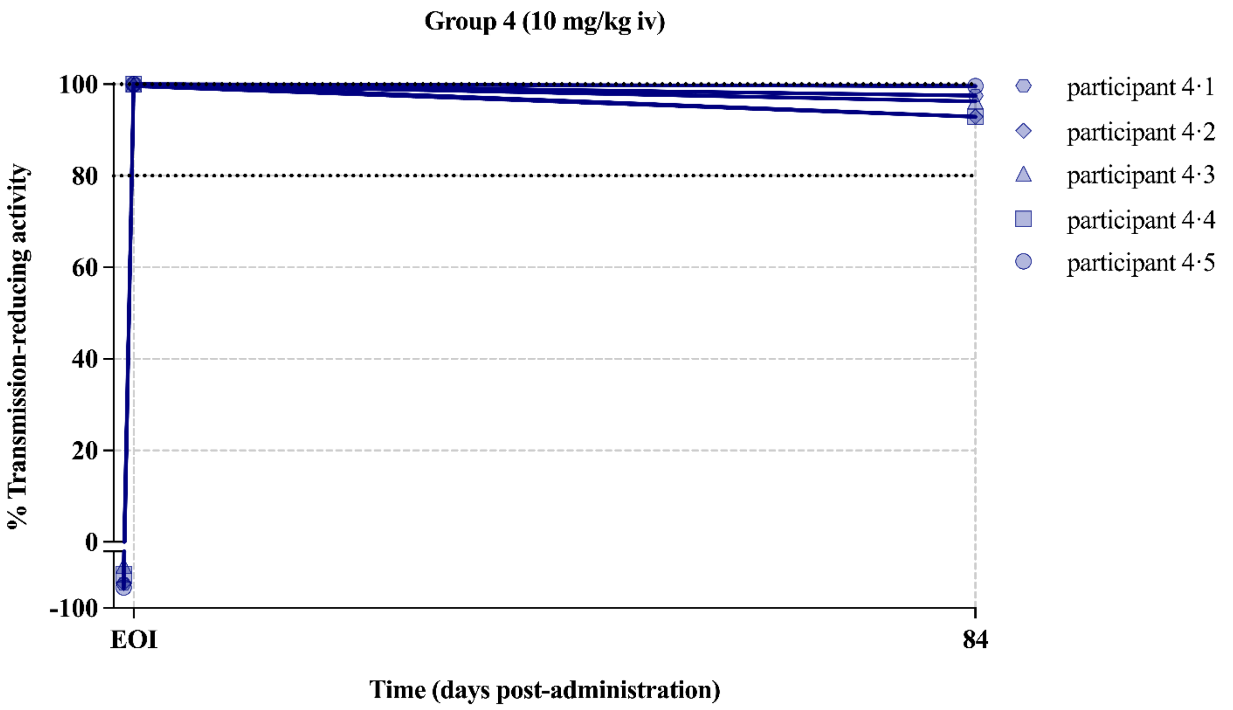

Appendix figure 4 – Transmission-reducing activity of 10 mg/kg TB31F iv against P. falciparum isolate NF135 over time. Transmission-reducing activity (TRA) was calculated by comparing the reduction of oocyst counts using participant sera compared to pooled naïve serum in SMFA. Each line represents a participant from group 4 (10 mg/kg iv). The horizontal dashed lines represent the 100% and 80% TRA threshold. iv = intravenous. EOI = end of infusion.

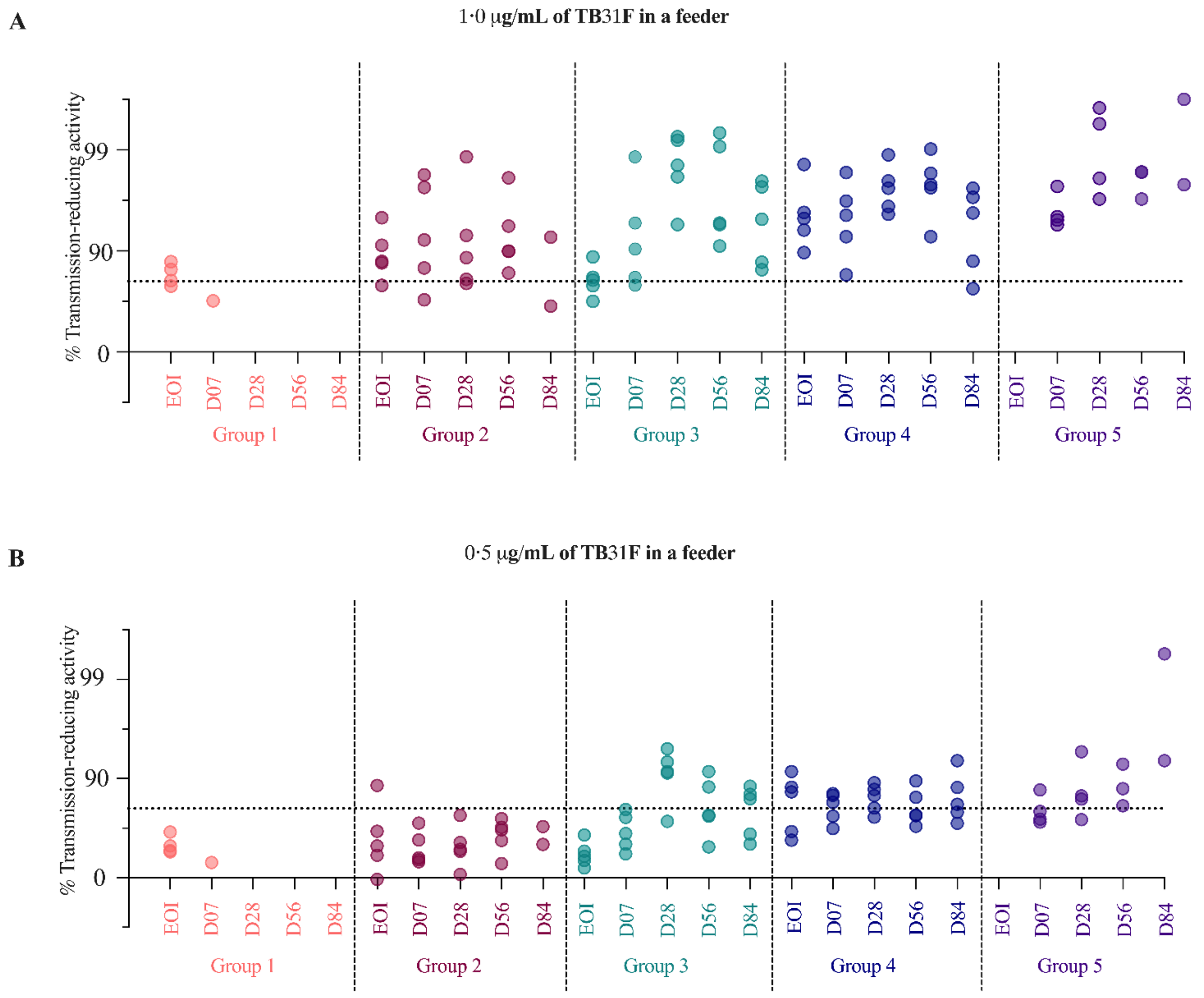

Appendix figure 5 – Functional activity of TB31F over time performed at Laboratory of Malaria and Vector Research, NIAID, NIH. Functional activity of TB31F was tested in SMFA at a fixed concentration of total IgGs (3·75 mg/mL) with complement. IgG was purified using Protein G columns and diluted with naïve human IgG to reach 0·5 or 1 µg/mL TB31F concentration in 3·75 mg/mL total IgG. Multiple samples were not tested as TB31F concentration in purified IgGs were too low to test at 1 µg/mL of TB31F in SMFA. The dashed line represents 80% transmission-reducing activity. D = day. EOI = end of infusion.

A

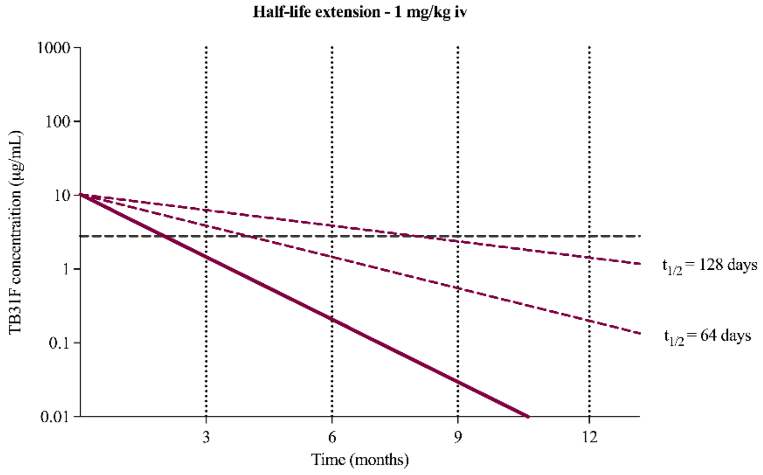

B

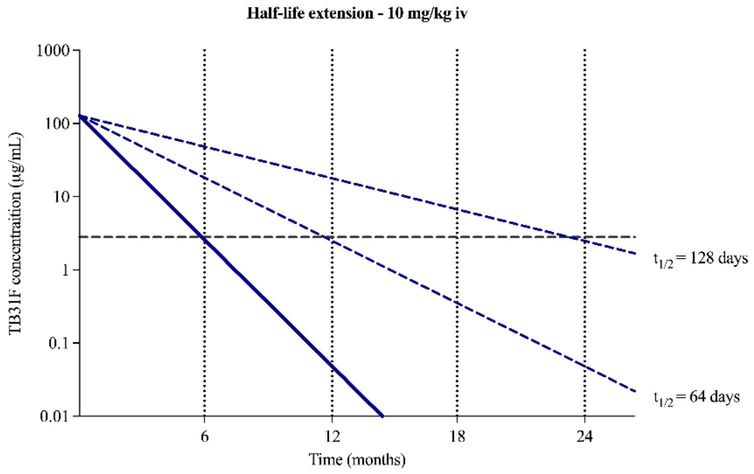

Appendix figure 6 – Projected impact of TB31F half-life extension. Three scenarios for two TB31F dosages (1 mg/kg and 10 mg/kg iv, figure A and B respectively) are shown: the current half-life of 32 days, a two-fold half-life extension to 64 days, and a fourfold extension to 128 days. The horizontal dashed line represents the estimated IC_{80}. iv = intravenous. sc = subcutaneous.

Appendix table 1 – TB31F infusion rates

	Group 1		Group 2		Group 3		Group 4	
**Dose TB31F**	**0·1mg/kg**		**1mg/kg**		**3mg/kg**		**10mg/kg**	
0min	50	mg/h	50	mg/h	100	mg/h	200	mg/h
+30min			100	mg/h	200	mg/h	300	mg/h
+60min			100	mg/h	300	mg/h	400	mg/h
+90min			100	mg/h	300	mg/h	500	mg/h
+120min					300	mg/h	500	mg/h
+150min							500	mg/h
+180min							500	mg/h

Appendix table 2 – TB31F infusion time

Participant	Group	TB31F administered (mg)	Total infusion time (hh:mm)
1·1	1	7·2	00:09
1·2	1	7·7	00:10
1·3	1	6·0	00:07
1·4	1	8·5	01:11
1·5	1	7·4	00:08
2·1	2	74·0	00:54
2·2	2	69·0	00:53
2·3	2	91·0	01:06
2·4	2	69·0	00:52
2·5	2	74·0	00:53
3·1	3	243·0	01:14
3·2	3	159·0	00:57
3·3	3	258·0	01:14
3·4	3	165·0	00:57
3·5	3	252·0	01:17
4·1	4	630·0	01:41
4·2	4	630·0	01:38
4·3	4	740·0	01:54
4·4	4	660·0	01:45
4·5	4	920·0	02:14
5·1	5	100·0	N/A
5·2	5	100·0	N/A
5·3	5	100·0	N/A
5·4	5	100·0	N/A
5·5	5	100·0	N/A

Appendix table 3 – Grading of local solicited adverse events following mAb TB31F administration

Local adverse event	Mild (Grade 1)	Moderate (Grade 2)	Severe (Grade 3)	Potentially Life-threatening (Grade 4)
Pain	Does not interfere with activity	Repeated use of non-narcotic pain reliever > 24 hours or interferes with activity	Any use of narcotic pain reliever or prevents daily activity	Emergency room (ER) visit or hospitalization
Erythema/Swelling	2.5 - 5 cm and does not interfere with activity	5.1 – 10 cm or interferes with activity	> 10 cm or prevents daily activity	Necrosis or exfoliative dermatitis

Appendix table 4 – Grading of systemic solicited adverse events following mAb TB31F administration

Systemic adverse event	Mild (Grade 1)	Moderate (Grade 2)	Severe (Grade 3)	Potentially Life-threatening (Grade 4)
Fever (measured orally)	38·0 – 38·4 (°C)<br>100·4 – 101·1 (°F)	38·5 – 38·9 (°C)<br>101·2 – 102·0 (°F)	39·0 – 40 (°C)<br>102·1 – 104 (°F)	>40 (°C)
Chills	No interference with activity	Some interference with activity	Significant; prevents daily activity	ER visit or hospitalization
Fatigue	No interference with activity	Some interference with activity	Significant; prevents daily activity	ER visit or hospitalization
Headache	No interference with activity	Repeated use of non- narcotic pain reliever > 24 hours or some interference with activity	Significant; any use of narcotic pain reliever or prevents daily activity	ER visit or hospitalization
Myalgia	No interference with activity	Repeated use of non- narcotic pain reliever > 24 hr or some interference with activity	Significant; prevents daily activity	ER visit or hospitalization

Appendix table 5 – Grading of Laboratory Abnormalities

	Grade	Range
Haemoglobin, male (mmol/L)	1	7·8-8·3
	2	6·5-7·7
	3	5·2-6·4
Haemoglobin, female (mmol/L)	1	6·8-7·3
	2	5·9-6·7
	3	5·0-5·8
Increase in Leukocytes (cells/mm3)	1	11,000 – 15,000
	2	15,001 - 20,000
	3	20,001 - 25,000
Decrease in Leukocytes (cells/mm3)	1	2,500 - 3,500
	2	1,500 - 2499
	3	1,000 -1,499
Decrease in Platelets (cells/mm3)	1	125,000 – 140·000
	2	100,000 - 124,000
	3	25,000 – 99,000
Creatinine (mg/dL)	1	1·5-1·7
	2	1·8-2·0
	3	2·1-2·5
Transaminases (AST) (U/L)	1	1·1-2·5 x ULN
	2	2·6-5 x ULN
	3	5·1-10 x ULN
Transaminases (ALT) (U/L)	1	1·1- 2·5 x ULN
	2	2·6 - 5 x ULN
	3	5·1-10 x ULN

AST = Aspartate aminotransferase. ALT = alanine aminotransferase. ULN = upper limit of normal.

Appendix table 6 – Any adverse event, per group

		**Group 1 0·1 mg/kg iv**		**Group 2 1 mg/kg iv**		**Group 3 3 mg/kg iv**		**Group 4 10 mg/kg iv**		**Group 5 100 mg sc**		**All**	
		#Subjects	%	#Subjects	%	#Subjects	%	#Subjects	%	#Subjects	%	#Subjects	%
**Local solicited AE (D0-D7)**	**Grade 1**	0	NA	1/5	20	1/5	NA	0	NA	4/5	80	6/25	24
	**Grade 2**	0	NA	0	NA	0	NA	0	NA	0	NA	0	NA
	**Grade 3**	0	NA	0	NA	0	NA	0	NA	0	NA	0	NA
	**Any grade**	0	NA	1/5	20	1/5	NA	0	NA	4/5	80	6/25	24
**Systemic solicited AE (D0-D28)**	**Grade 1**	4/5	80	3/5	60	1/5	20	5/5	100	2/5	40	15/25	60
	**Grade 2**	3/5	60	0	NA	0	NA	1/5	20	0	NA	4/25	16
	**Grade 3**	0	NA	0	NA	0	NA	0	NA	0	NA	0	NA
	**Any grade**	5/5	100	3/5	60	1/5	20	5/5	100	2/5	40	16/25	64
**Unsolicited AE (D0-D84)**	**Grade 1**	1/5	20	0	NA	0	NA	1/5	40	0	NA	2/25	8
	**Grade 2**	1/5	20	0	NA	0	NA	0	NA	0	NA	1/25	4
	**Grade 3**	0	NA	0	NA	0	NA	0	NA	0	NA	0	NA
	**Any grade**	2/5	40	0	NA	0	NA	1/5	40	0	NA	3/25	12
**Any AE (D0-D84)**	**Grade 1**	4/5	80	4/5	80	2/5	40	5/5	100	4/5	80	19/25	76
	**Grade 2**	3/5	60	0	NA	0	NA	1/5	20	0	NA	4/25	16
	**Grade 3**	0	NA	0	NA	0	NA	0	NA	0	NA	0	NA
	**Any grade**	5/5	100	4/5	80	2/5	40	5/5	100	4/5	80	20/25	80

Possibly, probably and definitely related adverse events reported per group. iv = intravenous. sc = subcutaneous. AE = adverse event. D = day. NA = not applicable.

Appendix table 7 – Unsolicited adverse events, per group

		Group 1 0.1 mg/kg iv		Group 2 1 mg/kg iv		Group 3 3 mg/kg iv		Group 4 10 mg/kg iv		Group 5 100 mg sc		All	
		#Subjects	%	#Subjects	%	#Subjects	%	#Subjects	%	#Subjects	%	#Subjects	%
**Depressed feeling**	**Grade 1**	0	NA	0	NA	0	NA	0	NA	0	NA	0	NA
	**Grade 2**	1/5	20	0	NA	0	NA	0	NA	0	NA	1/5	4
	**Grade 3**	0	NA	0	NA	0	NA	0	NA	0	NA	0	NA
**Diarrhea**	**Grade 1**	1/5	20	0	NA	0	NA	0	NA	0	NA	1/5	4
	**Grade 2**	0	NA	0	NA	0	NA	0	NA	0	NA	0	NA
	**Grade 3**	0	NA	0	NA	0	NA	0	NA	0	NA	0	NA
**Sore throat**	**Grade 1**	0	NA	0	NA	0	NA	0	NA	0	NA	0	NA
	**Grade 2**	0	NA	0	NA	0	NA	0	NA	0	NA	0	NA
	**Grade 3**	0	NA	0	NA	0	NA	0	NA	0	NA	0	NA
**Stomach ache**	**Grade 1**	0	NA	0	NA	0	NA	1/5	20	0	NA	1/5	4
	**Grade 2**	0	NA	0	NA	0	NA	0	NA	0	NA	0	NA
	**Grade 3**	0	NA	0	NA	0	NA	0	NA	0	NA	0	NA
**Headache**	**Grade 1**	0	NA	1/5	20	0	NA	0	NA	0	NA	0	NA
	**Grade 2**	0	NA	0	NA	0	NA	0	NA	0	NA	0	NA
	**Grade 3**	0	NA	0	NA	0	NA	0	NA	0	NA	0	NA

Possibly, probably and definitely related adverse events reported per group. iv = intravenous. sc = subcutaneous. NA = not applicable.

Appendix table 8 – Possibly, probably, definitely related adverse events

Record ID	Group	Adverse Event	ICD-10	Onset	End	Time from administration	Ongoing	Severity	Causality	Type	Medication
1·1	1	Fatigue	R53	17/02/2020 14:00	18/02/2020 07:00	0·19	No	Moderate	possibly related	systemic solicited	No
1·2	1	Myalgia	M79·19	20/02/2020 10:04	21/02/2020 10:04	0·05	No	Mild	possibly related	systemic solicited	No
1·2	1	Headache	R51	20/02/2020 21:30	21/02/2020 06:30	0·52	No	Mild	possibly related	systemic solicited	No
1·2	1	Fatigue	R53	21/02/2020 07:00	21/02/2020 13:00	0·92	No	Mild	possibly related	systemic solicited	No
1·2	1	Fatigue	R53	21/02/2020 13:00	21/02/2020 18:00	1·17	No	Moderate	possibly related	systemic solicited	No
1·2	1	Depressed feeling	F38	21/02/2020 13:00	21/02/2020 18:00	1·17	No	Moderate	possibly related	unsolicited	No
1·3	1	Fatigue	R53	25/02/2020 09:00	26/02/2020 11:00	1·01	No	Mild	probably related	systemic solicited	No
1·4	1	Fatigue	R53	27/02/2020 10:00	27/02/2020 23:00	0·02	No	Mild	possibly related	systemic solicited	No
1·4	1	Diarrhoea	R19·4	27/02/2020 18:30	27/02/2020 22:30	0·38	No	Mild	possibly related	unsolicited	No
1·4	1	Sore throat	J02·3	01/03/2020 07:00	01/03/2020 22:00	2·90	No	Mild	possibly related	unsolicited	No
1·5	1	Fatigue	R53	03/03/2020 17:00	04/03/2020 06:00	1·33	No	Moderate	possibly related	systemic solicited	No
1·5	1	Fatigue	R53	04/03/2020 06:00	04/03/2020 20:00	1·87	No	Mild	possibly related	systemic solicited	No
2·1	2	Fatigue	R53	06/07/2020 10:20	06/07/2020 10:45	0·04	No	Mild	possibly related	systemic solicited	No
2·1	2	Redness at injection site	L53·8	07/07/2020 14:00	08/07/2020 08:00	1·19	No	Mild	probably related	local solicited	No
2·2	2	Headache	R51	09/07/2020 15:50	09/07/2020 17:00	0·29	No	Mild	possibly related	systemic solicited	No
2·3	2	Fatigue	R50·9	13/07/2020 10:30	13/07/2020 13:45	0·04	No	Mild	possibly related	systemic solicited	No
2·5	2	Headache	R51	18/08/2020 08:00	18/08/2020 22:00	32·86	No	Mild	possibly related	systemic solicited	Yes
2·5	2	Headache	R51	25/08/2020 08:00	25/08/2020 22:00	39·86	No	Mild	possibly related	systemic solicited	No
2·5	2	Headache	R51	28/08/2020 08:00	28/08/2020 22:00	42·86	No	Mild	possibly related	systemic solicited	Yes
3·2	3	Pain at injection site	M79·68	07/08/2020 07:30	07/08/2020 11:48	0·93	No	Mild	possibly related	local solicited	No
3·3	3	Fatigue	R53	10/08/2020 10:52	11/08/2020 08:00	0·05	No	Mild	possibly related	systemic solicited	No

4·1	4	Headache	R51	30/08/2020 09:00	30/08/2020 11:15	2·99	No	Mild	possibly related	systemic solicited	Yes
4·1	4	Headache	R51	31/08/2020 11:30	31/08/2020 14:15	4·10	No	Mild	possibly related	systemic solicited	No
4·2	4	Fatigue	R53	31/08/2020 14:15	01/09/2020 08:00	0·19	No	Mild	possibly related	systemic solicited	No
4·3	4	Headache	R51	03/09/2020 19:30	03/09/2020 20:00	0·43	No	Mild	probably related	systemic solicited	Yes
4·3	4	Fatigue	R53	04/09/2020 13:00	04/09/2020 17:00	1·16	No	Moderat e	possibly related	systemic solicited	No
4·3	4	Headache	R51	10/09/2020 22:00	11/09/2020 02:00	7·53	No	Moderat e	possibly related	systemic solicited	Yes
4·4	4	Fever	R50·9	07/09/2020 17:27	07/09/2020 23:30	0·32	No	Mild	possibly related	systemic solicited	No
4·4	4	Fatigue	R53	08/09/2020 22:30	09/09/2020 08:00	1·53	No	Mild	possibly related	systemic solicited	No
4·5	4	Headache	R51	07/09/2020 17:00	07/09/2020 17:59	0·09	No	Mild	probably related	systemic solicited	No
4·5	4	Stomachache	R10·4	08/09/2020 10:00	08/09/2020 11:00	0·80	No	Mild	possibly related	unsolicited	No
4·5	4	Headache	R51	19/09/2020 19:40	19/09/2020 20:30	12·20	No	Mild	possibly related	systemic solicited	No
4·5	4	Headache	R51	02/10/2020 23:45	03/10/2020 00:45	25·37	No	Mild	possibly related	systemic solicited	No
5·1	5	Pain during injection	M79·6 8	30/11/2020 09:12	30/11/2020 09:14	0·00	No	Mild	possibly related	local solicited	No
5·2	5	Pain during injection	M79·6 8	03/12/2020 09:23	03/12/2020 09:23	0·00	No	Mild	possibly related	local solicited	No
5·2	5	Fatigue	R53	05/12/2020 16:00	05/12/2020 22:00	2·28	No	Mild	possibly related	systemic solicited	No
5·4	5	Pain	M79·6 8	10/12/2020 09:41	10/12/2020 09:43	0·00	No	Mild	probably related	local solicited	No
5·5	5	Pain	M79·6 8	10/12/2020 10:40	10/12/2020 10:42	0·00	No	Mild	probably related	local solicited	No
5·5	5	Fatigue	R53	10/12/2020 11:39	10/12/2020 16:42	0·04	No	Mild	possibly related	systemic solicited	No

Appendix table 9 – Unlikely related, not related adverse events

Record ID	Group	Adverse Event	ICD-10	Onset	End	Time from administration	Ongoing	Severity	Causality	Type	Medication
1·2	1	Flatulence	R14	29/02/2020 07:00	29/02/2020 23:00	8·92	No	Mild	unlikely related	unsolicited	No
1·2	1	Diarrhoea	R19·4	03/03/2020 18:30	04/03/2020 23:00	12·40	No	Mild	unlikely related	unsolicited	No
1·2	1	Heartburn	K21·9	09/03/2020 13:00	09/03/2020 22:00	18·17	No	Mild	unlikely related	unsolicited	Yes
1·2	1	Headache	R51	09/03/2020 14:00	09/03/2020 20:00	18·21	No	Mild	unlikely related	systemic solicited	No
1·2	1	Headache	R51	13/03/2020 08:15	13/03/2020 10:30	21·97	No	Mild	unlikely related	systemic solicited	No
1·2	1	Headache	R51	08/05/2020 20:00	08/05/2020 23:59	78·46	No	Mild	unlikely related	systemic solicited	No
1·3	1	Sore throat	J02·3	29/02/2020 08:30	29/02/2020 09:30	4·99	No	Mild	unlikely related	unsolicited	No
1·3	1	Headache	R51	08/03/2020 19:25	08/03/2020 23:30	13·44	No	Mild	unlikely related	systemic solicited	No
1·3	1	Sore Throat	J02·3	14/04/2020 08:00	14/04/2020 08:30	49·97	No	Mild	unlikely related	unsolicited	No
1·3	1	Cough	R05	20/04/2020 08:00	NA	55·97	Yes	Mild	unlikely related	unsolicited	No
1·4	1	Sore throat	J02·3	07/03/2020 08:00	20/03/2020 08:00	8·94	No	Mild	unlikely related	unsolicited	No
1·4	1	Distortion left ankle	S93	09/04/2020 08:00	NA	41·94	Yes	Mild	not related	unsolicited	Yes
1·4	1	Ingrown toenail	L60·0	13/05/2020 08:00	18/05/2020 17:00	75·94	No	Mild	not related	unsolicited	Yes
1·4	1	Headache	R51	15/04/2020 15:00	15/04/2020 19:00	48·23	No	Mild	unlikely related	systemic solicited	Yes
1·4	1	Headache	R51	20/04/2020 14:00	20/04/2020 18:00	53·19	No	Mild	unlikely related	systemic solicited	Yes
1·5	1	Hematoma	T14·0	03/03/2020 06:00	11/03/2020 08:00	0·87	No	Mild	not related	local solicited	No
1·5	1	Dry cough	R05	05/03/2020 16:00	06/03/2020 23:00	3·29	No	Mild	unlikely related	unsolicited	Yes
1·5	1	Distortion/bruised right hand	S63·7	14/04/2020 12:00	19/04/2020 22:00	43·12	No	Moderate	not related	unsolicited	Yes
2·2	2	Erythema (due to tape)	L53·8	09/07/2020 17:00	09/07/2020 22:00	0·34	No	Mild	not related	unsolicited	No
2·5	2	Myalgia	M79·19	19/07/2020 09:00	22/07/2020 09:00	2·90	No	Mild	unlikely related	systemic solicited	No
2·5	2	Strained ankle (left)	S90	09/08/2020 20:30	23/08/2020 08:00	24·38	No	Moderate	not related	unsolicited	Yes
2·5	2	Urinary tract infection	N39·0	31/08/2020 08:00	15/09/2020 08:00	45·86	No	Mild	not related	unsolicited	Yes
3·4	3	Migraine attack	G43	09/09/2020 10:00	09/09/2020 15:00	27·04	No	Moderate	unlikely related	unsolicited	Yes
3·5	3	Headache	R51	14/08/2020 08:30	14/08/2020 08:40	0·89	No	Mild	unlikely related	systemic solicited	No

4·1	4	Otalgia	H92·0	27/08/2020 11:45	27/08/2020 11:50	0·11	No	Mild	not related	unsolicited	No
4·1	4	Headache	R51	27/09/2020 11:00	28/09/2020 07:30	31·08	No	Mild	not related	systemic solicited	Yes
4·1	4	Headache	R51	28/09/2020 07:30	28/09/2020 12:00	31·93	No	Moderate	not related	systemic solicited	Yes
4·1	4	Headache	R51	13/10/2020 15:30	13/10/2020 18:00	47·27	No	Mild	unlikely related	unsolicited	No
4·1	4	Sore throat	J02·3	13/10/2020 08:00	16/10/2020 00:00	46·95	No	Mild	unlikely related	unsolicited	No
4·1	4	Headache	R51	16/10/2020 12:00	16/12/2020 17:00	50·12	No	Mild	unlikely related	systemic solicited	No
4·2	4	Stomach ache	R10·4	31/08/2020 18:00	01/09/2020 08:00	0·35	No	Mild	unlikely related	unsolicited	No
4·2	4	Patellofemoral pain syndrome	M22·2	07/09/2020 11:00	NA	7·06	Yes	Mild	not related	unsolicited	Yes
4·2	4	Headache	R51	22/09/2020 15:00	24/09/2020 23:00	22·22	No	Mild	unlikely related	systemic solicited	Yes
4·3	4	Vasovagal	R42	03/09/2020 14:20	03/09/2020 14:30	0·21	No	Mild	unlikely related	unsolicited	No
4·3	4	Headache	R51	04/09/2020 00:00	04/09/2020 02:00	0·61	No	Mild	unlikely related	systemic solicited	Yes
4·3	4	Headache	R51	09/09/2020 22:00	10/09/2020 01:00	6·53	No	Moderate	not related	systemic solicited	No
4·3	4	Upper respiratory tract infection	J06·9	24/09/2020 23:00	26/09/2020 09:00	21·57	No	Moderate	unlikely related	unsolicited	Yes
4·3	4	Upper respiratory tract infection	J06·9	26/09/2020 09:00	29/09/2020 09:00	22·99	No	Mild	unlikely related	unsolicited	Yes
4·3	4	Headache	R51	15/10/2020 14:00	15/10/2020 17:00	42·20	No	Mild	unlikely related	unsolicited	Yes
4·3	4	Headache	R51	27/10/2020 21:00	28/10/2020 01:00	54·49	No	Mild	unlikely related	unsolicited	Yes
4·3	4	Headache	R51	11/11/2020 14:00	11/11/2020 18:00	69·20	No	Mild	not related	systemic solicited	Yes
4·3	4	Stomach cramps	N94	20/11/2020 14:00	22/11/2020 19:00	78·20	No	Mild	not related	unsolicited	Yes
4·4	4	Myalgia	M79·19	07/09/2020 11:43	07/09/2020 12:43	0·08	No	Mild	unlikely related	systemic solicited	No
4·4	4	Vasovagal reaction	R42	07/09/2020 12:33	07/09/2020 12:43	0·12	No	Mild	unlikely related	unsolicited	No
4·4	4	Vasovagal reaction	R42	07/09/2020 14:20	07/09/2020 14:28	0·19	No	Mild	unlikely related	unsolicited	No
4·4	4	Headache	R51	10/09/2020 08:30	11/09/2020 14:00	2·95	No	Moderate	not related	systemic solicited	Yes
4·4	4	Headache	R51	18/09/2020 17:30	18/09/2020 19:00	11·32	No	Mild	unlikely related	systemic solicited	Yes
4·4	4	Vertigo	R42	23/09/2020 12:00	23/09/2020 17:00	16·10	No	Mild	unlikely related	unsolicited	No
4·4	4	Headache	R51	02/10/2020 13:00	03/10/2020 00:00	25·14	No	Mild	unlikely related	systemic solicited	No
4·4	4	Headache	R51	07/10/2020 07:00	10/10/2020 19:00	29·89	No	Moderate	not related	systemic solicited	Yes

4·4	4	Headache	R51	28/10/2020 16:00	28/10/2020 23:00	51·26	No	Mild	unlikely related	systemic solicited	No
4·4	4	Headache	R51	07/11/2020 09:00	07/11/2020 11:00	60·97	No	Mild	unlikely related	systemic solicited	Yes
4·4	4	Headache	R51	14/11/2020 21:00	14/11/2020 00:00	68·47	No	Mild	unlikely related	systemic solicited	No
4·4	4	Headache	R51	03/11/2020 16:00	03/11/2020 20:15	57·26	No	Moderate	unlikely related	systemic solicited	Yes
4·4	4	Headache	R51	08/11/2020 09:00	08/11/2020 11:15	61·97	No	Mild	unlikely related	systemic solicited	Yes
4·5	4	Pain right knee	R29·8	26/09/2020 16:30	26/09/2020 17:30	19·07	No	Mild	not related	unsolicited	No
4·5	4	Pain left arm	M79·19	05/11/2020 23:00	06/11/2020 00:30	59·34	No	Moderate	not related	unsolicited	No
4·5	4	Myalgia	M79·19	12/09/2020 08:30	15/11/2020 07:00	4·74	No	Mild	unlikely related	systemic solicited	No
4·5	4	Headache	R51	25/10/2020 20:15	25/10/2020 21:30	48·23	No	Moderate	unlikely related	systemic solicited	No
4·5	4	Headache	R51	13/09/2020 21:00	13/09/2020 21:30	6·26	No	Mild	unlikely related	systemic solicited	No
4·5	4	Chest Pain (right)	R07·3	08/10/2020 10:10	08/10/2020 10:20	30·81	No	Mild	unlikely related	unsolicited	No
4·5	4	Headache	R51	11/10/2020 19:30	12/10/2020 00:30	34·20	No	Mild	unlikely related	systemic solicited	Yes
5·1	5	Headache	R51	01/12/2020 17:00	01/12/2020 20:00	1·33	No	Mild	unlikely related	systemic solicited	No
5·1	5	Myalgia	M79·17	05/12/2020 07:00	05/01/2021 07:00	4·91	No	Mild	unlikely related	systemic solicited	No
5·2	5	Fatigue	R53	03/12/2020 10:28	03/12/2020 20:00	0·05	No	Mild	unlikely related	systemic solicited	No
5·4	5	Fatigue	R53	10/12/2020 09:44	10/12/2020 10:50	0·00	No	Mild	unlikely related	systemic solicited	No
5·4	5	headache	R51	22/12/2020 10:00	23/12/2020 23:00	12·01	No	Mild	unlikely related	systemic solicited	No
5·4	5	Headache	R51	22/12/2020 10:00	23/12/2020 23:00	12·01	No	Mild	unlikely related	systemic solicited	No
5·5	5	Toothache	K08·8	25/12/2020 08:00	27/12/2020 08:00	14·89	No	Mild	not related	unsolicited	Yes
5·5	5	Sore throat	J02·3	30/12/2020 07:00	31/12/2020 23:00	19·85	No	Mild	unlikely related	unsolicited	No

NA: Not applicable

Appendix table 10 – Laboratory abnormalities

Participant	Group	Date & Time of sampling	Time from administration (days)	Abnormality	Value	Normal range	Unit	Clinically Significant	Comments
1·1	1	14/02/2020 10:54	-2·93	Triglycerides	0·66	0·8 – 2·0	mmol/l	no	NA
1·1	1	18/02/2020 08:15	0·95	AST	64	<30	U/l	no	NA
1·1	1	18/02/2020 08:15	0·95	LDH	249	<250	U/l	no	NA
1·1	1	19/02/2020 10:14	2·04	AST	67	<30	U/l	yes	NA
1·1	1	19/02/2020 10:14	2·04	LDH	272	<250	U/l	yes	NA
1·1	1	21/02/2020 10:16	4·04	AST	44	<30	U/l	no	NA
1·1	1	21/02/2020 10:16	4·04	Urea	2·2	2·5 – 7·0	mmol/l	no	NA
1·1	1	24/02/2020 10:11	7·04	Hemoglobin	7·1	7·4 – 9·9	mmol/l	no	NA
1·1	1	24/02/2020 10:11	7·04	Hematocrit	0·36	0·36 – 0·46	l/l	no	NA
1·1	1	24/02/2020 10:11	7·04	Erythrocytes	3·99	4·00 – 5·20	*10^12/l	no	NA
1·1	1	24/02/2020 10:11	7·04	Urea	2	2·5 – 7·0	mmol/l	no	NA
1·1	1	02/03/2020 10:14	14·04	Hemoglobin	7·3	7·4 – 9·9	mmol/l	no	NA
1·1	1	02/03/2020 10:14	14·04	Hematocrit	0·35	0·36 – 0·46	l/l	no	NA
1·1	1	02/03/2020 10:14	14·04	Leukocytes	3·8	4·0 – 11·0	*10^9/l	no	NA
1·1	1	02/03/2020 10:14	14·04	Neutrophils	1·5	2·00 – 7·50	*10^9/l	no	NA
1·1	1	02/03/2020 10:14	14·04	Monocytes	0·29	0·30 – 1·00	*10^9/l	no	NA
1·1	1	09/03/2020 10:34	21·05	Hemoglobin	7·2	7·4 – 9·9	mmol/l	no	NA
1·1	1	09/03/2020 10:34	21·05	Hematocrit	0·36	0·36 – 0·46	l/l	no	NA
1·1	1	16/03/2020 10:06	28·03	Hemoglobin	7	7·4 – 9·9	mmol/l	no	NA
1·1	1	16/03/2020 10:06	28·03	Hematocrit	0·35	0·36 – 0·46	l/l	no	NA
1·1	1	16/03/2020 10:06	28·03	Leukocytes	4	4·0 – 11·0	*10^9/l	no	NA
1·1	1	16/03/2020 10:06	28·03	Neutrophils	1·5	2·00 – 7·50	*10^9/l	no	NA
1·1	1	11/05/2020 10:13	84·04	Leukocytes	3·1	4·0 – 11·0	*10^9/l	no	NA
1·1	1	11/05/2020 10:13	84·04	Neutrophils	1·25	2·00 – 7·50	*10^9/l	no	NA
1·1	1	11/05/2020 10:13	84·04	Monocytes	0·25	0·30 – 1·00	*10^9/l	no	NA
1·2	1	05/03/2020 15:08	14·26	Neutrophils	1·95	2·00 – 7·50	*10^9/l	no	NA

1·2	1	05/03/2020 15:08	14·26	Leukocytes	3·9	4·0 – 11·0	*10^9/l	no	NA
1·2	1	12/03/2020 08:46	20·99	Bilirubin total	21	<17	µmol/l	no	NA
1·2	1	14/05/2020 10:12	84·05	MCHC	21·8	20·0-21·5	mmol/l	no	NA
1·2	1	14/05/2020 10:12	84·05	Bilirubin total	19	<17	µmol/l	no	NA
1·2	1	19/03/2020 08:45	27·99	Leukocytes	4	4·0 – 11·0	*10^9/l	no	NA
1·3	1	23/01/2020 10:55	-31·91	Bilirubin total	20	<17	µmol/l	no	NA
1·3	1	25/02/2020 09:37	1·03	Bilirubin total	17	<17	µmol/l	no	NA
1·3	1	26/02/2020 09:27	2·03	Bilirubin total	17	<17	µmol/l	no	NA
1·4	1	10/02/2020 09:05	-17·01	Hemoglobin	7·2	7·4 – 9·9	mmol/l	no	NA
1·4	1	10/02/2020 09:05	-17·01	Erythrocytes	5·22	4·0 – 5·20	*10^12/l	no	NA
1·4	1	10/02/2020 09:05	-17·01	MCV	72	80 – 100	fl	no	NA
1·4	1	26/02/2020 09:33	-0·99	Hemoglobin	6·7	7·4 – 9·9	mmol/l	no	NA
1·4	1	26/02/2020 09:33	-0·99	Hematocrit	0·35	0·36 – 0·46	l/l	no	NA
1·4	1	26/02/2020 09:33	-0·99	MCV	71	80 – 100	fl	no	NA
1·4	1	28/02/2020 11:58	1·11	Hemoglobin	6·6	7·4 – 9·9	mmol/l	no	NA
1·4	1	28/02/2020 11:58	1·11	Hematocrit	0·34	0·36 – 0·46	l/l	no	NA
1·4	1	28/02/2020 11:58	1·11	MCV	70	80 – 100	fl	no	NA
1·4	1	29/02/2020 12:48	2·14	Hemoglobin	6·5	7·4 – 9·9	mmol/l	no	NA
1·4	1	29/02/2020 12:48	2·14	Hematocrit	0·34	0·36 – 0·46	l/l	no	NA
1·4	1	29/02/2020 12:48	2·14	MCV	70	80 – 100	fl	no	NA
1·4	1	29/02/2020 12:48	2·14	Eosinophils	0·55	<0·50	*10^9/l	no	NA
1·4	1	05/03/2020 14:58	7·23	Hemoglobin	6·4	7·4 – 9·9	mmol/l	no	NA
1·4	1	05/03/2020 14:58	7·23	Hematocrit	0·33	0·36 – 0·46	l/l	no	NA
1·4	1	05/03/2020 14:58	7·23	MCV	70	80 – 100	fl	no	NA
1·4	1	12/03/2020 08:35	13·97	Hemoglobin	6·4	7·4 – 9·9	mmol/l	no	NA
1·4	1	12/03/2020 08:35	13·97	Hematocrit	0·34	0·36 – 0·46	l/l	no	NA
1·4	1	12/03/2020 08:35	13·97	MCV	71	80 – 100	fl	no	NA
1·4	1	19/03/2020 08:39	20·97	Hemoglobin	6·9	7·4 – 9·9	mmol/l	no	NA
1·4	1	19/03/2020 08:39	20·97	Hematocrit	0·36	0·36 – 0·46	l/l	no	NA

1·4	1	19/03/2020 08:39	20·97	MCV	69	80 – 100	fl	no	NA
1·4	1	27/03/2020 17:04	29·32	Hemoglobin	6·8	7·4 – 9·9	mmol/l	no	NA
1·4	1	27/03/2020 17:04	29·32	Hematocrit	0·36	0·36 – 0·46	l/l	no	NA
1·4	1	27/03/2020 17:04	29·32	MCV	69	80 – 100	fl	no	NA
1·4	1	27/03/2020 17:04	29·32	Lymfocytes	3·82	1·0 – 3·5	*10^9/l	no	NA
1·4	1	20/05/2020 10:08	83·03	Hemoglobin	7	7·4 – 9·9	mmol/l	no	NA
1·4	1	20/05/2020 10:08	83·03	Erythrocytes	5·28	4·0 – 5·20	*10^12/l	no	NA
1·4	1	20/05/2020 10:08	83·03	MCV	69	80 – 100	fl	no	NA
1·4	1	20/05/2020 10:08	83·03	Urea	2·1	2·5 – 7	mmol/l	no	NA
1·5	1	03/03/2020 07:25	0·93	Plasmacytoid lymphocytes present	N/A	N/A	N/A	no	NA
1·5	1	03/03/2020 07:25	0·93	Macrotrombocytes present	N/A	N/A	N/A	no	NA
1·5	1	04/03/2020 07:20	1·93	AST	31	<30	U/l	no	NA
1·5	1	09/03/2020 14:50	7·24	Hemoglobin	7·3	7·4 - 9·9	mmol/l	no	NA
1·5	1	16/03/2020 08:20	13·97	Hemoglobin	7·2	7·4 - 9·9	mmol/l	no	NA
1·5	1	16/03/2020 08:20	13·97	Neutrophils	1·58	2 - 7·5	*10^9/l	no	NA
1·5	1	23/03/2020 08:20	20·97	Hemoglobin	7·1	7·4 - 9·9	mmol/l	no	NA
1·5	1	23/03/2020 08:20	20·97	Erythrocytes	3·93	4·0 – 5·2	*10^12/l	no	NA
1·5	1	23/03/2020 08:20	20·97	Neutrophils	1·88	2·0 – 7·5	*10^9/l	no	NA
1·5	1	25/05/2020 10:12	84·05	Neutrophils	1·85	2·00 – 7·50	*10^9/l	no	NA
1·5	1	25/05/2020 10:12	84·05	AST	31	<30	U/l	no	NA
2·1	2	26/06/2020 12:40	-9·87	Triglycerides	0·61	0·8 - 2	mmol/l	no	NA
2·2	2	29/06/2020 10:39	-9·93	Leukocytes	3·2	4·0 – 11·0	*10^9/l	no	NA
2·2	2	29/06/2020 10:39	-9·93	Neutrophils	1·48	2·0 – 7·5	*10^9/l	no	NA
2·2	2	29/06/2020 10:39	-9·93	Monocytes	0·28	0·3 – 1·0	*10^9/l	no	NA
2·2	2	29/06/2020 10:39	-9·93	Triglycerides	0·49	0·8 - 2	mmol/l	no	NA
2·2	2	29/06/2020 10:39	-9·93	Glucose	3·8	4·0 - 5·6	mmol/l	no	NA
2·2	2	08/07/2020 09:03	-1·00	Leukocytes	3·7	4·0 – 11·0	*10^9/l	no	NA
2·2	2	08/07/2020 09:03	-1·00	Neutrophils	1·41	2·0 – 7·5	*10^9/l	no	NA
2·2	2	08/07/2020 09:03	-1·00	Monocytes	0·26	0·30 – 1·00	*10^9/l	no	NA

2·2	2	08/07/2020 09:03	-1·00	Urea	1·9	2·0 – 7·5	mmol/l	no	NA
2·2	2	10/07/2020 09:45	1·03	Monocytes	0·28	0·3 – 1·0	*10^9/l	no	NA
2·2	2	10/07/2020 09:45	1·03	Potassium	4·8	3·5 - 4·7	mmol/l	no	NA
2·2	2	10/07/2020 09:45	1·03	Urea	2·4	2·5 – 7·0	mmol/l	no	NA
2·2	2	11/07/2020 10:44	2·08	Monocytes	0·23	0·3 – 1·0	*10^9/l	no	NA
2·2	2	01/10/2020 09:20	84·02	MCHC	19·7	20·0-21·5	mmol/l	no	NA
2·3	2	10/02/2020 14:55	-153·78	Eosinophils	0·82	<0·50	*10^9/l	no	NA
2·3	2	10/02/2020 14:55	-153·78	Troponine T	24	<14·0	ng/l	no	NA
2·3	2	29/06/2020 11:30	-13·92	Eosinophils	0·6	<0·50	*10^9/l	no	NA
2·3	2	10/07/2020 10:25	-2·97	Eosinophils	0·66	<0·50	*10^9/l	no	NA
2·3	2	10/07/2020 10:25	-2·97	LDH	279	<250	U/l	no	NA
2·3	2	14/07/2020 09:05	0·98	Eosinophils	0·55	<0·50	*10^9/l	no	NA
2·3	2	03/08/2020 11:05	21·06	ALT	67	<45	U/l	yes	NA
2·3	2	03/08/2020 11:05	21·06	AST	90	<35	U/l	yes	NA
2·3	2	10/08/2020 11:18	28·07	Eosinophils	0·64	<0·50	*10^9/l	no	NA
2·3	2	10/08/2020 11:18	28·07	LDH	298	<250	U/l	no	Incorrectly elevated due to haemolysis
2·3	2	05/10/2020 16:58	84·30	MCHC	21·6	20·0 - 21·5	mmol/l	no	NA
2·3	2	05/10/2020 16:58	84·30	Eosinophils	0·54	<0·50	*10^9/l	no	NA
2·4	2	13/08/2020 10:16	28·03	Bilirubin total	17	<17	μmol/l	no	NA
2·5	2	01/07/2020 09:45	-15·07	Urea	2·3	2·5 – 7·0	mmol/l	no	NA
2·5	2	01/07/2020 09:45	-15·07	Triglycerides	0·67	0·8 - 2·0	mmol/l	no	NA
2·5	2	15/07/2020 09:17	-1·09	Urea	2·4	2·5 – 7·0	mmol/l	no	NA
2·5	2	17/07/2020 09:00	0·90	Urea	2	2·5 – 7·0	mmol/l	no	NA
2·5	2	18/07/2020 09:40	1·93	LDH	454	<250·0	U/l	yes	NA
2·5	2	18/07/2020 09:40	1·93	Urea	1·8	2·5 – 7·0	mmol/l	yes	NA
2·5	2	20/07/2020 08:42	3·89	Urea	1·6	2·5 – 7·0	mmol/l	no	NA
2·5	2	13/08/2020 10:20	27·95	Urea	2·2	2·5 – 7·0	mmol/l	no	NA
3·1	3	14/07/2020 15:30	-19·73	Triglycerides	0·76	0·8 - 2·0	mmol/l	no	NA

3·1	3	17/08/2020 09:03	14·00	Potassium	3·4	3·5 - 4·7	mmol/l	no	NA
3·2	3	05/08/2020 08:55	-1·02	Potassium	3·4	3·5 - 4·7	mmol/l	no	NA
3·2	3	05/08/2020 08:55	-1·02	Urea	1·6	2·5-7	mmol/l	no	NA
3·2	3	07/08/2020 09:42	1·02	Monocytes	0·24	0·30 – 1·00	*10^9/l	no	NA
3·2	3	08/08/2020 08:07	1·95	Potassium	3·4	3·5 - 4·7	mmol/l	no	NA
3·2	3	08/08/2020 08:07	1·95	Urea	2·1	2·5 – 7	mmol/l	no	NA
3·2	3	13/08/2020 10:45	7·06	Monocytes	0·19	0·30 – 1·00	*10^9/l	no	NA
3·2	3	20/08/2020 10:12	14·04	Monocytes	0·26	0·30 – 1·00	*10^9/l	no	NA
3·2	3	20/08/2020 10:12	14·04	Potassium	3·3	3·5 - 4·7	mmol/l	no	NA
3·2	3	27/08/2020 10:12	21·04	Monocytes	0·19	0·30 – 1·00	*10^9/l	no	NA
3·2	3	27/08/2020 10:12	21·04	Potassium	3·4	3·5-4·7	mmol/l	no	NA
3·2	3	27/08/2020 10:12	21·04	Urea	2	2·5 – 7	mmol/l	no	NA
3·2	3	03/09/2020 11:40	28·10	Potassium	3·4	3·5 - 4·7	mmol/l	no	NA
3·2	3	03/09/2020 11:40	28·10	Monocytes	0·2	0·30 – 1·00	*10^9/l	no	NA
3·2	3	29/10/2020 10:24	84·05	Potassium	3·4	3·5 - 4·7	mmol/l	no	NA
3·2	3	29/10/2020 10:24	84·05	Sodium	134	135 - 145	mmol/l	no	NA
3·3	3	31/07/2020 09:35	-10·00	Hemoglobin	7·8	8·4 - 10·8	mmol/l	no	NA
3·3	3	31/07/2020 09:35	-10·00	Hematocrit	0·38	0·41 - 0·53	l/l	no	NA
3·3	3	31/07/2020 09:35	-10·00	MCV	75	80 – 100	fl	no	NA
3·3	3	07/08/2020 13:54	-2·82	Hemoglobin	7·5	8·4 - 10·8	mmol/l	no	NA
3·3	3	07/08/2020 13:54	-2·82	Hematocrit	0·37	0·41 - 0·53	l/l	no	NA
3·3	3	07/08/2020 13:54	-2·82	MCV	76	80 – 100	fl	no	NA
3·3	3	11/08/2020 08:58	0·97	Hemglobin	7·7	8·4 - 10·8	mmol/l	no	volunteer donated blood 2 days before screening
3·3	3	11/08/2020 08:58	0·97	Hematocrit	0·38	0·41 - 0·53	l/l	no	volunteer donated blood 2 days before screening
3·3	3	11/08/2020 08:58	0·97	Leukocytes	3·9	4·0 – 11·0	*10^9/l	no	NA
3·3	3	11/08/2020 08:58	0·97	MCV	76	80 – 100	fl	no	volunteer donated blood 2

									days before screening
3·3	3	12/08/2020 09:50	2·01	Hemoglobin	8·1	8·4 -10·8	mmol/l	no	NA
3·3	3	12/08/2020 09:50	2·01	Hematocrit	0·4	0·41 - 0·53	l/l	no	NA
3·3	3	12/08/2020 09:50	2·01	MCV	76	80 – 100	fl	no	NA
3·3	3	17/08/2020 08:31	6·95	Hemoglobin	7·5	8·4 - 10·8	mmol/l	no	NA
3·3	3	17/08/2020 08:31	6·95	Hematocrit	0·38	0·41 - 0·53	l/l	no	NA
3·3	3	17/08/2020 08:31	6·95	Leukocytes	3·4	4·0 – 11·0	*10^9/l	no	NA
3·3	3	17/08/2020 08:31	6·95	MCV	77	80 – 100	fl	no	NA
3·3	3	17/08/2020 08:31	6·95	Neutrophils	1·74	2·00 – 7·50	*10^9/l	no	NA
3·3	3	24/08/2020 09:00	13·97	Hemoglobin	7·7	8·4 - 10·8	mmol/l	no	NA
3·3	3	24/08/2020 09:00	13·97	Hematocrit	0·4	0·41 - 0·53	l/l	no	NA
3·3	3	24/08/2020 09:00	13·97	Leukocytes	3·6	4·0 – 11·0	*10^9/l	no	NA
3·3	3	24/08/2020 09:00	13·97	MCV	78	80 – 100	fl	no	NA
3·3	3	24/08/2020 09:00	13·97	Neutrophils	1·99	2·00 – 7·50	*10^9/l	no	NA
3·3	3	31/08/2020 12:02	21·10	Hemoglobin	7·5	8·4 - 10·8	mmol/l	no	NA
3·3	3	31/08/2020 12:02	21·10	Hematocrit	0·38	0·41 - 0·53	l/l	no	NA
3·3	3	31/08/2020 12:02	21·10	MCV	77	80 – 100	fl	no	NA
3·3	3	07/09/2020 12:03	28·10	Hemoglobin	7·8	8·4 - 10·8	mmol/l	no	NA
3·3	3	07/09/2020 12:03	28·10	Hematocrit	0·38	0·41 - 0·53	l/l	no	NA
3·3	3	07/09/2020 12:03	28·10	MCV	76	80 – 100	fl	no	NA
3·3	3	31/10/2020 10:12	82·02	Hemoglobin	8·3	8·4 - 10·8	mmol/l	no	NA
3·3	3	31/10/2020 10:12	82·02	MCV	77	80 – 100	fl	no	NA
3·4	3	12/08/2020 08:47	-1·01	Creatinine	101	45 - 90	µmol/l	no	NA
3·4	3	12/08/2020 08:47	-1·01	LDH	263	<250	U/l	no	NA
3·4	3	14/08/2020 08:38	0·98	Creatinine	96	45 - 90	µmol/l	no	NA
3·4	3	15/08/2020 10:32	2·06	Creatinine	91	45 - 90	µmol/l	no	NA
3·4	3	19/08/2020 09:09	6·00	Creatinine	102	45 - 90	µmol/l	no	NA
3·4	3	03/09/2020 10:06	21·04	Creatinine	94	45 - 90	µmol/l	no	NA

3·4	3	10/09/2020 10:02	28·04	Creatinine	96	45 - 90	µmol/l	no	NA
3·5	3	10/08/2020 12:38	-2·93	Troponin-T	16	<14·0	ng/l	no	NA
3·5	3	12/08/2020 08:48	-1·09	Troponin-T	15	<14·0	ng/l	no	NA
3·5	3	27/08/2020 09:52	13·95	Urea	8·7	2·5 – 7·0	mmol/l	no	NA
4·1	4	08/07/2020 12:35	-49·86	Urea	1·9	2·5 – 7	mmol/l	no	NA
4·1	4	08/07/2020 12:35	-49·86	Alkaline phosphatase	119	<100	U/l	no	NA
4·1	4	26/08/2020 08:46	-1·02	Sodium	134	135-145	mmol/l	no	NA
4·1	4	26/08/2020 08:46	-1·02	Urea	2·2	2·5 – 7	mmol/l	no	NA
4·1	4	28/08/2020 08:37	0·98	Urea	2·3	2·5 – 7	mmol/l	no	NA
4·1	4	28/08/2020 08:37	0·98	Alkaline phosphatase	127	<100	U/l	no	NA
4·1	4	29/08/2020 10:20	2·05	Urea	2·1	2·5 – 7	mmol/l	no	NA
4·1	4	29/08/2020 10:20	2·05	Alkaline phosphatase	120	<100	U/l	no	NA
4·1	4	10/09/2020 10:50	14·07	Alkaline phosphatase	117	<100	U/l	no	NA
4·1	4	10/09/2020 10:50	14·07	Urea	1·7	2·5 – 7	mmol/l	no	NA
4·1	4	17/09/2020 10:39	21·06	Urea	2·1	2·5 – 7	mmol/l	no	NA
4·1	4	17/09/2020 10:39	21·06	Alkaline phosphatase	101	<100	U/l	no	NA
4·1	4	24/09/2020 12:05	28·12	Urea	1·2	2·5 – 7	mmol/l	no	NA
4·1	4	24/09/2020 12:05	28·12	Alkaline phosphatase	120	<100	U/l	no	NA
4·1	4	19/11/2020 10:35	84·06	Urea	1·8	2·5 – 7	mmol/l	no	NA
4·2	4	18/08/2020 11:31	-12·92	Hemoglobin	6·7	7·4 - 9·9	mmol/l	yes	NA
4·2	4	18/08/2020 11:31	-12·92	Hematocrit	0·34	0·36 - 0·46	mmol/l	yes	NA
4·2	4	18/08/2020 11:31	-12·92	Creatinine	91	45 - 90	mmol/l	yes	NA
4·2	4	24/08/2020 09:35	-7·00	Hemoglobin	7	7·4 - 9·9	mmol/l	no	NA
4·2	4	24/08/2020 09:35	-7·00	Macro thrombocytes present	N/A	N/A	N/A	no	NA
4·2	4	28/08/2020 10:44	-2·95	Hemoglobin	6·9	7·4 - 9·9	mmol/l	no	NA
4·2	4	28/08/2020 10:44	-2·95	Creatinine	93	45 - 90	mmol/l	no	NA
4·2	4	01/09/2020 09:30	1·00	Hemoglobin	6·7	7·4 - 9·9	mmol/l	no	NA
4·2	4	01/09/2020 09:30	1·00	Hematocrit	0·34	0·36 - 0·46	mmol/l	no	NA
4·2	4	01/09/2020 09:30	1·00	Erytrocytes	3·91	4·0 – 5·20	*10^12/l	no	NA

4·2	4	01/09/2020 09:30	1·00	Creatinine	92	45 - 90	mmol/l	no	NA
4·2	4	02/09/2020 10:04	2·02	Hemoglobin	6·7	7·4 - 9·9	mmol/l	no	NA
4·2	4	02/09/2020 10:04	2·02	Hematocrit	0·35	0·36 - 0·46	mmol/l	no	NA
4·2	4	02/09/2020 10:04	2·02	Erytrocytes	3·97	4·00 – 5·20	*10^12/l	no	NA
4·2	4	02/09/2020 10:04	2·02	Creatinine	95	45 - 90	mmol/l	no	NA
4·2	4	08/09/2020 13:05	8·14	Hemoglobin	6·7	7·4 - 9·9	mmol/l	no	NA
4·2	4	08/09/2020 13:05	8·14	Hematocrit	0·34	0·36 - 0·46	mmol/l	no	NA
4·2	4	08/09/2020 13:05	8·14	Erythrocytes	3·96	4·0 – 5·20	*10^12/l	no	NA
4·2	4	15/09/2020 12:45	15·13	Hemoglobin	6·6	7·4 - 9·9	mmol/l	no	NA
4·2	4	15/09/2020 12:45	15·13	Hematocrit	0·34	0·36 - 0·46	mmol/l	no	NA
4·2	4	15/09/2020 12:45	15·13	Erythrocytes	3·94	4·00 – 5·20	*10^12/l	no	NA
4·2	4	15/09/2020 12:45	15·13	Monocytes	0·25	0·30 – 1·00	*10^9/l	no	NA
4·2	4	21/09/2020 17:04	21·31	Hemoglobin	6·4	7·4 - 9·9	mmol/l	no	NA
4·2	4	21/09/2020 17:04	21·31	Hematocrit	0·34	0·36 - 0·46	mmol/l	no	NA
4·2	4	21/09/2020 17:04	21·31	Erythrocytes	3·92	4·0 – 5·20	*10^12/l	no	NA
4·2	4	21/09/2020 17:04	21·31	Potassium	3·5	3·5 - 4·7	mmol/l	no	NA
4·2	4	28/09/2020 09:15	27·98	Hemoglobin	6·3	7·4 - 9·9	mmol/l	no	NA
4·2	4	28/09/2020 09:15	27·98	Hematocrit	0·33	0·36 - 0·46	mmol/l	no	NA
4·2	4	28/09/2020 09:15	27·98	Erythrocytes	3·79	4·0 – 5·20	*10^12/l	no	NA
4·2	4	28/09/2020 09:15	27·98	Young lymfocytes present	N/A	N/A	N/A	no	NA
4·2	4	28/09/2020 09:15	27·98	Hypochromasia	N/A	N/A	N/A	no	NA
4·2	4	24/11/2020 09:50	85·01	Hemoglobin	6·6	7·4 - 9·9	mmol/l	no	NA
4·2	4	24/11/2020 09:50	85·01	Hematocrit	0·34	0·36 - 0·46	mmol/l	no	NA
4·2	4	24/11/2020 09:50	85·01	Erythrocytes	3·89	4·00 – 5·20	*10^12/l	no	NA
4·2	4	24/11/2020 09:50	85·01	MCHC	19·5	20 - 21·5	mmol/l	no	NA
4·2	4	24/11/2020 09:50	85·01	Creatinine	91	45 - 90	mmol/l	no	NA
4·2	4	24/11/2020 09:50	85·01	Microplanie present	N/A	N/A	N/A	no	NA
4·3	4	27/07/2020 09:54	-37·97	Abnormal lymphocytes suspect reactively present	N/A	N/A	N/A	no	NA

4·3	4	02/09/2020 08:48	-1·02	Lymfocytes	3·75	1·0 – 3·5	*10^9/l	no	NA
4·3	4	02/09/2020 08:48	-1·02	AST	50	<30	U/l	no	NA
4·3	4	04/09/2020 11:22	1·09	Macro thrombocytes present	NA	NA	NA	no	NA
4·3	4	04/09/2020 11:22	1·09	AST	36	<30	U/l	no	NA
4·3	4	24/11/2020 09:37	82·01	Abnormal lymphocytes suspect reactively present	N/A	N/A	N/A	no	NA
4·4	4	09/09/2020 09:15	1·98	Monocytes	0·26	0·30 – 1·00	*10^9/l	no	NA
4·4	4	21/09/2020 17:46	14·34	Hemoglobin	7·3	7·4 - 9·9	mmol/l	no	NA
4·4	4	21/09/2020 17:46	14·34	Hematocrit	0·36	0·36 - 0·46	mmol/l	no	NA
4·4	4	21/09/2020 17:46	14·34	Eosinophils	0·65	<0·50	*10^9/l	no	NA
4·4	4	21/09/2020 17:46	14·34	Potassium	3·4	3·5 - 4·7	mmol/l	no	NA
4·4	4	28/09/2020 18:01	21·35	Lymphocytes	3·91	1·0 - 3·5	*10^9/l	no	NA
4·4	4	05/10/2020 17:35	28·33	Lymphocytes	4·06	1·0 - 3·5	*10^9/l	no	NA
4·5	4	28/09/2020 17:54	21·13	Erythrocytes	4·48	4·0 – 5·20	*10^12/l	no	NA
4·5	4	28/09/2020 17:54	21·13	Monocytes	1·03	0·30 – 1·00	*10^9/l	no	NA
5·1	5	25/08/2020 10:30	-96·95	Potassium	4·8	3·5 - 4·7	mmol/l	no	NA
5·1	5	01/12/2020 09:34	1·02	Thrombocytes	147	150 - 400	*10^9/l	no	NA
5·1	5	07/12/2020 10:05	7·04	Erythrocytes	4·46	4·00 – 5·20	*10^12/l	no	NA
5·1	5	28/12/2020 10:40	28·06	Thrombocytes	112	150 - 400	*10^9/l	yes	NA
5·1	5	13/01/2021 12:20	44·13	RCD	11·6	12 - 13·6	%	no	NA
5·1	5	22/02/2021 10:42	84·06	RCD	11·8	12 - 13·6	%	no	NA
5·2	5	01/12/2020 14:43	-1·78	Urea	7·1	2·5 – 7	mmol/l	no	NA
5·2	5	04/12/2020 10:20	1·04	Erythrocytes	4·49	4·00 – 5·20	*10^12/l	no	NA
5·2	5	04/12/2020 10:20	1·04	Bilirubin total	22	<17	µmol/l	no	NA
5·2	5	10/12/2020 09:40	7·01	Hematocrit	0·41	0·41 - 0·53	l/l	no	NA
5·2	5	10/12/2020 09:40	7·01	Erythrocytes	4·43	4·00 – 5·20	*10^12/l	no	NA
5·2	5	10/12/2020 09:40	7·01	RCD	13·9	12 - 13·6	%	no	NA
5·2	5	17/12/2020 10:41	14·05	Erythrocytes	4·48	4·0 – 5·2	*10^12/l	no	NA
5·2	5	23/12/2020 11:03	20·07	Eryhrocytes	4·46	4·0 – 5·20	*10^12/l	no	NA

5·2	5	29/12/2020 10:28	26·05	AST	37	<35	U/l	no	NA
5·2	5	29/12/2020 10:28	26·05	Bilirubin total	17	<17	µmol/l	no	NA
5·3	5	01/12/2020 13:15	-6·83	Leukocytes	3·4	4·0 – 11·0	*10^9/l	no	NA
5·3	5	01/12/2020 13:15	-6·83	Leukocytes	3·4	4·0 – 11·0	*10^9/l	no	NA
5·3	5	07/12/2020 09:45	-0·98	Leukocytes	3·7	4·0 – 11·0	*10^9/l	no	NA
5·3	5	07/12/2020 09:45	-0·98	Neutrophils	1·94	2·0 – 7·5	*10^9/l	no	NA
5·3	5	09/12/2020 09:35	1·02	Leukocytes	3·1	4·0 – 11·0	*10^9/l	no	NA
5·3	5	09/12/2020 09:35	1·02	RCD	14·1	12 - 13·6	%	no	NA
5·3	5	09/12/2020 09:35	1·02	Neutrophils	1·47	2·0 – 7·5	*10^9/l	no	NA
5·3	5	09/12/2020 09:35	1·02	LDH	<268	<250	U/l	no	Incorrectly elevated due to haemolysis
5·3	5	10/12/2020 09:53	2·03	Leukocytes	3·4	4·0 – 11·0	*10^9/l	no	NA
5·3	5	10/12/2020 09:53	2·03	RCD	14·1	12 - 13·6	%	no	NA
5·3	5	10/12/2020 09:53	2·03	Neutrophils	1·78	2·0 – 7·5	*10^9/l	no	NA
5·3	5	15/12/2020 09:42	7·02	Leukocytes	2·9	4·0 – 11·0	*10^9/l	no	NA
5·3	5	15/12/2020 09:42	7·02	RCD	13·7	12 - 13·6	%	no	NA
5·3	5	15/12/2020 09:42	7·02	Neutrophils	1·55	2·0 – 7·5	*10^9/l	no	NA
5·3	5	15/12/2020 09:42	7·02	Monocytes	0·22	0·30 – 1·00	*10^9/l	no	NA
5·3	5	22/12/2020 09:09	14·00	leukocytes	3·4	4·0 – 11·0	*10^9/l	no	NA
5·3	5	22/12/2020 09:09	14·00	Neutrophils	1·68	2·0 – 7·5	*10^9/l	no	NA
5·3	5	29/12/2020 09:55	21·03	Hematocrit	0·4	0·41 - 0·53	l/l	no	NA
5·3	5	29/12/2020 09:55	21·03	Leukocytes	3·2	4·0 – 11·0	*10^9/l	no	NA
5·3	5	29/12/2020 09:55	21·03	Neutrophils	1·66	2·0 – 7·5	*10^9/l	no	NA
5·3	5	05/01/2021 10:42	28·06	Leukocytes	3·1	4·0 – 11·0	*10^9/l	no	NA
5·3	5	05/01/2021 10:42	28·06	Neutrophils	1·65	2·0 – 7·5	*10^9/l	no	NA
5·3	5	02/03/2021 13:13	84·17	Leukocytes	3·8	4·0 – 11·0	*10^9/l	no	NA
5·4	5	08/12/2020 11:40	-1·92	MCHC	19·6	20·0 - 21·5	mmol/l	no	NA
5·4	5	11/12/2020 09:05	0·98	MCHC	19·5	20·0 - 21·5	mmol/l	no	NA
5·4	5	23/12/2020 13:11	13·15	MCHC	19·6	20·0 - 21·5	mmol/l	no	NA

5·4	5	29/12/2020 14:06	19·18	RCD	11·9	12·0 - 13·6	%	no	NA
5·5	5	08/12/2020 10:43	-2·00	Triglycerides	0·69	0·8 - 2	mmol/l	no	NA
5·5	5	11/12/2020 09:15	0·94	MCHC	19·8	20·0 - 21·5	mmol/l	no	NA
5·5	5	17/12/2020 10:29	6·99	RCD	11·9	12·0 - 13·6	%	no	NA
5·5	5	23/12/2020 10:53	13·01	AST	35	<30	U/l	no	NA
5·5	5	29/12/2020 10:52	19·01	Hemoglobin	7·1	7·4 - 9·9	mmol/l	no	NA
5·5	5	29/12/2020 10:52	19·01	Hematocrit	0·35	0·36 - 0·46	mmol/l	no	NA
5·5	5	29/12/2020 10:52	19·01	ALT	43	<35	U/l	no	NA
5·5	5	29/12/2020 10:52	19·01	AST	59	<30	U/l	no	NA
5·5	5	29/12/2020 10:52	19·01	Troponin-T	16	<14·0	ng/l	no	NA
5·5	5	07/01/2021 13:12	28·11	ALT	46	<35	U/l	yes	NA
5·5	5	07/01/2021 13:12	28·11	AST	82	<30	U/l	yes	NA
5·5	5	04/03/2021 13:54	84·13	MCHC	19·8	20·0 - 21·5	mmol/l	no	NA
5·5	5	04/03/2021 13:54	84·13	ALT	43	<35	U/l	no	NA
5·5	5	04/03/2021 13:54	84·13	AST	43	<30	U/l	no	NA

AST: aspartate aminotransferase; LDH: lactate dehydrogenase; MCHC: mean corpuscular hemoglobin concentration; MCV: mean corpuscular volume; ALT: alanine aminotransferase; RCD: Red blood cell deformability.

Appendix table 11 – TB31F pharmacokinetic parameters

Route	Group	Dose Group	C_{max} (µg/mL)	T_{max} (days)	$AUC_{0\text{-}12\ weeks}$ (µg·h/mL)	$AUC_{0\text{-}Inf}$ (µg·h/mL)	$T^{1/2,\ elim}$ (days)	F	CL (L/h)	V_d (L)
iv	1	0·1 mg/kg	2·94 (2·34-3·69)	EOI	1507 (1180-1924)	1926 (1194-3106)	38·8 (22·6-66·6)	NA	0·0019 (0·0010-0·0036)	2·49 (2·06-3·01)
iv	2	1 mg/kg	28·3 (14·4-55·5)	EOI	9913 (7628-12883)	11606 (8478-15887)	32·0 (21·5-47·6)	NA	0·0024 (0·0021-0·0028)	2·65 (1·67-4·21)
iv	3	3 mg/kg	80·8 (55·2-118)	EOI	36260 (30519-43081)	40926 (32051-52258)	27·2 (19·9-37·2)	NA	0·0028 (0·0013-0·0060)	2·61 (1·21-5·64)
iv	4	10 mg/kg	255 (177-367)	EOI	109283 (71074-168034)	125417 (81567-192841)	28·9 (23·9-35·0)	NA	0·0028 (0·0017-0·0047)	2·78 (1·50-5·14)
iv	1-4	0·1-10 mg/kg	NA	EOI	NA	NA	31·1 (19·9-48·6)	NA	0·0024 (0·0013-0·0044)	2·63 (1·58-4·38)
sc	5	100 mg	9·28 (3·49-24·7)	4·3 (1·15-16·0)	8040 (2815-22963)	10104 (3262-31298)	36·1 (22·0-59·1)	0·52 (0·17-1·61)	NA	NA
iv and sc	1-5	0·1-10 mg/kg and 100 mg	NA	EOI	NA	NA	32·2 (20·3-51·1)	NA	NA	NA

Pharmacokinetic parameters derived from noncompartmental analysis reported as geometric mean (95% confidence interval). For group 1, serum TB31F concentrations were unavailable for 1 participant at day 28 and all 5 participants at day 56. One participant in group 5 had no detectable or very low concentrations of TB31F at any time point and was not included in this analysis. C_{max} = maximum concentration. T_{max} = time to C_{max}. $AUC_{0\text{-}12\ weeks}$ = area under the concentration-time curve from 0 to 12 weeks. $AUC_{0\text{-}Inf}$ = area under the concentration-time curve from time zero to infinity. $T^{1/2,\ elim}$ = elimination half-life. F = bioavailability. CL = clearance. V_d= apparent volume of distribution. iv = intravenous. EOI = end of infusion. NA = not applicable. sc = subcutaneous.

CLINICAL TRIAL PROTOCOL

Phase 1 study to evaluate the safety, tolerability, and pharmacokinetics and pharmacodynamics of monoclonal antibody TB31F in healthy malaria-naive adults in the Netherlands

Version 1.6

Date: 5 October 2020

Confidentiality Statement

This document is confidential and is to be distributed for review only to investigators, potential investigators, consultants, study staff, and applicable Independent Ethics Committees or Institutional Review Boards. The contents of this document shall not be disclosed to others without written authorization from CVIA (or others, as applicable).

PROTOCOL TITLE: Phase 1 study to evaluate the safety, tolerability, and pharmacokinetics and pharmacodynamics of monoclonal antibody TB31F in healthy malaria-naive adults in the Netherlands

**Protocol ID**	NL69779.091.19
**Short title**	Safety, tolerability, and pharmacokinetics and pharmacodynamics of TB31F
**EudraCT number**	2019-001904-39
**Clinicaltrials.gov Identifier**	NCT04238689
**Version**	1.6
**Date**	5 October 2020
**Protocol Author**	Christian F. Ockenhouse MD PhD<br>Saskia C. van der Boor MD<br>Robert W. Sauerwein MD PhD
**Principal investigator(s)**	**Matthew McCall MD PhD**<br>Radboudumc, Department of Medical Microbiology<br>Tel: +31 (0)24 3615363<br>Email: matthew.mccall@radboudumc.nl
**Senior Co-Investigator**	**Robert Sauerwein MD PhD**<br>Department of Medical Microbiology<br>Tel : +31 (0)24 3610577<br>Fax :+31 (0)24 3614666<br>Email: Robert.Sauerwein@radboudumc.nl
**Clinical Investigator(s)**	**Saskia C. van der Boor MD**<br>Radboudumc, Department of Medical Microbiology<br>Tel: +31 (0)24 3619515<br>Fax: +31 (0)24 3614666<br>Email: saskia.vanderboor@radboudumc.nl<br><br>**Merel J. Smit MD**<br>Radboudumc, Department of Medical Microbiology<br>Tel: +31 (0)24 3619515<br>Fax: +31 (0)24 3614666<br>Email: merel.smit@radboudumc.nl
**Sponsor**	Stichting Katholieke Universiteit Nijmegen<br>Department of Medical Microbiology<br>Geert Grooteplein-Zuid 10<br>6525 GA, Nijmegen, The Netherlands
**Funder/Subsidizing party**	PATH MVI<br>2201 Westlake Avenue, Suite 200 Seattle, Washington

	98121, USA
**Independent expert (s)**	**Arjan van Laarhoven MD PhD**<br>Radboudumc Department of Internal Medicine<br>Tel: +31 24 361 69 80.<br>Email: Arjan.vanLaarhoven@radboudumc.nl
**Laboratory sites**	**Eric Aaldring**<br>Radboudumc Clinical Chemical Laboratory<br>Tel: +31 (0)24 3616997<br>Fax: +31 (0)24 3568408<br>e-mail: Eric.Aaldring@radboudumc.nl<br><br>**Heiman Wertheim MD PhD**<br>Clinical Microbiology Laboratory<br>Tel: +31 (0)24 36 14281<br>email: Heiman.Wertheim@radboudumc.nl
**Pharmacy**	**Rob ter Heine PharmD PhD**<br>Radboudumc Clinical Pharmacy - Clinical Trial Unit<br>Tel: +31 (0)24 3616405<br>Fax: +31 (0)24 3668755<br>email: R.terHeine@radboudumc.nl<br><br>**Elin M. Svensson PhD**<br>Radboudumc Clinical Pharmacy - Clinical Trial Unit<br>Tel: +31 (0)24 3616405<br>Fax: +31 (0)24 3668755<br>email: Elin.Svensson@radboudumc.nl
**Pharmacokinetic Assays**	Imperial College London, Human Immunology Laboratory<br>IAVI Core Lab<br>2nd Floor, Lift Bank D<br>Chelsea and Westminster Hospital 369<br>Fulham Road, London, UK SW10 9NH

**Standard Membrane Feeding Assays**	**Prof. Teun Bousema**<br>**Matthijs Jore, PhD**<br>RadboudUMC<br>Department of Medical Microbiology<br>Geert Grooteplein-Zuid 10<br>6525 GA, Nijmegen, The Netherlands<br>And<br>Laboratory Malaria and Vector Research, NIH<br>Bethesda MD
**CRO**	**Radboud Technology Center Clinical Studies**<br>Geert Grooteplein-Zuid 10<br>6525 GA, Nijmegen, The Netherlands

PROTOCOL SIGNATURE SHEET

The signature below constitutes approval of this protocol and the attachments and provides required assurances that this trial will be conducted according to all stipulations of the protocol, including all statements regarding confidentiality, and according to local legal and regulatory requirements, applicable to ICH E6 [R2] guidelines.

**Name**	**Signature**	**Date**
**Principal Investigator:**<br>Matthew McCall MD PhD<br>Department of Medical Microbiology		28 October 2020

TABLE OF CONTENTS

TABLE OF CONTENTS – Figures and Tables 9
LIST OF ABBREVIATIONS AND RELEVANT DEFINITIONS 10
INTRODUCTION AND RATIONALE 14
1.1 Introduction 14
1.2 Rationale 16
2. HYPOTHESIS, OBJECTIVES, AND ENDPOINTS 17
2.2 Study Objectives 17
2.3 Study Endpoints 17
3. STUDY DESIGN 18
3.1 Visit intervals 20
4. STUDY POPULATION 21
4.2 Inclusion criteria 21
4.3 Exclusion criteria 22
4.4 Sample size calculation 24
5. TREATMENT OF SUBJECTS 24
5.2 Use of co-intervention (if applicable) 24
5.3 Medication available for adverse effects (if applicable) 25
6. INVESTIGATIONAL PRODUCT 25
6.2 Summary of findings from non-clinical studies 26
6.3 Summary of findings from clinical studies 29
6.4 Summary of known and potential risks and benefits 29
6.4.1 Risk Assessment 29
6.4.2 Benefit assessment 35
6.4.3 Overall risk/benefit conclusion 35
6.5 Description and justification of dosages proposed in the clinical trial 35
6.6 Dosages, dosage modifications and method of administration 37
6.7.1 Presentation and formulation 40
6.7.2 Stability and storage 40
6.7.3 Dose preparation and administration 40
6.7.4 Labeling 41
6.8 Drug accountability 41
7. NON-INVESTIGATIONAL PRODUCT 41
8. METHODS 41
8.1.1 Primary study parameters/endpoints 41
8.1.2 Secondary study parameters/endpoints 42
8.1.3 Exploratory study parameters/endpoints 42
8.2 Randomization, blinding and treatment allocation 42
8.3 Study schedule and procedures 42
8.3.1 Recruitment of subjects 43
8.3.2 Screening visit (visit 1) 43

8.3.3 Inclusion visit (visit 2) 44
8.3.4 mAb TB31F inoculation visit (visit 3) 45
8.3.5 Follow-up after mAb TB31F administration (visits 4-12) 46
8.3.6 Unscheduled visits 46
8.3.7 Medical history 47
8.3.8 Physical Examination 47
8.3.9 Height and Weight 47
8.3.10 Vital Signs 47
8.3.11 Laboratory Evaluations 48
8.3.12 Urine Toxicology analysis 49
8.3.13 Pregnancy Test 49
8.3.14 Discontinuation of study procedures 49
8.3.15 Standard Membrane Feeding Assay 49
8.3.16 Pharmacokinetic laboratory assay 49
8.3.17 Safety Assessments 49
8.3.18 Time and Event procedures: TB31F 44
8.4 Withdrawal of individual subjects 46
8.4.1 Handling of withdrawals 46
8.4.2 Withdrawals at specific time-points 47
8.5 Replacement of individual subjects after withdrawal 48
8.6 Follow-up of subjects withdrawn from treatment 48
8.7 Premature termination of the study 48
9. SAFETY ASSESSMENT AND REPORTING 49
9.2 AEs, SAEs and SUSARs 49
9.2.1 Adverse events (AEs) 49
9.2.2 Clinical Laboratory Parameters 49
9.2.3 Serious adverse events (SAEs) 49
9.2.4 Suspected unexpected serious adverse reactions (SUSARs) 50
9.3 Annual safety report 51
9.4 Follow-up of adverse events 51
9.4.1 Adverse event data collection 51
9.4.2 Monitoring periods for safety events 52
9.4.3 Recording of adverse event data collection 52
9.4.4 Assessment of causality 53
9.4.5 Follow-up of adverse events 54
9.5 Safety Monitoring Committee 54
9.5.1 Local safety monitor 54
9.5.2 Safety monitoring committee (SMC) 54
9.5.3 Review of safety data by the safety monitor and SMC 55
10. STATISTICAL ANALYSIS 57
10.1 Primary study parameter(s) 58
10.2 Secondary study parameter(s) 59
10.3 Other study parameters 59
10.4 Interim analysis 60

10.5 Accounting for Missing, Unused, and Spurious Data 60
11. ETHICAL CONSIDERATIONS 60
11.2 Recruitment and consent 60
11.3 Benefits and risks assessment, group relatedness 61
11.4 Ethical aspects concerning the use of human subjects 61
11.5 Compensation for injury 61
11.6 Compensation 62
12. ADMINISTRATIVE ASPECTS, MONITORING AND PUBLICATION 63
12.1.1 Source data 63
12.1.2 Confidentiality 64
12.2 Monitoring and Quality Assurance 64
12.3 Amendments 66
12.4 Annual progress report 66
12.5 Temporary halt and (prematurely) end of study report 66
12.6 Public disclosure and publication policy 67
13. STRUCTURED RISK ANALYSIS 67
13.2 Update of risk assessment for amendment v1.6 (prior to group 5) 69
13.3 Synthesis 71
14. REFERENCES 72
15. Appendix 1 – Classification of Adverse Events 74
Vital Signs 76
Laboratory abnormalities 77

TABLE OF CONTENTS – Figures and Tables

Figure 1 Asexual blood stage parasites and sexual stage gametocytes from *P. falciparum*

Figure 2 Testing the efficacy of mAb TB31F by the Standard Membrane Feeding Assay (SMFA).

Figure 3 Staggered dosing trial design for monoclonal antibody TB31F administration

Figure 4 Lot number and label in Dutch and English

Figure 5 Inhibitory activity of native, chimeric and humanized form of 85RF45.1 antibody tested in SMFA.

Figure 6 Transmission Reducing Activity of Catalent monoclonal antibodies measured at 1.1µg/ml, 3.3µg/ml and 10µg/ml.

Figure 7 Serum TB31F time course for 18.75 and 24.8mg/kg IV dose

Figure 8 Pharmacokinetic properties of monoclonal antibodies

Figure 9 TB31F vial labels in Dutch and English

Table 1 Study groups
Table 2 Group intervals for Visit 2 (TB31F administration)
Table 3 Permissible study visit intervals
Table 4 Dosing groups of TB31F Administration in Sprague Dawley Rats
Table 5 Risk Assessment
Table 6 TB31F formulation

Appendix

Table A1 Grading of local solicited adverse events following mAb TB31F Administration
Table A2 Grading of systemic solicited adverse events following mAb TB31F Administration
Table A3 Vital signs
Table A4 Laboratory abnormalities

LIST OF ABBREVIATIONS AND RELEVANT DEFINITIONS

**ABR**	ABR form, General Assessment and Registration form, is the application form that is required for submission to the accredited Ethics Committee (In Dutch, ABR = Algemene Beoordeling en Registratie)
**AB**	Antibody
**AE**	Adverse Event
**AR**	Adverse Reaction
**AUC**	Area under the serum concentration-time curve
**AUC0-t**	AUC up to the last measurable concentration
**AUC0-τ**	AUC to the end of the dosing period
**BP**	Blood pressure
**CA**	Competent Authority
**CBC**	Complete Blood Count
**CCMO**	Central Committee on Research Involving Human Subjects; in Dutch: Centrale Commissie Mensgebonden Onderzoek
**Cmax**	Maximum observed serum concentration
**CV**	Curriculum Vitae
**DLT**	Dose Limiting Toxicities
**DSMB**	Data Safety Monitoring Board
**EOI**	End Of Infusion
**EU**	European Union
**EudraCT**	European drug regulatory affairs Clinical Trials
**GCP**	Good Clinical Practice
**GP**	General Practitioner
**IB**	Investigator’s Brochure
**IC**	Informed Consent
**IV**	Intravenous(ly)
**IMP**	Investigational Medicinal Product
**IMPD**	Investigational Medicinal Product Dossier
**MC**	Medium Care
**METC**	Medical research ethics committee (MREC); in Dutch: medisch ethische toetsing commissie (METC)
**PBMC**	Peripheral Blood Mononuclear Cells
**pK**	pharmacokinetic (pK)

**Racc**	accumulation index
**RUMC**	Radboud University Medical Center
**(S)AE**	(Serious) Adverse Event
**SMFA**	Standard Membrane Feeding Assay
**SC**	Subcutaneous(ly)
**SPC**	Summary of Product Characteristics (in Dutch: officiële productinfomatie IB1-tekst)
**Sponsor**	The sponsor is the party that commissions the organisation or performance of the research, for example a pharmaceutical company, academic hospital, scientific organisation or investigator. A party that provides funding for a study but does not commission it is not regarded as the sponsor, but referred to as a subsidising party.
**SUSAR**	Suspected Unexpected Serious Adverse Reaction
**T1/2**	Terminal serum half-life
**TBV**	Transmission Blocking Vaccine
**Tmax**	Time to reach maximum serum concentration
**WMO**	Medical Research Involving Human Subjects Act (in Dutch: Wet Medisch-wetenschappelijk Onderzoek met Mensen

SUMMARY

Rationale: Malaria, a disease caused by the parasite *Plasmodium*, is one of the world's major infectious diseases. The availability of a monoclonal antibody to block transmission when used in combination with other malaria control measures would be a critical and decisive step on the path to malaria elimination.

Objectives: The primary objective is to assess the safety and tolerability of monoclonal antibody TB31F administered intravenously (IV) at escalating dose levels and subcutaneously (SC) in healthy, malaria naive, adults in the Netherlands. The secondary objective is to evaluate the serum pharmacokinetics of mAb TB31F at each dose level by ELISA, and the functional transmission blocking activity of serum in SMFA.

Hypothesis: Monoclonal antibody TB31F will be safe and well tolerated in healthy adults.

Study design: TB31F is a first-in-human phase I, open-label, single-site, dose-escalation study to determine the safety, tolerability and pharmacokinetics and pharmacokinetics of the mAb TB31F. MAb TB31F will be administered intravenously or subcutaneously as a single dose to healthy adults.

Study population: 25 healthy, malaria naive, adults (males and females) aged 18 – 35 years.

Intervention: Group 1 (n=5) will receive 0.1 mg/kg, group 2 (n=5) will receive 1 mg/kg, group 3 (n=5) will receive 3 mg/kg, and group 4 (n=5) will receive 10 mg/kg mAb TB31F by intravenous infusion. Group 5 (n=5) will receive 100 mg subcutaneously. Safety follow-up and collection of serum/plasma for pharmacokinetic and pharmacodynamic measurements will be done at following time points: baseline, end of infusion (EOI), 1, 3, 6 and 24 hours and 2, 7, 14, 21, 28, 56 and 84 days after administration. Extra follow-up visits at 4 and 10 days after administration for collection of serum/plasma for pharmacokinetic and pharmacodynamic measurements will be performed in group 5.

Main study parameters/endpoints: The primary study endpoint will be determined by the occurrence of solicited AEs, as defined by the systemic presence of fever, headache, myalgia, fatigue, chills, and rash, and local AEs, defined as pain and /or redness and swelling at the injection site. Unsolicited local and general (serious) adverse events will be recorded as described by the FDA Toxicity Grading Scale for Healthy Adult and Adolescent Subjects Enrolled in Preventative Vaccine Clinical Trials and adapted to the Radboud University Medical Center laboratory values (see Appendix A4 for list of laboratory values to be monitored). The pharmacokinetic endpoint will be determined by serum concentration of TB31F at each dose level assessed by ELISA and the pharmacodynamic endpoint will be assessed by SMFA (figure 2).

Nature and extent of the burden and risks associated with participation, benefit and group relatedness: There are no benefits to participating in this study. Subjects are not protected against malaria after participating. TB31F has not been administered in humans before, therefore we do not know what side effects TB31F can give. Participating in this trial includes the burden of mAb TB31F administration (intravenously or subcutaneous), premedication, placement of intravenous infusion catheter(s), multiple blood sampling tests, frequent follow-up visits, physical examinations, screening for HIV, Hepatitis B and Hepatitis C, a pregnancy test (for females), presumptive and/or symptom-

driven COVID-19 diagnostics (where required by current Radboudumc-wide or Medium Care [MC]-specific guidelines), filling out a memory aid and abiding to all study rules.

INTRODUCTION AND RATIONALE

1.1 Introduction

Malaria is one of the most devastating infectious diseases worldwide. It is a public health problem in over 90 countries worldwide for over 2 billion people. In 2016, there were 216 million cases of malaria and 445,000 deaths, of which 91% took place in Africa [1]. Human malaria is caused by protozoa of the genus *Plasmodium*, including *P. falciparum, P. vivax, P. ovale, P. malariae* and *P. knowlesi*. Parasites in the sporozoites stage are injected into the skin by an infected female *Anopheles* mosquito, which penetrate skin capillaries. Sporozoites are subsequently transported to the liver, where they develop and multiply in hepatocytes before release into the blood (merozoite stage), invading red blood cells for further maturation and multiplication. The cyclical proliferation of asexual stages within the human red blood cells is responsible for the occurrence of clinical symptoms. Malaria mortality is primarily due to organ dysfunction, including the brain, following sequestration of infected erythrocytes in the microvasculature.

Young children with poor access to health services are particularly at risk [1]. Other high-risk groups include women during pregnancy, non-immune travelers, refugees, displaced persons, and labor forces entering endemic areas. The disease additionally forms a profound economic burden for the affected countries, which are already struggling with poverty [1]. The urgency of the situation is emphasized by the waning effectiveness of all currently registered anti-malarials due to the fast emergence and spread of resistance and the absence of an effective vaccine [2]. The World Health organization (WHO) has declared malaria control a global development priority, and has modified their recommendation to include both malaria control programs to eradication programs [3]. However, it is widely accepted that malaria eradication is unlikely attainable with the currently available tools [4, 5]. Furthermore, the epidemiology of malaria has been changing over recent years due to a combination of factors including increasing resistance of the malaria parasite to chemotherapy, increasing insecticide resistance of the Anopheles mosquito vectors, ecological and climate changes and increased international travel to malaria-endemic areas [1].

A major challenge for malaria elimination is the highly efficient spreading of malaria parasites. During the malaria life cycle, the formation of male and female gametocytes is essential for parasite transmission to the female *Anopheles* mosquito vector when blood that contains gametocytes is ingested by the mosquitoes.

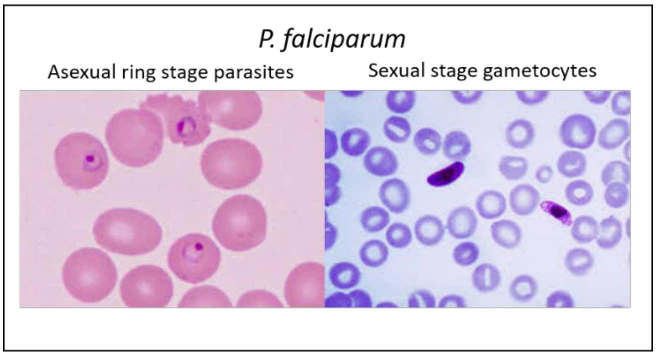

Figure 1 – Asexual blood stage parasites and sexual stage gametocytes from *P. falciparum*

Circulating gametocytes do not cause clinical pathology or symptoms but play an essential role in the onward transmission of malaria infections. A transmission blocking vaccine (TBV) aims to prevent the sexual development of parasites in the mosquito's midgut. Gametocytes of *P. falciparum* synthesize Pfs48/45, an important antigen candidate for transmission blocking vaccines expressed by gametocytes and early gametes. Male gametocytes lacking Pfs48/45 are unable to bind to female gametocytes in the mosquito gut. Antibodies (Abs) are ingested during the blood meal and can bind sexual forms in the mosquito gut thereby preventing oocyst development. Previously, a rat monoclonal antibody (85RF45.1) has been developed that reacts in an indirect immunofluorescent assay with Pfs45/48 and interrupts transmission of P. *falciparum* in the Standard Membrane Feeding Assay (SMFA) (Figure 2).

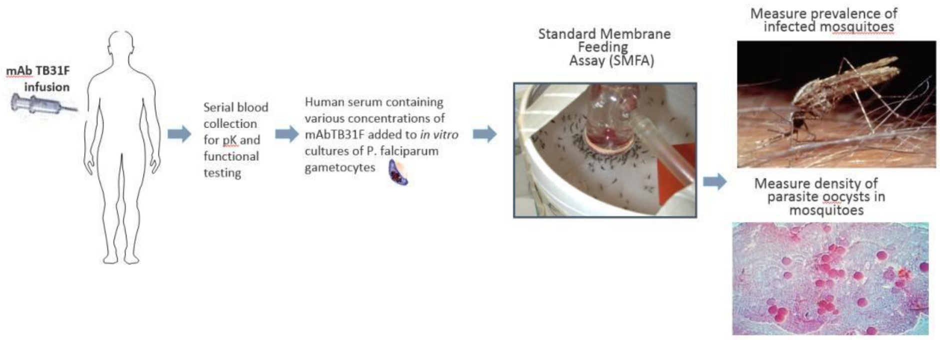

Figure 2 – Testing the efficacy of mAb TB31F by the Standard Membrane Feeding Assay (SMFA).
MAb TB31F is given at different concentrations to study volunteers. Serum is collected at different time points throughout the study. A SMFA is performed and parasite oocyst density is calculated.

SMFA is the most widely used assay to determine the transmission-reducing activity (TRA) of a monoclonal antibody in serum antibody functionality [6]. Laboratory reared anopheles mosquitoes are fed through membrane feeders with in vitro cultured gametocytes to which serum or purified antibodies with various concentrations of mAb TB31F is added. Subsequently, the oocyst prevalence and densities in the salivary glands of mosquitoes are compared between the experimental and control group by means of microscopy of the mosquito gut, parasite DNA detection or immune-assays [6, 7]. The percent transmission reducing activity (TRA) measured in the SMFA assay around which there is confidence that blocking activity is present (Minimum Effective Dose, MED) has been set by consensus at 80%. The presence of TB31F in serum is capable of blocking oocyst development in SMFA in rodent studies. Previously, 12.5 µg/mL of the mAb 5RF45.1 demonstrates complete blockage of the oocyst stage (NF54 strain *P. falciparum*) parasite development in SMFA (0/20 mosquitoes dissected were positive for oocyst stage parasites compared to 54/58 positive mosquitoes fed with gametocyte stage parasites in absence of any monoclonal antibody) [8]. >80% TRA (MED) in SMFA is generally first observed at MAb concentrations of between 3.3 to 10 µg/mL [TB31F Investigator's Brochure v1.0 19MAR2019]; sub-optimal TRA with increased variability between experiments is observed at concentrations between 1.1 to 3.3 µg/mL, which may be interpreted as the Minimum Anticipated Biological Effect Level

(MABEL).

The previously developed rat mAb 85RF45.1 has been humanized and the lead mAb selected for GMP-grade production is HC2+LC1 (hereinafter named TB31F). This first in human study aims to assess the safety and tolerability of monoclonal antibody TB31F administered intravenously at escalating dose levels and TB31F administered subcutaneously in healthy, malaria naive, adults. This study will also evaluate the pharmacokinetics of TB31F and the relationship of the pharmacokinetics with the functional activity of mAb TB31F in the standard membrane feeding assay. For this study, pharmacokinetics is defined as the study of the time course of drug absorption, distribution, metabolism, and excretion and pharmacodynamics refers to the functional relationship between drug concentration and the resulting effect measuring transmission-reducing activity in SMFA. Exploratively, we will examine for the anti- TB31F antibody response (anti-idiotypic) to mAb TB31F antibodies and develop an integrated population pharmacokinetic-pharmacodynamic model for TB31F.

Future use of this formulation of TB31F may include bridging the standard membrane feeding assay (SMFA) to direct skin feeds in a CHMI trans model or on asymptomatic naturally-infected carriers of *P. falciparum* gametocytes to determine whether the transmission-reducing properties of serum containing TB31F correlates with reduction in the proportion of mosquitoes infected with P. falciparum when Anopheles are fed directly on the skin of subjects previously administered TB31F mAb.

1.2 Rationale

The renewed focus on malaria elimination has increased the priority of research towards development of interventions to block malaria transmission [9, 10] . By interrupting transmission of malaria parasites in mosquito vectors, a reduction in the number of secondary infections in the community is expected with an overall reduction in disease and mortality [10]. Transmission blocking interventions will play an important role in the complete arrest of malaria transmission in endemic areas [6, 11, 12]. From a community perspective, deployment of transmission blocking drugs/biologics and transmission blocking vaccines (TBVs) will be an efficient complementary element in an integrated program of anti- malarial interventions, particularly for malaria elimination [10]. TBVs aim to actively induce specific and functional antibodies. Such antibodies are taken up by the mosquito during the blood meal and prevent parasite development in the mosquito gut. Alternatively, antibodies may be passively administrated, to inhibit parasite development, which is considered in areas with seasonal transmission or under specific circumstances such as malaria epidemics [6]. Therefore, availability of a monoclonal antibody to block transmission when used in combination with other malaria control measures is a critical and decisive step on the path to malaria elimination. The effector mechanism of TBV immunity relies completely on functional antibodies (abs) in the mosquito. As a first step in clinical development, it is critical to understand the pharmacokinetic- pharmacodynamic relationships of circulating antibodies with functional TBV activity. In addition to intravenous administration of escalating doses of TB31F in groups of subjects to assess these relationships over a wide range of serum concentrations, subcutaneous

administration will be performed in a final study group to allow better extrapolation of these relationships to envisaged use in target populations in field settings. This knowledge allows dose selection for future clinical studies.

2. HYPOTHESIS, OBJECTIVES, AND ENDPOINTS

2.1 Study Hypothesis

Monoclonal antibody TB31F will be safe and well tolerated in healthy adults.

2.2 Study Objectives

Primary objective:

- To assess the reactogenicity and safety after a single dose of mAb TB31F in terms of rates of solicited local AEs through 7 days post-administration, solicited general AEs through 28 days post- mAb administration, clinically significant hematological and biochemical laboratory abnormalities through 28 days post-mAb administration, unsolicited AEs through end of study (day 84), and serious adverse events (SAEs) through end of study.

Secondary objectives:

- To describe the serum pharmacokinetics (pK) of mAb TB31F at each dose level in healthy subjects.
- To assess the pharmacodynamics of mAb TB31F assessed as functional activity of serum in a standard membrane feeding assay at each dose level.

Exploratory objectives:

- To describe the anti-TB31F antibody response (anti-idiotypic) in terms of seropositivity rates and geometric mean titers (GMTs).
- To develop an integrated population pharmacokinetic-pharmacodynamic model for TB31F.

2.3 Study Endpoints

Primary outcome endpoints:

- Number and severity of solicited local adverse events of all severities from first product administration through day 7;
- Number and severity of solicited general AEs and clinically significant hematological and biochemical laboratory abnormalities from first product administration through day 28;
- Number and severity of unsolicited adverse events from first product administration through end of study;
- Occurrence of serious adverse events from first product administration through end of study.

Secondary outcome endpoints

- Serum pharmacokinetics at each dose level through end of study by ELISA;
- Percentage of transmission reducing activity at different time points by SMFA.

Exploratory outcome endpoints

- Anti-drug antibody (ADA) levels in study subjects after receiving study product through end of study;
- An integrated population pharmacokinetic-pharmacodynamic model by Monte Carlo simulations.

3. STUDY DESIGN

This first-in-human phase 1 trial is a proof-of-concept study whose purpose is to establish the safety and tolerability of TB31F administration in healthy subjects. The secondary objective of this trial is to quantify and measure the serum concentration and functional activity of TB31F in this setting. The trial is not designed to predict drug accumulation in humans upon repeated dosing, as this will be considered in the future with product characteristics that include improved potency and half-life extension strategies.

While we cannot predict with confidence the safety in human subjects, we have adopted a staggered trial design with escalating dosages of mAb TB31F and a single subcutaneous dosage of mAb TB31F. The trial will be carried out by the Radboud University Medical Center (Radboudumc). Four groups will receive a single dose of mAb TB31F administered by intravenous infusion and one group will receive a single dose of mAb TB31F administered subcutaneously. Group 1 (n=5) will receive 0.1 mg/kg TB31F, group 2 (n=5) will receive 1 mg/kg TB31F, group 3 (n=5) will receive 3 mg/kg TB31F, and group 4 (n=5) will receive 10 mg/kg mAb TB31F by intravenous infusion. Group 5 (n=5) will receive 100mg subcutaneously, divided equally between injection sites. Twenty-five (n=25) subjects will be enrolled, as well as 1 reserve subject per group.

TB31F administration within each group will be staggered such that the first subject in each group will be administered mAb TB31F and observed for the occurrence of any AEs. The second subject in each dose group will not receive their dose of TB31F sooner than 2 days after the first subject has received TB31F (Figure 3). For group 1, there will be a minimum of 48 hours between TB31F administration to each subsequent volunteer. For groups 2-5, the remaining three subjects in each dosage group will receive their TB31F dose no sooner than 2 days after the second subject has been administered TB31F, with at least a one-hour interval in administration of TB31F between each subject.

Escalation to the next higher dosage group will be dependent upon no safety signals arising as outlined in section 9.5.4 and will follow the same staggered algorithm as shown in figure 3. The SMC will review the safety data up to day 7 before proceeding to the next dosage group. The data to be reviewed before each dose escalation group includes the safety data (solicited and unsolicited AEs through day 7 post TB31F administration; see section 9.5.4) and clinically significant laboratory tests collected at 24 hours, 2 and 7 days following TB31F administration for all subjects in the specified dose group.

The decision to proceed at these defined time points to the next dosing group will be made by the study team upon the recommendation of the Safety Monitoring Committee (SMC) after reviewing the clinical and safety lab data available. Safety follow-up and collection of serum samples for pharmacokinetic and pharmacodynamic measurements will be done at following times: 0 hours, end

of infusion (EOI), 1, 3, 6 and 24 hours after product administration, and on days 2, 7, 14, 21, 28, 56 and 84 after product administration. Extra follow-up visits 4 and 10 days after administration for collection of serum/plasma for pharmacokinetic and pharmacodynamic measurements will be performed in group 5 (section 8.3.18). All subjects will be followed for approximately 84 days after mAb TB31F administration.

Table 1. Study groups

**Study groups**	**Number of subjects**	**Single Dose**	
		**Mtb31F**	**Administration Route**
Group 1	5	0.1 mg/kg	intravenous
Group 2	5	1 mg/kg	intravenous
Group 3	5	3 mg/kg	intravenous
Group 4	5	10 mg/kg	intravenous
Group 5	5	100 mg	subcutaneous

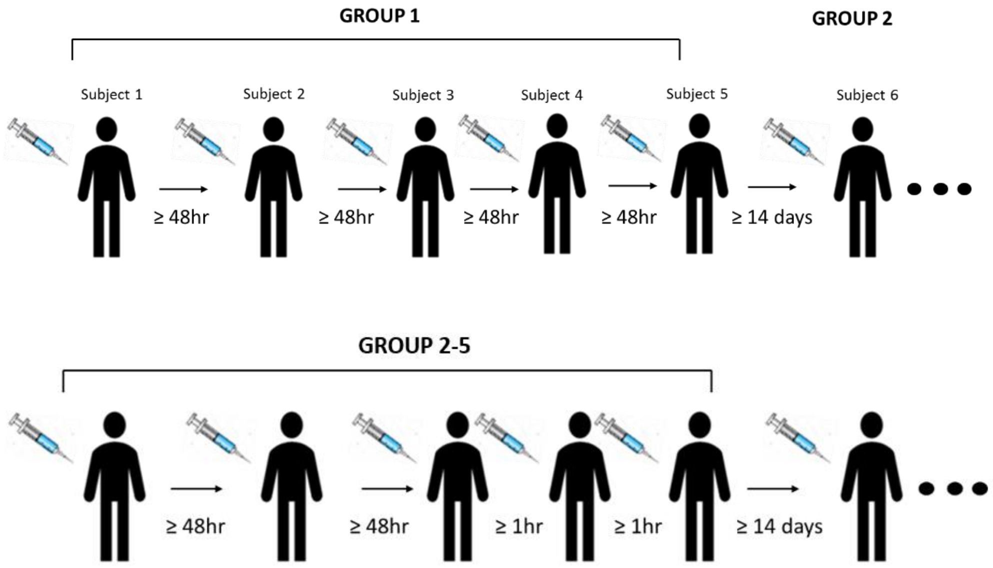

Figure 3 – Staggered dosing trial design for monoclonal antibody TB31F administration

For group 1, all subjects will receive a single dose of TB31F with a window of at least 48 hours. For groups 2-5, subject 1 and subject 2 will receive a single dose of TB31F with a window of at least 48 hours. Subjects 3, 4 and 5 of the same group will receive the same dose of mAb TB31F minimally 48 hours after subject 2. Escalation to the next dosage group will occur if no Group Holding Rules are met and upon review by the SMC.

3.1 Visit intervals

Permissible Within-Group visit intervals

The permissible intervals for TB31F administration between subjects enrolled in all groups are indicated in table 2. The permissible study visit intervals are indicated in table 3.

Table 2. Group intervals for Visit 2 (TB31F administration)

	Interval	Optimal length of interval	Allowed interval
Group 1	Subject 001 to Subject 002	2 days	≥ 2 days
	Subject 002 to Subject 003	2 days	≥ 2 days
	Subject 003 to Subject 004	2 days	≥ 2 days
	Subject 004 to Subject 005	2 days	≥ 2 days
	Subject 005 to Subject 006 (Interval between Group 1 to Group 2)	~14 days	≥ 14 days
Group 2	Subject 006 to Subject 007	2 days	≥ 2 days
	Subject 007 to Subject 008	2 days	≥ 2 days
	Subject 008 to Subject 009	1 hour	≥ 1 hour
	Subject 009 to Subject 010	1 hour	≥ 1 hour
	Subject 010 to Subject 011 (Interval between Group 2 to Group 3)	~14 days	≥ 14 days
Group 3	Subject 011 to Subject 012	2 days	≥ 2 days
	Subject 012 to Subject 013	2 days	≥ 2 days
	Subject 013 to Subject 014	1 hour	≥ 1 hour
	Subject 014 to Subject 015	1 hour	≥ 1 hour
	Subject 015 to Subject 016 (Interval between Group 3 to Group 4)	~14 days	≥ 14 days
Group 4	Subject 016 to Subject 017	2 days	≥ 2 days
	Subject 017 to Subject 018	2 days	≥ 2 days
	Subject 018 to Subject 019	1 hour	≥ 1 hour
	Subject 019 to Subject 020	1 hour	≥ 1 hour
	Subject 020 to Subject 021 (Interval between Group 4 to Group 5)	~14 days	≥ 14 days
Group 5	Subject 021 to Subject 022	2 days	≥ 2 days
	Subject 022 to Subject 023	2 days	≥ 2 days
	Subject 023 to Subject 024	1 hour	≥ 1 hour
	Subject 024 to Subject 025	1 hour	≥ 1 hour

Table 3. Permissible study visit intervals

Interval	Type of visits	Day of Visit	Allowed interval
Visit 1	Screening	-84 to -1	-84 to 0 days
Visit 2	Inclusion	Day -1	- 4 days
Visit 3	Administration	Day 0	± 6 hours
Visit 4	Follow-up	Day 1	± 6 hours
Visit 5	Follow-up	Day 2	± 6 hours
Visit 5a*	Follow-up	Day 4	± 1 day
Visit 6	Follow-up	Day 7	± 1 day
Visit 6a*	Follow-up	Day 10	± 1 day
Visit 7	Follow-up	Day 14	± 2 days
Visit 8	Follow-up	Day 21	± 2 days
Visit 9	Follow-up	Day 28	± 2 days
Visit 10	Follow-up	Day 56	± 5 days
Visit 11	(end-of-study visit)	Day 84	± 5 days

** Visits 5a and 6a will only be performed in group 5*

4. STUDY POPULATION

4.1 Population (base)

The study population will be comprised of adult male and female healthy subjects aged 18-35 at time of mAb administration. A total of 25 subjects will be enrolled to participate in the study as well as 5 reserve subjects (1 reserve subject per dosing group). The investigator will ensure that all subjects being considered for the study meet the eligibility criteria described in section 4.2 and 4.3. Subject eligibility is to be established and confirmed by checking all inclusion/exclusion criteria at both screening and inclusion (baseline). A relevant record of the eligibility criteria will be stored with the source documentation at the study site.

4.2 Inclusion criteria

Deviations from inclusion criteria are not allowed because they can potentially jeopardize the scientific integrity, regulatory acceptability of the study or subject safety. Therefore, adherence to the criteria as specified in the protocol is essential. All subjects must satisfy all of the following criteria at study entry:

- Subject must sign written informed consent to participate in the trial.
- Subject is able to understand planned study procedures and demonstrate comprehension of the protocol procedures and knowledge of study by passing a quiz (assessment of understanding). Subjects must score at least 80% correct on a multiple-choice quiz. If they do not score 80% on the initial quiz, the protocol information will be reviewed with them, and they will have the opportunity to retest.

- In the opinion of the investigator, the subject can and will comply with the requirements of the protocol.
- Subjects are available to attend all study visits and are reachable by phone throughout the entire study period from day -1 until day 84 (end of study).
- The subject will remain within reasonable travelling distance from the study center from day -1 until day +7 after mAb TB31F administration.
- Subject is a male or non-pregnant and non-lactating female age ≥ 18 and ≤ 35 years and in good health at time of mAb administration.
- Subject agrees to their general practitioner (GP) being informed about participation in the study and agrees to sign a form to request the release by their GP, and medical specialist when necessary, of any relevant medical information concerning possible contra-indications for participation in the study to the investigator(s).
- The subject agrees to refrain from blood donation to Sanquin or for other purposes throughout the study period according to current Sanquin guidelines.
- Female subjects of non-childbearing potential may be enrolled in the study. Non-childbearing potential is defined as pre-menarche, current bilateral tubal ligation or occlusion, hysterectomy, bilateral ovariectomy or post-menopause. All subjects must agree to use continuous adequate contraception* until 2 months after completion of the study. Female subjects must agree not to breastfeed from 30 days prior to mAb administration until 2 months after completion of the study. Female subject must have a negative pregnancy test at the inclusion visit.

Italic note: **Acceptable forms of female contraception include: established use of oral, injected or implanted hormonal contraceptives; intrauterine device or intrauterine system; barrier methods (condoms or diaphragm with additional spermicide); male partner's sterilization (with appropriate post-vasectomy documentation of absence of sperm in the ejaculate); true abstinence when this is in line with the preferred and usual lifestyle of the subject. Periodic abstinence (e.g., calendar, ovulation, symptothermal, post-ovulation methods) and withdrawal are not acceptable methods of contraception. Adequate contraception does not apply to subjects of childbearing potential with partners of the same sex.*

4.3 Exclusion criteria

Deviations from exclusion criteria are not allowed because they can potentially jeopardize the scientific integrity, regulatory acceptability of the study, or subject safety. Therefore, adherence to the criteria as specified in the protocol is essential. If any of the following exclusion criteria apply, the subject must not be included in the study:

1. Acute or chronic disease at time of TB31F administration, clinically significant pulmonary, cardiovascular, hepatic or renal functional abnormality, as determined by physical examination or laboratory screening tests:
    a. Acute disease is defined as the presence of a moderate or severe illness with or without

fever. Subjects with a minor illness on the day of TB31F administration will be (temporarily) excluded from participation, but may be re-evaluated for inclusion at a later date. Subjects with a positive SARS-CoV2 test at inclusion will be (temporarily) excluded from participation but may be re-evaluated for inclusion at a later date (following current Radboudumc guidelines).

b. Fever is defined as an oral, axillary or tympanic temperature ≥ 38.0°C (100.4°F). The preferred route for recording temperature in this study will be oral.

c. Any abnormal and clinically significant baseline laboratory screening tests of ALT, AST, creatinine, hemoglobin, platelet count or total white blood cell count, as defined in the protocol according to the FDA Toxicity Grading Scale for Healthy Adult and Adolescent Subjects Enrolled in Preventative Vaccine Clinical Trials (appendix 1).

2. History of malignancy of any organ system (other than localized basal cell carcinoma of the skin), treated or untreated, within the past 5 years.
3. Chronic use of i) immunosuppressive drugs, ii) antibiotics, iii) or other immune modifying drugs within three months prior to study onset (inhaled and topical corticosteroids and oral anti-histamines exempted) or expected use of such during the study period.
4. Positive urine toxicology test for cannabis, cocaine or amphetamines at screening or at inclusion.
5. Screening tests positive for Human Immunodeficiency Virus (HIV), active Hepatitis B Virus (HBV), Hepatitis C Virus (HCV).
6. Use of any investigational or non-registered product (drug or vaccine) during the study period other than the study product.
7. Participation in any other clinical study in the 30 days prior to the start of the study or during the study period.
8. Prior receipt of an investigational antimalarial monoclonal antibody.
9. History of adverse reactions to monoclonal antibodies.
10. Administration of immunoglobulin and/or any blood products within the three months preceding the first dose of study mAb or planned administration during the study period.
11. Any history of malaria, positive serology for *P. falciparum,* or previous participation in any malaria (vaccine) study or CHMI.
12. Body weight $\geq$ 115 kg.
13. Being an employee or student of the department of Medical Microbiology or Medium Care of the Radboudumc, or a person otherwise related to the investigator other than a professional relationship for clinical trial purpose only.
14. History of drug or alcohol abuse interfering with normal functioning in the period of one year prior to study onset.
15. Any other condition or situation that would, in the opinion of the investigator, place the subject

at an unacceptable risk of injury or render the subject unable to meet the requirements of the protocol.

4.4 Sample size calculation

TB31F is a first in human observational safety trial in healthy volunteers, designed to identify safety concerns associated with TB31F administration at different dosages. A total of twenty-five healthy adults (males and females) aged 18 – 35 will be divided over five groups. Each group will include five subjects. The study's original sample size of n=20 (four intravenous groups of n=5), was calculated to have a 90% chance of observing at least 1 event if the true rate of such an event is 10.87% or more; and there is a 90% chance of observing no events if the true rate is 0.52% or less. The 5th (subcutaneous) was subsequently pragmatically chosen to have the same size. Sample size for each group is appropriate for mAb administration Phase 1 trials based on similar trial designs for HIV, CMV, and anthrax monoclonal antibody prophylactic studies [13-15]. This is a small Phase 1 study; therefore, it is largely descriptive and is intended to provide primarily safety data of 4 escalating intravenous doses of mAb TB31F and one subcutaneous dose of mAb TB31F. Comparative statistics will be performed but will have low power to detect anything other than very large differences between the groups. 5 reserve subjects will be recruited (1 reserve subject per group).

5. TREATMENT OF SUBJECTS

5.1 Investigational product/treatment

Volunteers will sequentially receive a single administration of TB31F intravenously or subcutaneously. Five subjects will receive 0.1 mg/kg mAb TB31F intravenously (Group 1); five subjects will receive 1 mg/kg of mAb TB31F intravenously (Group 2), five subjects will receive 3 mg/kg of mAb TB31F intravenously (Group 3), five subjects will receive 10 mg/kg of mAb TB31F intravenously (Group 4), and five subjects will receive 100 mg (2mL) of mAb TB31F subcutaneously, divided equally between two injection sites of ~1mL each, in principle on the abdomen. For group 1 up till group 4, the mAb TB31F in frozen vials will be reconstituted with normal saline (concentration based on subject body weight) and put into an intravenous infusion bag or syringe per instructions included in the Pharmacy Manual. For Group 5, TB31F in a frozen vial (concentration 50 mg/mL) will be thawed, mixed and aspirated into a syringe per instructions included in the Pharmacy Manual. In case of tolerance issues in this group, TB31F may be diluted 1:1 in normal saline prior to administration, as described in the Pharmacy Manual, and sub-divided over additional sites of ~1mL each, as appropriate. There will be no placebo group.

5.2 Use of co-intervention (if applicable)

In order to be eligible for the study, subjects must abide to the inclusion and exclusion criteria as described in section 3.2 and 3.3 of this protocol. Therefore:

- subjects may not chronically use i) immunosuppressive drugs, ii) antibiotics, iii) or other immune modifying drugs within three months prior to study onset (inhaled and topical

corticosteroids and oral antihistamines exempted);

- male and female subjects must use continuous adequate contraception until 2 months after study completion. Females may not breastfeed from 30 days prior to mAb administration until 2 months after completion of the study;
- subjects must be reachable by phone (24/7) throughout the entire study period from day -1 until day +84;
- subjects will remain within reasonable travelling distance from the study center from day -1 until day +7 after mAb TB31F administration;
- subject must refrain from blood donation to Sanquin following current Sanquin guidelines;
- subjects may not receive any immunoglobulin and/or any blood products within three months preceding the first dose of study mAb or throughout the study, with the exception of TB31F;
- subjects may not participate in any other trials during the study period;
- subjects must always contact the trial physician if they experience any medical complaint throughout the entire study period;
- subjects may be required to respond to standardized Radboudumc-wide COVID-19 measures e.g. a questionnaire prior to study visits and/or undergo presumptive (e.g. at inclusion visit) and/or symptom-driven COVID-19 testing, e.g. a nasopharynx/throat swab for SARS-CoV2 PCR, in accordance with current Radboudumc-wide or MC-specific guidelines.

5.3 Medication available for adverse effects (if applicable)

All volunteers will receive premedication consisting of paracetamol (1000mg, oral) and clemastine (2mg, intravenous). Any infusion related reactions occurring during or after antibody administration will be treated according to physician discretion and following local treatment protocols. If mild symptoms arise during monoclonal TB31F administration in group 1 up till group 4, infusion rate will be slowed. If moderate to severe symptoms arise, infusion of TB31F will be stopped [16]. In case of an anaphylactic reaction, the current version of the RUMC anaphylaxis SOP will be followed.

6. INVESTIGATIONAL PRODUCT

6.1 Name and description of investigational products

- Product name: mAb TB31F
- Dosage form: liquid, total unit dose of the investigational product diluted in normal saline (groups 1-4)
- Route of administration: slow intravenous infusion or subcutaneous injection
- Physical description: liquid
- Study Drug Substance: TB31F
  Drug Substance was produced at Catalent, Pharma Solutions, Catalent Pharma Solutions, 726 Heartland Trail, Madison WI 5371
- Study Drug Product: TB31F, MassBiologics, 460LK Hill Street, Boston, MA 02126 USA

- Lot number and Label: 92-CM552-001A (figure 4)
- Product information: Intended for human use in adults as a transmission blocking measure against malaria transmission
- Alternatives to this IND Product or Study: At this time, there is no known approved product to afford the same potential protection from malaria.

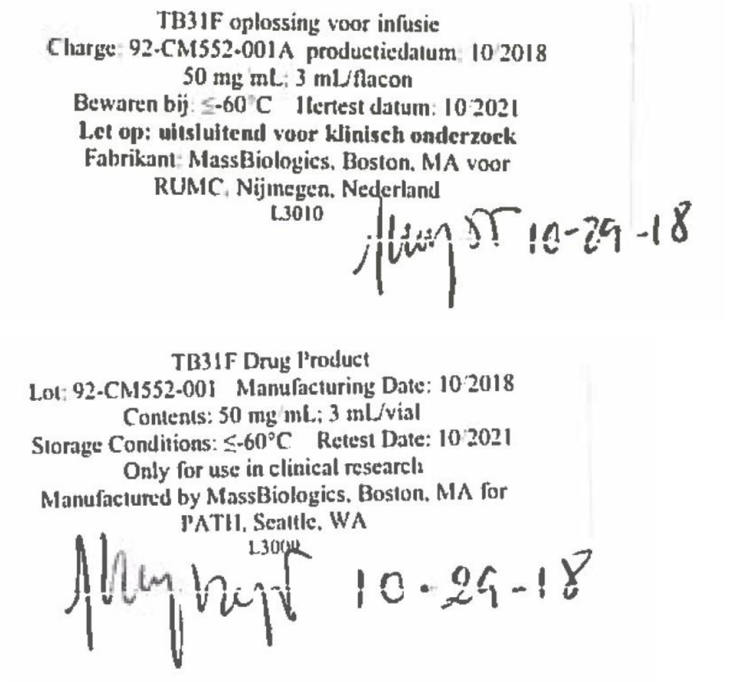

Figure 4 – Lot number and label in Dutch and English

6.2 Summary of findings from non-clinical studies

Preclinical data is described in more detail in the investigator’s brochure (IB). Briefly, Mab 85RF45.1 was isolated and characterized at the Radboudumc as a rat hybridoma cell line. In a standard membrane feeding assay (SMFA) at concentration of 12.5 ug/ml, the mAb 85RF45.1 demonstrated complete blockage of oocyst stage (NF54 strain *P. falciparum*) parasite development in *Anopheles* mosquitoes (0/20 mosquitoes dissected were positive for oocyst stage parasites compared to 54/58 positive mosquitoes fed with gametocyte stage parasites in absence of any monoclonal antibody) [8]. Mab 85RF45.1 was chosen as a candidate for humanization and further SMFA testing and was genetically modified (to rat-human chimeric mAb and humanized mAb). The three mAbs were evaluated concurrently in the SMFA assay, the results are shown in Figure 5. Transmission reducing activity of 80% or greater were reliably measured at a final concentration in the in vitro assay at ~ 3.5 ug/ml.

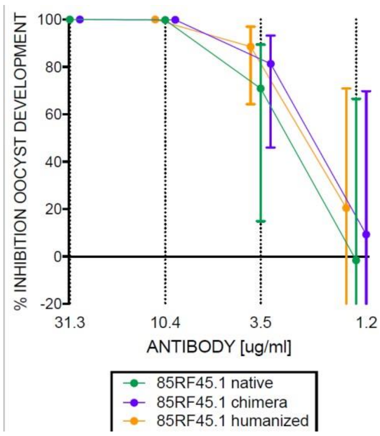

Figure 5: Inhibitory activity of native, chimeric and humanized form of 85RF45.1 antibody tested in SMFA.

Nine variants derived from three humanized heavy chains and three humanized light chains were sequenced and cloned at Lake Pharma, Inc. HC2+LC1 was selected as the lead mAb for GMP manufacture based on humanness scores and functional binding activities. The functional results of the lead mAb HC2+LC1 (hereafter named TB31F) are shown in Figure 6 below with TRA of 100% at 10ug/ml and ~80% at ~3 ug/ml. Indirect immunofluorescence against gametocytes was observed at 0.08 ug/ml mAb concentration.

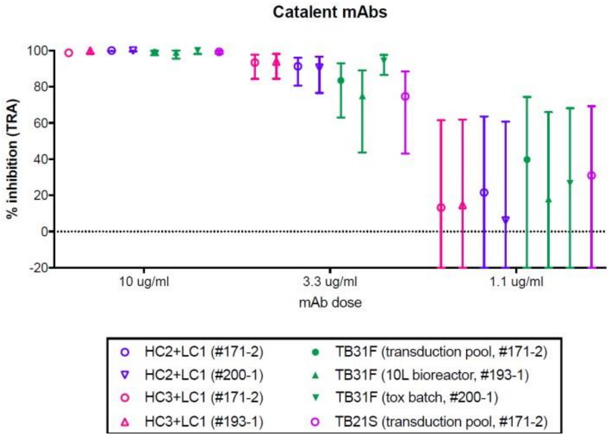

Figure 6: Transmission Reducing Activity of Catalent monoclonal antibodies measured at 1.1µg/ml, 3.3µg/ml and 10µg/ml.

6.2.1. Evaluation of Toxicity and Pharmacodynamics in Sprague Dawley Rats Administered TB31F

The purpose of the study was to determine the pharmacodynamics and toxicity of TB31F in Sprague Dawley rats. The rat is a standard and accepted species for pharmacokinetic and toxicity studies. The animals were assigned to a dose group using a procedure that stratifies animals across groups by body weight such that mean body weight of each group was not statistically different from any other group using analysis of variance (ANOVA). Forty male and forty female Sprague Dawley rats were allocated to one of six designated dose groups. The animals were administered one of five dose levels of TB31F or the saline control once via intravenous injection.

Table 4. Dosing groups of TB31F Administration in Sprague Dawley Rats

Group Number	Sex	Dose Level (mg/kg/day)
1	M/F	0 (Saline control)
2	M/F	18.75
3	M/F	37.5
4	M/F	75
5	M/F	150
6	M/F	240

For the toxicity subset of animals, 7 days post injection, 5 animals per sex had a full necropsy with all tissues assessed and any gross lesions collected; thymus, spleen and gross lesions were examined by a pathologist. For the pharmacodynamic (PD) subset of animals, blood was collected via cardiac puncture from one animal of each sex per group (groups 2-6) at 18 hours and 7 days post dose.

There were no significant effects on the body weight of male or female rats administered TB31F. There were no signs of toxicity noted during clinical observations for any treatment groups. Clinical observations were performed prior to administration, weekly, and at termination. There were no significant effects on the feed consumption of rats administered TB31F. There was no histological evidence of potential toxicity in the thymus or spleen associated with TB31F administration in male or female rats in this study.

Serum samples from the PD subset of animals were initially tested in a qualified SMFA with a standardized IgG concentration of 2.5 mg/mL. Inhibition was calculated from the direct count of oocysts in the midgut of mosquitoes. Oocysts were 99.6 to 100% inhibited compared to a negative control ($p = 0.001$). A second experiment was conducted based on set concentrations (1.1, 3.3, and 10 µg/mL) of the recovered TB31F antibody in the spiked human serum, erythrocyte, and gametocyte culture used in the SMFA. Only samples from animals assigned to Groups 2 (18.75 mg/kg) and 6 (240 mg/kg) were evaluated to bracket the dose levels evaluated in vivo. In these assays, we see a concentration dependent inhibition of oocysts, with full (100%) inhibition observed at 10 µg/mL, high levels of inhibition (91.3 to 100%) observed at 3.3 µg/mL, and low to no inhibition (-21.7 to 79.5%) observed at 1.1 µg/mL. There was no apparent difference between samples from the 18 hour or 7-day time points. These studies demonstrate that there is no loss of activity of TB31F when introduced into rat circulation and recovered, for up to 7 days.

6.2.2 Evaluation of Pharmacokinetics in Female Sprague Dawley Rats Administered TB31F

Forty female Sprague Dawley rats were allocated to one of two designated dose groups. Standard PK analyses were performed using provided comprehensive serum concentration data in rats following IV administration of 18.75 and 24.8 mg/kg of TB31F, a humanized monoclonal antibody (mAb). It is also known that antibody drugs demonstrate non-linear distribution, which is consistent with the PK of TB31F within a studied dose range. Blood was collected from the tail vein of each animal at various time points. The serum concentration profile of TB31F was

characterized by bi-phasic elimination kinetics. In the initial phase up to 12 hours post dosing, the serum concentration of TB31F declined rapidly over the first 2 hours, followed by a slower elimination with an initial elimination half-life (T1/2 INI) of 43 or 38 hours after administration of 18.75 or 24.8 mg/kg, respectively. Mean Cmax was not significantly different between the two dose groups (0.779 ± 0.244 mg/mL for 18.75 mg/kg vs. 0.884 ± 0.145 mg/mL for 24.8 mg/kg, $p>0.05$). The mean (± SD) serum concentrations of TB31F following the IV administration of 18.75 or 24.8 mg/kg are depicted in Figure 7.

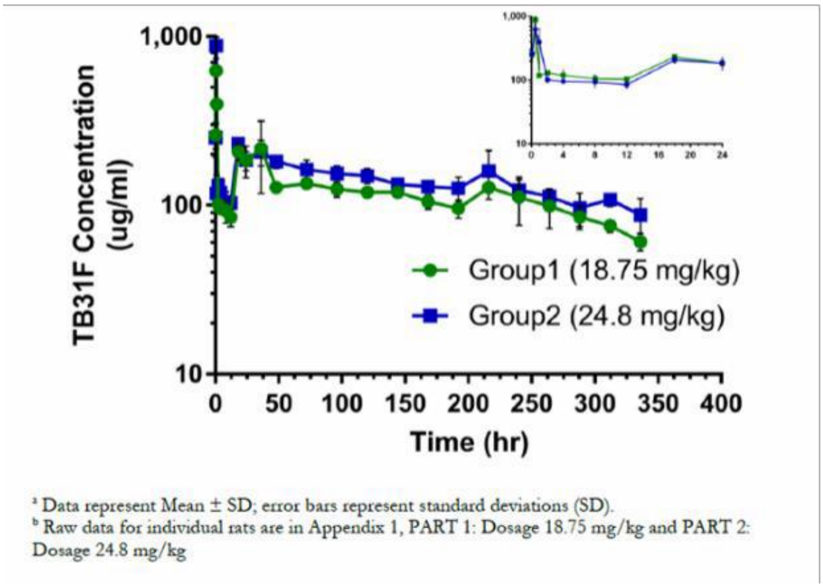

Figure 7: Serum TB31F time course for 18.75 and 24.8mg/kg IV dose

6.3 Summary of findings from clinical studies

There have been no reports of any other clinical trials of a monoclonal antibody that targets an antigen from the P. falciparum malaria parasite, which is passively administered in human subjects.

6.4 Summary of known and potential risks and benefits

The investigational study product, mAb TB31F, to be used in this experimental medicine study has not been administered previously to humans. All known risks and precautions described here are explained in detail in the informed consent documents.

6.4.1 Risk Assessment

The following section (Table 5) outlines the initial risk assessment and mitigation strategy for this study protocol.

Table 5. Risk Assessment

**Important Potential/Identified Risk**	**Data/Rationale for Risk**	**Mitigation Strategy**
**Investigational study product (mAb TB31F)**		
Important potential risk: **First-in-human unknown risk**	There is no prior experience of mAb TB31F or similar monoclonal antibodies to the Pfs45/48 malaria antigen in human subjects.	A dose escalating design will be used for Group 1 up till Group 4, starting with the lowest dose of 0.1 mg/kg. Study subjects will be observed closely by qualified clinicians, and emergency care will be immediately available to subjects. A minimum of 48 hours is required between product administration to the two sentinel subjects of each group. Group dose escalation will occur no sooner than 14 days according to the holding criteria.
Important potential risk: **Hypersensitivity (including anaphylaxis)**	As with other monoclonal antibody products, hypersensitivity and anaphylaxis to one or several components of the vaccine can rarely occur.	All study subjects will receive premedication. Subjects will be observed closely for at least 60 minutes following administration of the mAb TB31F product with appropriate medical treatment readily available in case of severe adverse events.
**Study procedures**		
**Pain when taking blood samples**	Because of the necessity to obtain frequent blood sampling for accurate PD/PK modeling, there is	Subjects will receive an intravenous catheter for frequent blood sampling. Subjects will be

	a risk of feeling faint, or experience mild pain, bruising, irritation or redness at the site where blood was taken. The amount of blood to be taken for sampling will not be harmful to the subject's health (~400mLs)	advised to inform or call the study doctor immediately if they have any side effects.
**Intravenous catheter placement**	The placement of an intravenous catheter in the subject's arm may cause the subject immediate pain at the site of catheter placement.	Experienced and qualified medical personnel will insert intravenous catheter and counsel the subject.
**Pregnancy risks**		
**Pregnancy and lactating females**	Risks of the mAb product to unborn babies are unknown at this time; pregnant females will be excluded from this study.	Study subjects should not become pregnant during the study and subjects will be counseled on appropriate use of contraceptives and tested before mAb TB31F administration. Lactating females will be excluded from this study.
**COVID-19 Related Risks**		
**Interaction between COVID-19 and TB31F administration**	Whether concurrent COVID-19 affects the safety of TB31F administration is unknown, but this appears unlikely, at least for mild infections (in severe infections, patients' hyper-inflammatory state might exacerbate adverse responses to this mAb). Similarly, it is unknown whether TB31F administration would exacerbate concurrent COVID-19, but considered also unlikely. Other study procedures (e.g. clemastine & paracetamol administration and blood draws) are not expected to affect COVID-19.	Subjects may be required to respond to a standardized Radboudumc-wide COVID-19 questionnaire prior to study visits and/or undergo presumptive (e.g. at inclusion visit) and/or symptom-driven COVID-19 testing, e.g. a nasopharynx/throat swab for SARS-CoV2 PCR, according to current Radboudumc-wide or MC-specific guidelines. Subjects with symptoms matching COVID-19, recent COVID-19 exposure and/or a positive SARS-CoV2 PCR will be (temporarily) ineligible for TB31F administration.

**Elevated risk of contracting COVID-19 infection through study participation**	Participating in the study may increase risk of exposure to SARS-CoV2, e.g. from study staff, other study subjects, hospital patients/staff or the general public.	Radboudumc has guidelines in place to minimize the risk of SARS-CoV2 transmission from staff and patients; current versions of these guidelines will also be adhered to by study staff and subjects. For example, subjects will be advised to avoid public transport to/from study visits where possible. SARS-CoV2 testing will be performed in accordance with current Radboudumc-wide and MC-specific guidelines.
**Risks to Study Personnel**		
**Needle-stick injuries**	The principal risk in the clinical setting is the handling of needles that may be contaminated with blood or body fluids and the associated risk of acquiring a blood-borne pathogen (including hepatitis B and C viruses and human immunodeficiency virus (HIV)).	Adherence to standard operating procedures (SOP) for working with infectious agents and universal precautions will reduce the risk of exposure. Subjects will be screened for Hepatitis B and C and HIV prior to inclusion. Individuals positive for HIV, hepatitis B and C are excluded from the study.

**Risk of acquiring SARS-CoV2 from study volunteers**	Study staff, other hospital staff and patients may be at (minimally) elevated risk of exposure to SARS-CoV2 from study participants.	Radboudumc has guidelines in place to minimize the risk of SARS-CoV2 transmission from patients; current versions of these guidelines will also be adhered to in the context of this study. These may include: requiring subjects to respond to a standardized Radboudumc-wide COVID-19 questionnaire prior to study visits; presumptive and/or symptom-driven COVID-19 testing of subjects; social distancing; use of personal protective equipment by staff; visits of subjects with symptoms matching COVID-19, recent COVID-19 exposure and/or a positive SARS-CoV2 PCR, may also be postponed or performed by telephone; where this might pose a risk to subjects themselves (e.g. for safety visits), the visit may go ahead with additional infection-preventions precautions, e.g. requiring subjects to wear a face mask.

Following IV administration of TB31F in groups 1-4, the following update to elements of the risk assessment was made:

Important Potential/Identified Risk	Data/Rationale for Risk	Mitigation Strategy
**Investigational study product (mAb TB31F)**		
Important potential risk: **Limited experience in humans**	mAb TB31F has been administered intravenously in a limited number of human subjects.(n=20)	Study subjects in group 5 will be observed closely by qualified clinicians, and emergency care will be immediately available to subjects. A minimum of 48 hours is required between product administration to the two sentinel subjects of each group.
**Local reactogenicity to SC administration of TB31F**	MAb TB31F has never previously been administered subcutaneously in humans. Subcutaneous administration of TB31F may cause the subject to experience temporary pain and/or burning sensation at the site of administration.	All study subjects will receive premedication with paracetamol and clemastine. Experienced and qualified medical personnel will place the subcutaneous administration and counsel the subject. Every injection will be carefully monitored to evaluate the occurrence of local adverse events. Subjects will be observed closely for at least 60 minutes following administration of the mAb TB31F product with appropriate medical treatment readily available in case of (severe) adverse events. In case of tolerability concerns, mAb TB31F may be diluted in normal saline for injections prior to subcutaneous administration, as described in the Pharmacy Manual.

6.4.2 Benefit assessment

No benefit is intended for study subjects; however, subjects may indirectly benefit from general medical evaluation and health screening procedures including testing for HIV, hepatitis B, and hepatitis C. Subject will be informed about the results of the screening and if necessary, they will be referred to their primary physician where they will receive counseling and further medical attention earlier than if they did not know of their disease status. Society as a whole may benefit from information learned through this study and individual subjects will contribute to the process of developing a mAb-based biological that might have a role in malaria elimination and transmission interruption against *P. falciparum* malaria in the future. Subjects will receive a financial compensation which is reasonable and in line with Dutch common practice (section 11.6).

6.4.3 Overall risk/benefit conclusion

The investigational mAb TB31F being used in this study is currently in a very early stage of clinical development and no efficacy has been demonstrated in humans. Taking into account the measures taken to minimize risk to subjects participating in this study, study participation will provide new knowledge and understanding of antibody-mediated transmission-reducing mechanisms against *P. falciparum* malaria infection.

6.5 Description and justification of dosages proposed in the clinical trial

MAb TB31F will be tested for the first time in humans and is designed to evaluate the safety and pharmacokinetics in a staggered dose escalation of four dosages intravenously (0.1 mg/kg; 1 mg/kg, 3 mg/kg, and 10 mg/kg) and one dosage subcutaneously (100 mg, 2mL) divided over 2 sites of ~1mL each. The rationale for the originally proposed intravenous dosages (groups 1-4) in this study is based on three considerations:

1) The initial starting concentration (0.1 mg/kg body weight) proposed in this protocol is extrapolated from animal safety data (calculations show in point 2, below) and is consistent with or below those in previous Phase 1 monoclonal antibodies directed against off-target (non- human) binding to other pathogens including CMV, HIV and anthrax [14, 15, 17]. Synagis® (palivizumab), an FDA-licensed mAb for the prevention of severe respiratory syncytial virus for which the target is a viral pathogen, is administered in infants at monthly doses of 15 mg/kg. Other investigational mAbs directed at pathogens that have been safely taken into efficacy trials include a mAb directed at Clostridium difficile toxin (bezlotoxumab) administered at a 10 mg/kg dosage and a mAb directed at hepatitis C virus administered at a 50 mg/kg dosage [18, 19]. The safety profile for such mAbs was determined to be acceptable. The dose escalation plan in this first-in-human Phase 1 study starts at 100 to 500-fold lower than typical dosages for other mAb directed at pathogens.
2) We based the initial starting dose on the EMA-recommended Minimum Anticipated Biological Effect Level (MABEL) approach, as well as taking into account calculations

recommended by the US FDA Maximum Recommended Starting Dose (MRSD) guidelines using a rat preclinical model. Sub-optimal (i.e., <80%) TRA (MABEL) is first detectable in SMFA between 1-2 µg/mL. A dose of 0.1 mg/Kg TB31F administered to 60 kg volunteer with a circulating volume of 5 L, would be expected to result in a peak concentration of ~1.2 µg/mL. Note that this approach results in a starting dose ~40-fold lower than that calculated using MRSD guidelines, based on the maximum dose of 240 mg/kg of TB31F tested in rat preclinical toxicity study in which no observed adverse effect levels (NOAELs) were observed in the tested rat species. This NOAEL of 240 mg/kg was used to convert to the Human Equivalent Dose (HED). HED = NOAEL rat of 240 mg/kg * 0.16 = 38.4 mg/kg assuming a 0.23 kg rat and a 60 kg human. The default safety factor that should normally be used is 10-fold. The HED calculation resulted in 3.84 mg/kg. The proposed starting dose of 0.1 mg/kg in Group 1 is thus approximately 40 times lower than the calculated HED.

3) We selected a range of four concentrations based on dose escalation from the initial dose of 0.1 mg/kg to the highest dose of 10 mg/kg in order to provide meaningful separation of exposure between dose groups for safety evaluation of adverse events and for PK assessment of monoclonal antibody concentration over time. A model-based meta-analysis of monoclonal antibody pharmacokinetics of groups receiving different dosages was used to estimate serum concentrations following TB31F infusion over time.

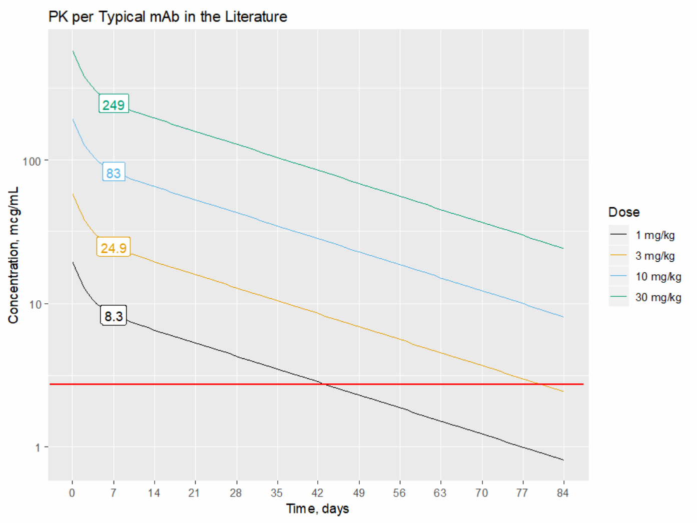

Figure 8 – Pharmacokinetic properties of monoclonal antibodies administered at 1 to 30 mg/kg

Based on known functional activity of TB31F serum concentrations in SMFA, we predict detectable TRA will be observed in all groups administered TB31F mAb at peak concentration following administration and >80% TRA (that corresponds with blocking activity) will be achieved in the four highest dose groups. By the end of the study (day 84), we would predict that subjects in groups receiving the highest dosage of TB31F (10 mg/kg) would still exhibit transmission blocking activity while subjects in Groups 1 and 2 that were administered TB31F at 0.1 and 1 mg/kg would no longer demonstrate inhibitory activity.

We chose a broad range of dosages to answer critical questions with respect to the functional in vivo functional properties of TB31F:

- What is the range of mAb TB31F human serum concentrations in subjects receiving five different dosages of the mAb?
- What is the dynamic range of concentration of mAb TB31F associated with SMFA functional activity at specific time points?
- What is the PK profile of this mAb with respect to half-life of serum concentrations that exceed the minimal inhibitory concentration required for >80% inhibition of oocyst development in the mosquito and 80% inhibition of proportion of mosquito infected with one or more oocysts?

An additional consideration was added regarding subcutaneous dosage in group 5, following IV administration in groups 1-4.

4) A fixed dose of 100 mg was selected as proof of concept. The dosage of 100 mg mAb TB31F in subjects of 50-90 kg is equivalent to a dosage of 1.1-2 mg/kg. The bioavailability of mAb is estimated to be between 50 and 80% after subcutaneous administration. The subcutaneous dose is therefore expected to approximate to 1 mg/kg IV. Preliminary analysis in group 2 (1 mg/kg) showed that TRA of >95% is maintained up to day 28 after TB31F administration.
5) TB31F will be applied in undiluted form for subcutaneous administration (group 5). There are no formal requirements for the upper limit of osmolality of parenteral drugs [20] and administration of mAbs at higher tonicity has shown to improve subcutaneous absorption of monoclonal antibodies [21]. Intramuscular administration of monoclonal antibodies (which is considered more painful than subcutaneous administration) has shown to be safe and tolerable up to 1100mOsm/kg with limited pain and discomfort [22]. It is therefore to be expected that single small (1mL) undiluted doses of TB31F (pH 6.0 and osmolality ~700mOsm/kg) will have acceptable tolerability. Should subcutaneous administration not be tolerated well, a 1:1 dilution with NaCl is described in the Pharmacy Manual.

6.6 Dosages, dosage modifications and method of administration

See Pharmacy manual for drug preparation. One objective of the study is to provide a reference point for future passive transfer trials of monoclonal anti-Pfs48/45 antibodies and as such the TB31F mAb will be administered to subjects through an intravenous catheter in all subjects of group 1 up till group 4 and will be administered subcutaneously in subjects of group 5. The intravenous catheter will be placed on the day of mAb TB31F administration. MAb TB31F will be added to normal saline in 20cc or 50cc syringes, or 100mL bags and connected to the intravenous catheter in subjects of group 1 up till group 4 (see Pharmacy Manual for drug preparation).

MAb TB31F in frozen vials will be reconstituted with normal saline (concentration based on

subject body weight) for intravenous infusion in group 1 up till group 4. Intravenous infusion in these first four groups will occur slowly with a controllable infusion pump. Severe hypersensitivity reactions are rare, provided subjects receive close monitoring, and prompt intervention when symptoms occur. Mild-to-moderate reactions can be managed by temporary infusion interruption, reduction of the infusion rate, and symptom management. Intravenous administration of TB31F mAb will be administered using a step-wise approach (see Appendix 2). Infusion rates will be reduced by 50% for mild reactions; and terminated for severe infusion reactions.

In group 5, subcutaneous injection of 2 mL undiluted mAb TB31F will occur slowly with a syringe divided over 2 sites of ~1mL each, in principle on the abdomen. In case of tolerance issues, TB31F may be diluted 1:1 in normal saline prior to administration, as described in the Pharmacy Manual, and sub-divided over additional sites of ~1mL each, as appropriate.

Table 6. TB31F formulation in groups 1-4

Treatment	Product name	Formulation	Presentation	Total volume to be administered (TB31F + NaCl)	Number of doses
mAb TB31F	mAb TB31F	mAb TB31F drug substance is formulated in 10 mM histidine, 150 mM sodium chloride, 10% (w/v) sucrose, 25 mM arginine, 0.01% (w/v) polysorbate 20, pH 6.0 at a target concentration of 50 mg/mL.	3mL frozen liquid	~6-46 mL	1
	Normal saline	0.9% Sodium chloride solution	Liquid in vial		

Table 6a. TB31F formulation in group 5

Treatment	Product name	Formulation	Presentation	Total volume to be administered (TB31F)	Number of doses
mAb TB31F	mAb TB31F	mAb TB31F drug substance is formulated in 10 mM histidine, 150 mM sodium chloride, 10% (w/v) sucrose, 25 mM arginine, 0.01% (w/v) polysorbate 20, pH 6.0 at a target concentration of 50 mg/mL.	3mL frozen liquid	2mL (1mL per site)*	2*

* In case of tolerance issues, TB31F may be diluted 1:1 in normal saline prior to administration, as described in the Pharmacy Manual. The total volume (TB31F + NaCl) to be administered in this case will be 4mL, sub-divided over additional sites of ~1mL each, as appropriate.

6.7 Preparation and labeling of Investigational Medicinal Product

6.7.1 Presentation and formulation

The mAb TB31F is presented as 3mL frozen concentrated liquid in a 10mL container. Prior to administration, the mAb TB31F will be prepared for intravenous infusion or subcutaneous injection per the procedures set forth in the Pharmacy Manual. Briefly, for groups 1 up till group 4 the study product will be removed from frozen storage, thawed and diluted with sterile saline solution in an IV bag or syringe. After dilution with sterile saline, the bag is mixed. The prepared diluted investigational product may be stored at room temperature condition for up to 4 hours. For group 5 the study product will be removed from frozen storage, thawed and 1 mL added per syringe (2 syringes of 1mL in total). In case of tolerance issues, TB31F may be diluted 1:1 in normal saline prior to administration, as described in the Pharmacy Manual.

6.7.2 Stability and storage

MAb TB31F must be stored at ≤-60°C prior to dilution in a safe and locked place. Access to the storage space should be limited to authorized study personnel. The storage conditions will be assessed during pre-trial monitoring visits. The storage temperature should be continuously monitored with calibrated temperature monitoring device(s) and recorded. Temperature excursions must be reported in degrees Celsius. Once the dilution process is completed, the prepared study product can be stored at ambient temperature and administered within 4 hours. Any temperature excursion outside the ranges specified in the Pharmacy Manual must be reported within 24 hours of site awareness to PATH. The impacted study vaccines must not be used and must be stored in quarantine at label temperature conditions until usage approval has been obtained from PATH.

6.7.3 Dose preparation and administration

Dose preparation, as described in the Pharmacy Manual, will be carried out by qualified personnel under aseptic conditions. The investigational product will be prepared to administer in a 100mL infusion bag or a 20cc or 50cc syringe (groups 1-4) and a 5cc (or smaller) syringe for group 5, labeled with subject number, and administered by a study staff member and dispensed to the investigator/medical doctor. Receipt and disposal of the investigational products will be done according to RUMC SOPs. All vials study product mAb TB31F will be stored at ≤-60°C, and storage temperatures will be recorded daily with validated temperature monitoring devices. Any temperature deviations in the vials mAb TB31F outside the defined range (≤-60°C) must be reported to the sponsor as soon as detected. Following exposure to a deviation in temperature, study product will not be used until written approval has been given by the sponsor. Commercial sterile normal saline will be purchased for use as diluent for intravenous infusion of mAb TB31F in groups 1 to 4.

6.7.4 Labeling

The study mAb TB31F is labeled and packed according to applicable regulatory requirements. Box label (Figure 9) and vial label are depicted below in English and Dutch. As product administration will take place in the Netherlands, there will be at least a Dutch label on each box and vial.

TB31F Geneesmiddel
Lot: 92-CM552-001A
productiedatum: 10/2018
bewaarcondities: ≤ -60°C
Use According to Protocol
Let op: uitsluitend voor klinisch onderzoek
Geproduceerd door: MassBiologics, Boston, MA voor PATH, Seattle, WA
Totaal:_______Doos_______van de ______

TB31F Drug Product
Lot: 92-CM552-001A
Date of Manufacture: 10/2018
Storage Conditions: ≤ -60°C
Use According to Protocol
Caution: New Drug Limited by Federal Law to Investigational Use
Manufactured by: MassBiologics, Boston, MA for PATH, Seattle, WA
QTY.:_______Box_______of _________

TB31F Geneesmiddel
LXXXX Lot: 92-CM552-001A
inhoud: 50 mg/mL; 3 mL/vial
productiedatum: MM/YYYY
bewaarcondities: < -60°C
Let op: uitsluitend voor klinisch onderzoek
Geproduceerd door MassBiologics, Boston, MA voor PATH, Seattle, WA

TB31F Drug Product
LXXXX Lot: 92-CM552-001A
Contents: 50 mg/mL; 3 mL/vial
Date of Manufacture: MM/YYYY
Storage Conditions: ≤ -60°C
Caution: New Drug Limited by Federal Law to Investigational Use
Manufactured by MassBiologics, Boston, MA for PATH, Seattle, WA

Figure 9 – TB31F vial labels (top) and box labels (bottom) in Dutch and English

6.8 Drug accountability

The site pharmacist will maintain complete records of all study products received from the sponsor and an accurate record of the randomization codes, inventory, and an accountability record of mAb TB31F supplies for this study. The site pharmacist will also ensure the security of these documents. Partially used vials will not be used for human administration or for in vitro experimental studies. At the end of the study, the site will receive instruction from the Sponsor regarding the final disposition of any remaining study products.

7. NON-INVESTIGATIONAL PRODUCT

All volunteers will be treated with paracetamol (1000mg, orally) and clemastine (2mg, intravenously) prior to TB31F administration.

8. METHODS

8.1 Study parameters/endpoints

8.1.1 Primary study parameters/endpoints

- Occurrence of solicited local adverse events of all severities from first product

administration through day 7;

- Occurrence of solicited general AEs and clinically significant hematological and biochemical laboratory abnormalities from first product administration through day 28;
- Occurrence of unsolicited adverse events from first product administration through end of study;
- Occurrence of serious adverse events from first product administration through end of study.

8.1.2 Secondary study parameters/endpoints

- Pharmacokinetic measurements at each dose level through end of study by ELISA;
- Percentage of transmission reducing activity (TRA) at different time points by SMFA.

8.1.3 Exploratory study parameters/endpoints

- Anti-drug antibody (ADA) levels in study subjects after receiving study product through end of study;
- An integrated population pharmacokinetic-pharmacodynamic model by Monte Carlo simulations.

8.2 Randomization, blinding and treatment allocation

This will be an open-label trial to enable appropriate safety evaluation prior to proceeding to the next dose group. Randomization will not be used in this study. Subjects may choose in which of the five groups they wish to be enrolled. The time of notification and treatment group will be documented in the electronic case report form (eCRF).

If the enrollment criteria are met, the eligible subjects will be entered in the study. Screening applicants will receive a screening number of two letters and a corresponding volunteer number as soon as they return their application form. Study numbers will not represent or include any private identifiable information.

8.3 Study schedule and procedures

It is the responsibility of the investigator to ensure only medically qualified study staff perform medical assessments and procedures which require medical training. Results of assessments should be reviewed with the subject, as appropriate, in a timely manner. It is also the responsibility of the investigator to ensure strict observance of the intervals between visits/procedures. These intervals are precisely defined for subjects in accordance with the protocol and are reflected in the study schedule outlined in section 8.3.18. In the context of the ongoing COVID-19 outbreak, the clinical investigator will follow current local and national guidelines concerning the mitigation of SARS-CoV-2 transmission and dedicated to minimizing the risk of exposure of study volunteers, study personnel, hospital staff & patients and the general public. This applies to all study visits. Examples of such mitigation measures are listed in Table 5.

8.3.1 Recruitment of subjects

The purpose of recruitment will be to obtain 25 eligible subjects for participation in this trial plus 5 reserve subjects (1 reserve subject per group). In order to recruit sufficient subjects, advertisements will be placed in prominent public places, as well as on the University intranet. Short presentations will be held at university before class lectures where possible. Furthermore, the study will be advertised on social media and local newspapers. The advertisement text will indicate a telephone number to call, an e-mail address, and a website (www.malariavaccin.nl) to request further information. Interested subjects can register for the information meetings by filling in an online form which includes questions regarding their health status, use of medication, and participation in previous scientific studies. All subjects with an interest in participation will be advised to join one for the information meetings, where they will receive information on study participation and the rights and duties of subjects, as well as the corresponding documents (the study information sheet and study schedule, the application form, general information brochure on medical-scientific research from the Ministry of Health, Welfare and Sport, and the insurance text). If they are still interested after the information meeting and/or after reading the documents, an appointment for a screening visit will be made. The screening visit will be planned at least 72 hours after the subject receives the information sheet and informed consent form. Since the information meeting is not mandatory, the documents can be e-mailed to subjects as well. Applicants will receive a screening number of two letters and a corresponding volunteer number as soon as they return their application form. This number will be used for source documents. This volunteer code will not contain any identifiable information.

8.3.2 Screening visit (visit 1)

The screening visit will take place within 84 days before the inclusion visit. The screening appointment for individual subjects is planned at least 72 hours after the subject has received the information sheet and the informed consent form. The purpose of the screening visit is to determine whether interested subjects are eligible for participation, to provide and clarify study information to the volunteer and to answer any questions the subjects may have. The trial physician is responsible for providing the study information and performing the medical screening. A study nurse may draw venous blood, and collect vital parameters.
Upon arrival for the screening visit, the volunteer is asked to fill out a medical questionnaire. Subsequently, and prior to any screening activities, study staff will review the informed consent process with the volunteer. The possibility of withdrawal from the study, at any time and without any declaration of the reason, will be pointed out to the subjects. The subject will be required to pass a (short) written quiz about the study in order to ensure the study subjects has sufficient understanding of what participation entails. The investigator, or a person designated by the investigator, will fully inform the volunteer of all pertinent aspects of the study and individual consent will be documented by a signature. Subjects may only participate in screening if they have signed the informed consent form. All subjects must consent to an HIV, hepatitis B and hepatitis C serological screening, urine toxicology and for females also a pregnancy test. Subjects who sign the

informed consent will undergo complete screening (section 8.3.8). After subject consents, the below activities will occur:

- Patient history: the answers to the medical questionnaire are discussed with the volunteer and clarified where needed. The subject will be further interviewed to collect demographic data, medical history including details of any chronic or recurrent medical and psychiatric conditions and use of adequate contraception;
- Study information: the study schedule and study rules are discussed with the subject and any questions are answered;
- A physical examination including vital signs, height and weight will be performed;
- Blood specimens will be collected for routine clinical laboratory testing of biochemical and hematological parameters, as well as HIV, hepatitis B and hepatitis C serological screening;
- A urine specimen will be collected for toxicology screening and for females a pregnancy test if the subject is of childbearing potential;
- Consent to inform the general practitioner of study participation, and if necessary a medical specialist, will be signed by the subject and sent after screening.

If physical examination, vital signs or laboratory values are out of the normal range, a repeat measure may be obtained as deemed necessary by the investigators. The medical history, physical examination, and laboratory findings for subjects will be recorded in the source screening data documents. All subjects will be asked to supply a phone number of a partner or roommate who may be contacted in case of emergency. Concomitant medications is recorded at all study visits.
All results of the screening will be reviewed with the subject. Subjects are informed by phone if they have satisfied all the inclusion criteria. If clinically significant abnormalities are identified during screening, subjects will be referred to their primary health provider or appropriate medical center. If identified during the study, subjects may be asked to return to the study site for further evaluation, including clinical evaluation and repeat laboratory testing as warranted.

8.3.3 Inclusion visit (visit 2)

Subjects meeting the eligibility criteria during screening (section 4.2 and 4.3) will be invited back for enrolment into the study at the inclusion visit, which will occur prior to the planned administration day. Baseline assessments will be taken on the inclusion day. For each subject, study start (day 0) will be defined as the day of mAb administration. For subjects that do not show up for inclusion visit (visit 2), alternate subjects may be used. At study inclusion, the following activities will occur:

- Patient history will be taken and all adverse events that have occurred since screening will be noted. Only subjects who still meet the inclusion criteria will be included to receive mAb TB31F;
- Blood specimens will be collected for routine clinical laboratory testing of biochemical and hematological parameters;
- A urine specimen will be collected for toxicology screening and for females a pregnancy test

if the subject is of childbearing potential;

- Subject will be issued symptom diaries and a thermometer to record any local and systemic symptoms and medication use;
- All subjects will be issued an emergency notification card that details their participation in the study and provides contact phone numbers of the investigators.
- Since TB31F is administered at the Medium Care, subject may undergo presumptive testing for SARS-CoV2 (e.g. nasopharynx/throat swab for PCR) at inclusion in accordance with current Radboudumc MC guidelines.

8.3.4 mAb TB31F inoculation visit (visit 3)

The purpose of this visit is to inoculate enrolled subjects with mAb TB31F. On the day of mAb administration, the subjects will be admitted to the Medium Care Unit for approximately 8-10 hours. Subjects will be briefly assessed regarding any new medical events since the inclusion visit. Temperature, blood pressure, and pulse rate will be recorded. A repeat measurement of body weight may be performed. To confirm eligibility, results of all assessments will be reviewed. If the subject is still eligible for participation, the following activities will occur as detailed in section 8.3.18:

- The subject will have one or two intravenous dwelling catheters inserted into the antecubital vein of the arm.
- The subject will receive premedication: 1000mg paracetamol orally and 2mg clemastine intravenously.
- mAb TB31F product will be prepared by pharmacy staff based on group assignment and study subject weight in groups 1 up till group 4.
- mAb TB31F will be administered by slow intravenous drip (see Appendix 2 for infusion rates) in group 1 up till group 4. The infusion rate will be reduced by 50% for mild reactions; and terminated for severe infusion reactions. MAb TB31F will be administered subcutaneously by slow injection in group 5, divided equally between two injection sites (1mL per site). Subjects will be observed directly for at least 60 minutes after the infusion. One intravenous dwelling catheter may be removed following drug product administration.
- Collection of serum/plasma for pharmacokinetic and pharmacodynamic measurements will be done at the following time points: 0 hours, EOI and 1, 3, and 6 hours after product administration. Subjects may leave the Medium Care Unit after 6 hours and return the next day to the clinical research center for the 24-hour blood sampling time point.
- Vital signs, adverse events (solicited and unsolicited) and blood will be collected at each time point as indicated in section 8.3.18.
- A list of solicited local and systemic symptoms will be reviewed with the subject at 0, EOI, 1, 3 and 6 hours (day 1) and 24 hours (day 2). If the occurrence of an adverse event or use of medication is confirmed by the study physician, it is recorded in the clinical trial database.

- After leaving the hospital, subjects will be asked to examine the site of infusion and record local signs and symptoms for seven days including bruising, erythema, swelling or induration. Subjects will be asked to measure their oral temperature daily until day 7. Subjects will be asked to record any AEs in the memory aid booklet until day 84. At the end of the study, the memory aid will be collected and kept as source data with the subject's study file.

All inoculations are performed by a trained nurse under the supervision of one of the clinical investigators. Another clinical investigator will be on call in case of emergency. Local hospital procedures will be followed. Study subjects will be monitored at the Medium Care Unit on the day of TB31F administration. The venous catheter will be removed at the end of visit 3 (day of product administration) and subjects will be discharged from the Medium Care Unit. Follow-up visits will take place as described in section 8.3.18.

8.3.5 Follow-up after mAb TB31F administration (visits 4-12)

Follow-up visits will be carried out on an outpatient basis at the study center. Subject will be asked to examine the site of injection and record local signs and symptoms in their memory aid booklet (including bruising, erythema, swelling or induration) for seven days after injection as well as systemic symptoms throughout day 28. Subjects will also be asked to measure their body temperature in the mornings for 6 consecutive days after mAb TB31F administration. The subject diary will be reviewed at each study visit and used as a base for discussion of possible local and systemic adverse events or medication use. If the occurrence of an adverse event or use of medication is confirmed by the study physician, it is recorded in the clinical trial database. At the end of the study, the diary will be collected and kept as source data with the subject's study file. Follow-up visits *not* requiring blood collection for safety endpoints may be carried out by telephone, or at home, instead of at the clinical research center for subjects with suspected or confirmed SARS-CoV2 infection, until such time as they are considered no longer (potentially) contagious in accordance with current Radboudumc-wide guidelines. In such cases, it may not be possible to obtain blood samples for non-safety endpoints (PK, ADA & SMFA).

During the study period subjects will be instructed to call the trial physicians at any time if they experience severe symptoms or symptoms related to COVID-19. If clinically significant abnormalities are identified during the study, subjects may be asked to return to the study site for further evaluation, including clinical evaluation and repeat laboratory testing as warranted. The trial physician can decide to initiate any additional diagnostics (including safety laboratory evaluations) at all times. For unexpected laboratory abnormalities, the laboratory test will be repeated. If there is any ambiguity regarding the decision to include or exclude a volunteer, a trial physician will discuss the case with the local safety monitor.

8.3.6 Unscheduled visits

Subjects may need to present to the study center during operating hours for an unscheduled

visit should they experience any other AE that requires evaluation by the trial clinician. Data for any examinations performed on the subject at an unscheduled visit must be recorded in the eCRF. If an unscheduled visit is performed, the procedures for the next following visit should not be made earlier than scheduled above.

8.3.7 Medical history

The trial clinician will review the medical history of potential study subjects during the screening visit. The starting point will be a medical questionnaire the subjects have filled in before the screening. Particular attention will be paid to:

- Current or recent (within the previous two weeks) acute respiratory illness with or without fever, including symptoms associated with COVID-19;
- Recent receipt of immune globulin or other blood products, or injected corticosteroids or other immune modulator therapy within the previous six months;
- Hypersensitivity of any kind;
- Clinically relevant history of cardiovascular, renal, gastrointestinal, hematological, dermatological, endocrine, neurological or immunological diseases;
- Known or suspected immunologic function impairment of any kind and/or known HIV infection, hepatitis B or hepatitis C infection;
- Mental illness;
- Tobacco, alcohol, or drug use;
- Medication use in the past 6 months;
- For women, pregnancy and contraceptive use and/or history of surgical sterility. For males, use of contraception;

8.3.8 Physical Examination

A physical examination will be performed during screening, including the examination of general appearance, skin, neck, eyes, throat, lungs, heart, abdomen, back and extremities, and a routine vascular and neurological examination. At inclusion, a repeat measurement of body weight will be obtained. During follow-up visits a focused physical examination will be performed if deemed necessary by the trial physician.

8.3.9 Height and Weight

Height (cm) and body weight (to the nearest kilogram [kg] in indoor clothing, but without shoes) will be measured at the screening visit. Body mass index (BMI) will be calculated using the following formula: $BMI = \text{Body weight (kg)} / [\text{height (m)}]^2$ and converted to an integer. Body weight will be repeated at the inclusion visit to be used for the preparation of mAb TB31F in group 1 up till group 4.

8.3.10 Vital Signs

Vital signs including body temperature (degrees Celsius), blood pressure (BP, millimeters of mercury) and pulse measurements (beats per minute) will be recorded at the screening visit. Additional measurements will take place at the discretion of the physician.

Systolic and diastolic BP will be measured while the subject is sitting, with the back supported and both feet placed on the floor, using an automated validated device, with an appropriately sized cuff. In case the cuff sizes available are not large enough for the subject's arm circumference, a sphygmomanometer with an appropriately sized cuff may be used.

If vital signs are out-of-range at screening or baseline, the investigator may obtain two additional readings, so that a total of up to three consecutive assessments are made, with the subject seated quietly for approximately five minutes preceding each repeat assessment. At least the last reading must be within the normal range in order for the subject to qualify.

Temperature, blood pressure and pulse are measured as part of the physical examination at screening and inclusion. Additionally, subjects will be given a thermometer to measure their oral temperature on a daily basis for one week after TB31F administration, which they will collect in their study diaries.

8.3.11 **Laboratory Evaluations**

During the study, blood samples will be drawn for screening, safety and research purposes. The blood sampling schedule in the flowchart (section 8.3.18) shows the maximum amounts of blood that will be drawn. Following universal precautions, blood will be collected by venipuncture into vacutainer tubes. Blood specimens will be affixed with coded labels that link the specimen to the subject, specimen type, specimen collection date, and time-point. The specimen collection tube must be appropriate for the type of specimen required. The cumulative blood draw for each subject over the entire course of study participation is approximately 400mL.

Biological safety parameters will be measured on plasma or serum samples at the central laboratory of the RUMC. In the case where a laboratory assessment is outside the reference range, a decision regarding whether the result is of clinical significance or not shall be made by the investigator and shall be based, in part, upon the nature and degree of the observed abnormality. The assessment may first be repeated for confirmation. All abnormalities will be documented in the source documents, including clinical considerations.

<u>Pharmacokinetic and functional analysis of transmission reduction activity</u>

Pharmacokinetic (pK) sampling will establish the dose-response curve for each subject enrolled in this Phase 1 study. The serum concentrations of TB31F will be measured by an ELISA against a recombinant epitope R0.6C of the Pfs48/45 antigen at each sampling time point. Assessment of TB31F concentrations in the serum from subjects will be performed by the Human Immunology Laboratory at Imperial College London. Functional transmission-blocking activity will be determined at defined intervals as per protocol using SMFA assays performed at Radboudumc in Nijmegen the Netherlands

<u>Complete Blood Count (CBC)</u>

Complete hematology tests, including hemoglobin, hematocrit, red blood cell count, white blood cell count with differential (including neutrophils, basophiles, eosinophils, monocytes and

lymphocytes) and platelet count will be measured when appropriate. Complete hematology will be done at screening, at inclusion, at 24 hours and days 2, 7, 14, 21, 28 and 84.

Clinical Chemistry

Glucose will be measured only at screening. Alanine aminotransferase (ALT), aspartate aminotransferase (AST), creatinine, will be measured at screening, inclusion, at 24 hours and days 2, 7, 14, 21, 28 and 84.

8.3.12 Urine Toxicology analysis

A midstream urine sample (approx. 30ml) will be obtained, in order to avoid contamination with epithelial cells and sediments, to allow proper assessment. A toxicology screening will be performed at screening and at baseline. A positive test for amphetamines and cocaine is a reason for exclusion. A positive test for cannabis on the inclusion day prior to TB31F administration will also be a reason for exclusion.

8.3.13 Pregnancy Test

A midstream urine sample (approx. 30ml) will be obtained and assessed by commercially available hCG urine tests. This test will be done at screening (visit 1) and inclusion (visit 2).

8.3.14 Discontinuation of study procedures

Participants have the right to decline study procedures for any reason and at any time during the study. If a subject declines study procedures, this will be recorded as a study deviation and the reason will be clearly documented in the source document. The subject will be encouraged to complete all remaining safety related follow-up visits and procedures through the entire study period, including unscheduled visits. The reason for all discontinuations will be documented in source documents and the eCRF.

8.3.15 Standard Membrane Feeding Assay

Samples for SMFA will be collected from all subjects. Sera and/or purified IgG prepared from each serum will be used in the SMFA. Samples will be frozen until SMFA is planned. SMFA assays will be performed at both Radboudumc (The Netherlands) and the Laboratory of Malaria and Vector Research (LMVR), NIH USA, using a qualified standard membrane feeding assays using serum from subjects in cultures with *P. falciparum* and *Anopheles stephensi* mosquitoes.

8.3.16 Pharmacokinetic laboratory assay

A qualified ELISA assay developed at the Human Immunology Laboratory of Imperial College London will be used to measure the pharmacokinetics of TB31F in subjects' sera. The ELISA will detect TB31F in sera against its target antigenic epitope (R0.6C) at 12 time points as shown in section 8.3.18.

8.3.17 Safety Assessments

Planned safety assessments will provide the data for active monitoring of investigational product safety during conduct of the trial and for the primary reactogenicity and safety endpoints. Subjects will be observed directly for 60 minutes following product administration. AEs will be recorded prior to infusion, EOI, 6 and 24 hours, and at all follow-up visits. Clinical laboratory results assessed as AEs will be scored for severity using adapted Food and Drug Administration Toxicity Tables (see Appendix 1).

8.3.18 Time and Event procedures: TB31F

Type of contact	Visit 1 (Screening)	Visit 2	Visit 3					Visit 4	Visit 5	Visit 5a*	Visit 6	Visit 6a*	Visit 7	Visit 8	Visit 9	Visit 10	Visit 11
**Study Day**	**D-84 to D-1**	**D-1**	**D0**	**D0**	**D0**	**D0**	**D0**	**D1**	**D2**	**D4**	**D7**	**D10**	**D14**	**D21**	**D28**	**D56**	**D84**
**Time after D0 infusion**			**pre**	**EOI**	**1hr**	**3hr**	**6hr**	**24hr**	**48hr**	**96hr**	**Wk1**	**Wk1,5**	**Wk2**	**Wk3**	**Wk4**	**Wk8**	**Wk12**
Informed consent	●																
Inclusion/exclusion criteria	●	●															
Demographic data and medical history	●																
Physical examination[1]	●[1]	●[1]	●[1]	●[1]	●[1]	●[1]	●[1]	●[1]	●[1]	●[1]	●[1]	●[1]	●[1]	●[1]	●[1]	●[1]	●[1]
Height and weight measurement	●[1]	●[1]															
Blood pressure, temperature, pulse[2]	●	●[2]	●[2]	●[2]	●[2]	●[2]	●[2]	●[2]	●[2]	●[2]	●[2]	●[2]	●[2]	●[2]	●[2]	●[2]	●[2]
Record any concomitant medications	●	●	●					●	●	●	●	●	●	●	●	●	●
HIV, HBV, HCV (3,5mL)	●																
Pregnancy test (urine)	●	●															
Drugs screening (urine)	●	●															
Distribution of emergency notification card		●															
Distribution of memory aid card		●															
COVID-19 diagnostics[3]		●															
TB31F administration			●														
Solicited local AEs (days 0–7)			●	●			●	●	●		●						
Solicited general AEs (days 0-28)			●	●			●	●	●	●*	●	●*	●	●	●		
Unsolicited AEs (days 0-84)			●	●			●	●	●	●*	●	●*	●	●	●	●	●
Complete blood count[4] (2mL)	●	●						●	●		●		●	●	●		●
Biochemistry tests[5] (4mL)	●	●						●	●		●		●	●	●		●
PK samples (CAT tube)[6]			●	●	●	●	●	●	●	●*	●	●*	●	●	●	●	●
ADA samples (CAT tube)[6]			●								●		●		●	●	
SMFA (CAT tube)[6,7]			●	●				●	●	●*	●	●*	●	●	●	●	●

**Type of contact**	**Visit 1 (Screening)**	**Visit 2**	**Visit 3**					**Visit 4**	**Visit 5**	**Visit 5a***	**Visit 6**	**Visit 6a***	**Visit 7**	**Visit 8**	**Visit 9**	**Visit 10**	**Visit 11**
**Study Day**	**D-84 to D-1**	**D-1**	**D0**	**D0**	**D0**	**D0**	**D0**	**D1**	**D2**	**D4**	**D7**	**D10**	**D14**	**D21**	**D28**	**D56**	**D84**
**Time after D0 infusion**			**pre**	**EOI**	**1hr**	**3hr**	**6hr**	**24hr**	**48hr**	**96hr**	**Wk1**	**Wk1,5**	**Wk2**	**Wk3**	**Wk4**	**Wk8**	**Wk12**
Safety Report[8]											•						•
End of study visit																	•
Daily blood volume (mL)	13,5	6	34	24	10	10	10	30	30	24*	40	24*	40	30	40	34	30
Cumulative blood volume (mL)	13,5	19,5	53,5	77,5	87,5	97,5	107,5	137,5	167,5		207,5		247,5	277,5	317,5	351,5	381,5*

1. A physical examination including height and weight and vital parameters will be performed at the screening visit. At inclusion, a repeat measurement of body weight will be obtained. Additional measurements will take place at the discretion of the physician.
2. Vital signs including body temperature, blood pressure and pulse will be recorded at the screening visit. Additional measurements will take place at the discretion of the physician.
3. E.g. nasopharynx/throat swab for SARS-CoV-2 PCR, where required by current Radboudumc-wide or MC-specific guidelines. Symptom-driven diagnostics may also be performed at other visits.
4. CBC test includes: hemoglobin, hematocrit, platelets, red blood cell count, MCV, MCH, MCHC, white blood cell count + differentiation
5. Biochemistry test includes: creatinine, urea, sodium, potassium, bilirubin, AF, yGT, AST, ALT, LDH and hs-Trop-T. Blood will be collected in 2 lithium heparin tubes (2mL). Additional at screening: serology malaria (2mL), cholesterol, triglyceride and glucose (2mL).
6. Serum will be collected in 4mL or 10mL CAT tubes.
7. SMFA assays will be performed at 6 time points (Pre, EOI, D7, D28, D56, D84). Serum will be collected for optional/exploratory SMFA sampling time points on D1, D2, D4*, D10*, D14 and D21.
8. Safety report: A safety report for each group is prepared by the clinical investigator once the final subject in that group has completed 7 days of follow-up. A safety report of all the safety data is made after the close-out visit.

* Visit 5a and 6a will only be performed in group 5. The cumulative blood volume in group 5 is approximately 430 mL.

8.4 Withdrawal of individual subjects

Subjects can leave the study at any time for any reason if they wish to do so without any penalty or loss of medical benefits. Subjects can also be withdrawn from the study procedures at the discretion of the clinical investigator or the local safety monitor for urgent medical reasons or if exclusion criteria are met. The following reasons may lead to withdrawal of individual subjects:

- Withdrawal of informed consent by volunteer;
- Any serious adverse event;
- Any adverse event that, according to clinical judgment of the investigator, is considered as a definite contraindication to proceeding with the study procedures;
- Immunosuppressant or other immune-modifying drugs administered chronically (i.e., more than 14 days in total) during the study period. Topical steroids are allowed;
- Immunoglobulin and/or any blood products administered during the study period;
- Pregnancy;
- Completely lost to follow-up;
- Ineligibility (arising during the study or retrospectively, having been overlooked at screening);
- If the investigator or safety monitor believes that continuation would be detrimental to the subject's well-being;
- Volunteer non-compliance with study requirements;
- Any other protocol deviation that results in a significant risk to the subject's safety.

8.4.1 Handling of withdrawals

For subjects lost to follow-up (i.e. those subjects whose status is unclear because they fail to appear for study visits without stating an intention to withdraw), extensive effort (i.e. documented phone calls and e-mails) will be undertaken to locate or recall the volunteer or at least to determine his or her health status. The investigator should show "due diligence" by documenting in the source documents steps taken to contact the subject. In case of premature withdrawal for any reason, the investigator will exert his/her best effort to:

- Update any ongoing AE/SAEs that remained ongoing at the time of the subject's last visit prior to withdrawal.
- Conduct an interview to determine if the subject has had any reaction or AE (serious or non- serious) since the last visit. Where possible, the investigator should visibly or physically assess any reported adverse reaction or AE and document whether it led to the withdrawal.
- Conduct a physical examination.
- Collect blood for biochemical and hematological clinical laboratory parameters.
- Review the subject's memory aid if it is still in use at the time of withdrawal.
- Document the reason for premature withdrawal on the CRF.
- Update the subject's contact information.

8.4.2 Withdrawals at specific time-points

<u>During screening</u>

If the investigator ascertains that the subject does not meet the inclusion/exclusion criteria after the subject signs the informed consent form, the investigator only needs to note the reason for subject exclusion on the screening log.

<u>As a result of erroneous inclusion</u>

If a subject who does not meet the inclusion/exclusion criteria is inadvertently included in the trial, the investigator will terminate the subject's participation in the trial, the sponsor will be informed immediately prior to administration of study product and a protocol deviation will be completed. In the event that the study product has already been given, the subject will be encouraged to complete all remaining safety related follow-up visits and procedures through the last study visit. However, the subject's data will be excluded from the analysis.

<u>As a result of withdrawal of informed consent</u>

If a subject withdrawal occurs for any reason, the investigator must make every effort to determine the primary reason for a subject's withdrawal from the study and record this information in the study file. However, in accordance with the principles of the current version of the Declaration of Helsinki, a subject does have the right to withdraw from the study at any time and for any reason and is not obliged to give his or her reasons for doing so.

With permission of the subject, all data generated before withdrawal will be included in final study analysis with subject's permission, unless it is felt that the data is compromised. Blood samples collected before withdrawal will be used/stored unless the subject specifically requests otherwise.

<u>As a result of occurrence of an adverse event/serious adverse event</u>

If any subject develops an AE/SAE leading to premature withdrawal, the event will be fully followed-up. For premature withdrawal in connection with the emergence of an AE/SAE that is considered to be clinically relatively favorable (for example, the diagnosis is known, and it is expected to be resolved completely within a week), intermittent follow-up may be accepted as long as the plan for follow-up of the event is fully described in the notes section of the subject's CRF. Follow-up visits will be carried out for all subjects who are withdrawn due to occurrence of an AE or in connection with a change in any other safety indicator (vital sign or clinical laboratory result).

All data generated before withdrawal will be included in final study analysis with subject's permission, unless it is felt that the data is compromised. Blood samples collected before withdrawal will be used/stored unless the subject specifically requests otherwise.

<u>As a result of lost to follow-up</u>

To prevent lost to follow-up, subjects will be reminded by phone, email, or text message of their next study visit. In the event of a missed visit, subject will be contacted by phone within 1 day. A

subject who misses two consecutive visits and cannot be reached or located after 5 attempts will be considered lost to follow-up. Efforts to contact the subject will be documented in source documents. Any subject who fails to attend the final study visit will also be classified as lost to follow-up. There will be no replacement for subjects who are lost to follow-up. All data generated before withdrawal will be included in final study analysis with subject's permission, unless it is felt that the data is compromised. Blood samples collected before withdrawal will be used/stored unless the subject specifically requests otherwise.

8.5 Replacement of individual subjects after withdrawal

If an assigned subject does not present on the day of mAb TB31F study product administration or elects to withdraw the consent on the day of study product administration, one of the reserve subjects will replace the subject. After subjects have received administration with mAb TB31F they cannot be replaced.

8.6 Follow-up of subjects withdrawn from treatment

A subject may end his or her participation in the study and still be followed up for safety, unless the subject chooses to have complete withdrawal of the consent for further participation in any trial procedures. If a subject chooses to withdraw from the study, the investigator will make a reasonable effort to determine the reason for the subject's withdrawal and complete the study termination eCRF.

8.7 Premature termination of the study

The study may be discontinued by the sponsor:

- On advice of the safety monitor
- On advice of the Safety Monitoring Committee (SMC)
- On advice of the clinical investigator
- On advice of the METC

The investigators, local safety monitor, SMC, METC or Sponsor may decide to put the study on hold based on adverse events, pending discussion with the Sponsor, SMC, METC, local safety monitor or investigators. Following discussion, it may be decided to terminate the study.

9. SAFETY ASSESSMENT AND REPORTING

9.1 Temporary halt for reasons of subject safety

In accordance to section 10, subsection 4, of the WMO, the sponsor will suspend the study if there is sufficient ground that continuation of the study will jeopardize subject health or safety. The sponsor will notify the METC without undue delay of a temporary halt including the reason for such an action. The investigator will take care that all subjects are kept informed. PATH REC will also be notified of any decision to prematurely suspend or terminate the study. Safety monitoring will be conducted throughout the study.

9.2 AEs, SAEs and SUSARs

9.2.1 Adverse events (AEs)

Adverse events are defined as any undesirable experience occurring to a subject during the study, whether or not considered related to the investigational product. All adverse events reported spontaneously by the subject or observed by the investigator or his staff will be recorded. Abnormal laboratory findings (e.g. clinical chemistry or hematology) or other abnormal assessments that are judged by the investigator to be clinically significant will be recorded as AEs (or SAEs if they meet the definition). The investigator will exercise his or her medical and scientific judgment in deciding whether an abnormal laboratory finding or any other abnormal assessment is clinically significant. If there are any severe complaints, the volunteer will be evaluated immediately by a qualified clinician using the appropriate clinical assessments according to standard hospital care.

9.2.2 Clinical Laboratory Parameters

To the extent possible, all normal ranges for clinical laboratory test results will be pre-specified in site reference range documents, but the investigator will exercise his/her medical and scientific judgment in deciding whether an abnormal laboratory finding or other abnormal assessment is clinically significant. Clinically significant abnormal laboratory findings that present at baseline and significantly worsen following the start of the study will also be reported as AEs or SAEs.

9.2.3 Serious adverse events (SAEs)

A serious adverse event is any untoward medical occurrence or effect that:

- results in death;
- is life threatening (at the time of the event);
- requires hospitalization or prolongation of existing inpatients’ hospitalization;
- results in persistent or significant disability or incapacity;
- is a congenital anomaly or birth defect; or

- any other important medical event that did not result in any of the outcomes listed above due to medical or surgical intervention but could have been based upon appropriate judgment by the investigator.

An elective hospital admission will not be considered as a serious adverse event. The investigator will report all SAEs to the sponsor and the safety monitor without undue delay after obtaining knowledge of the events (within 24 hours). All SAEs will be reported through the web portal *ToetsingOnline* to the accredited METC that approved the protocol, within 7 days for SAEs that result in death or are life threatening followed by a period of maximum of 8 days to complete the initial preliminary report by the investigator/sponsor. All other SAEs will be reported within a period of maximum 15 days after the sponsor has first knowledge of the serious adverse event. Death is an outcome of an event. The event that resulted in the death should be recorded and reported on the SAE CRF.

9.2.4 Suspected unexpected serious adverse reactions (SUSARs)

Adverse reactions are all untoward and unintended responses to an investigational product related to any dose administered. Unexpected adverse reactions are SUSARs if the following three conditions are met:

1. the event must be serious (see chapter 9.2.3);
2. there must be a certain degree of probability that the event is a harmful and an undesirable reaction to the medicinal product under investigation, regardless of the administered dose;
3. the adverse reaction must be unexpected, that is to say, the nature and severity of the adverse reaction are not in agreement with the product information as recorded in:
   - Summary of Product Characteristics (SPC) for an authorized medicinal product;
   - Investigator's Brochure for an unauthorized medicinal product.

The sponsor will report expedited the following SUSARs through the web portal *ToetsingOnline* to the competent authority:

- SUSARs that have arisen in the clinical trial that was assessed by the METC.
- SUSARs that have arisen in other clinical trials of the same sponsor and with the same medicinal product, and that could have consequences for the safety of the subjects involved in the clinical trial that was assessed by the METC.

The remaining SUSARs are recorded in an overview list (line-listing) that will be submitted once every half year to the METC. This line-listing provides an overview of all SUSARs from the study medicine, accompanied by a brief report highlighting the main points of concern.

The expedited reporting of SUSARs through the web portal Eudravigilance or ToetsingOnline is sufficient as notification to the competent authority. The sponsor will report expedited all SUSARs

to the competent authorities in other Member States, according to the requirements of the Member States. The expedited reporting will occur not later than 15 days after the sponsor has first knowledge of the adverse reactions. For fatal or life threatening cases the term will be maximal 7 days for a preliminary report with another 8 days for completion of the report. As this is an open label study in which the sponsor, investigator and the SMC are not blinded, the code would not have to be broken in the case of a SUSAR.

9.3 Annual safety report

In addition to the expedited reporting of SUSARs, the sponsor will submit, once a year throughout the clinical trial, a safety report to the accredited METC, competent authority, and competent authorities of the concerned Member States. This safety report consists of:

- a list of all suspected (unexpected or expected) serious adverse reactions, along with an aggregated summary table of all reported serious adverse reactions, ordered by organ system, per study;
- a report concerning the safety of the subjects, consisting of a complete safety analysis and an evaluation of the balance between the efficacy and the harmfulness of the medicine under investigation.

9.4 Follow-up of adverse events

All AEs will be followed until they have abated, or until a stable situation has been reached. Depending on the event, follow up may require additional tests or medical procedures as indicated, and/or referral to the general physician or a medical specialist. SAEs need to be reported until end of study within the Netherlands, as defined in the protocol. After termination of the trial, the investigator should assure that the subject is referred for medical follow-up, as appropriate.

9.4.1 **Adverse event data collection**

Signs and symptoms will be recorded in study diaries and reviewed during admission at the clinical trial research center, at all follow-up visits, and whenever a trial volunteer reports signs or symptoms to the trial physician between visits.

Solicited adverse events

After administration with mAb TB31F the following systemic signs and symptoms will be solicited until 28 days after infusion:

Fever, headache, myalgia, fatigue, chills, and rash. The time points that these symptoms are solicited is indicated in section 8.3.18.

After the administration of mAb TB31F the following local signs and symptoms will be solicited until 7 days after administration:

Pain at injection site and redness/swelling at injection site. The time points that these symptoms are solicited is indicated in section 8.3.18.

9.4.2 Monitoring periods for safety events

Adverse events will be collected during the study during the following periods:

- Solicited local AEs commonly associated with monoclonal antibody administration occurring through 7 days (day of TB31F administration and subsequent 6 days);

- Solicited general AEs commonly associated with monoclonal antibody administration and clinically significant hematological and biochemical laboratory abnormalities occurring through 28 days (day of TB31F administration and subsequent 27 days);

- Unsolicited AEs occurring through 84 days following TB31F administration (day of TB31F administration and subsequent 83 days);

- AEs (including SAEs) leading to withdrawal from the study during the entire study period post- mAb TB31F administration;

- SAEs during the entire study period post mAb TB31F administration;

9.4.3 Recording of adverse event data collection

Study staff will train subjects on use of memory aid booklets for self-assessment of solicited local and general adverse events through 7 days (day of mAb TB31F administration and subsequent 6 days) post- administration of study mAb TB31F; and for self-assessment of adverse events and concomitant medications until day 28. Study staff will review and discuss with the subject all recorded events to ensure appropriate documentation and scoring prior to transferring of the diary card record to the eCRF.

If known, the trial clinician will record the diagnosis (i.e., disease or syndrome) rather than component signs, symptoms and laboratory values. If the signs and symptoms are considered unrelated to an encountered syndrome or disease they should be recorded as individual AEs. If a primary AE is recorded, events occurring secondary to the primary event should be described in the narrative description of the case (e.g. primary AE = Orthostatic hypotension; secondary event may be fainting, head trauma, etc.). In case of hospitalizations for surgical or diagnostic procedures, the pre-existing condition should be recorded as the SAE, not the procedure itself.

All adverse events/reactions (solicited and unsolicited), noted by the investigators will be accurately documented in the case report form by the investigators. For each event/reaction the following details will be recorded:

1. Description of the event(s)/reactions(s)
2. Date and time of occurrence
3. Duration
4. Intensity
5. Relationship with the intervention
6. Action taken, including treatment
7. Outcome

In addition, symptoms will be ranked as (1) mild, (2) moderate, (3) or severe, depending on their intensity according to the following scale:

Mild (grade 1):	awareness of symptoms that are easily tolerated and do not interfere with usual daily activity
Moderate (grade 2):	discomfort that interferes with or limits usual daily activity
Severe (grade 3):	disabling, with subsequent inability to perform usual daily activity, resulting in absence or required bed rest

If an adverse event changes in intensity during the specified reporting period, a new description of the adverse event will be added. Interrupted AEs are registered as one AE if the interruption is <24 hours. When an AE/SAE occurs, it is the responsibility of the investigators to review all documentation (e.g. hospital progress notes, laboratory, and diagnostics reports) related to the event. The investigators will then record all relevant information regarding an AE/SAE on the CRF or SAE Report Form, respectively.

The investigator will attempt to establish a diagnosis of the event based on signs, symptoms and/or other clinical information. In such cases, the diagnosis should be documented as the AE/SAE and not the individual signs/symptoms.

9.4.4 Assessment of causality

The investigators are obliged to assess the relationship between study procedures and the occurrence of each AE/SAE. The investigators will use clinical judgment to determine the relationship. Alternative causes, such as natural history or the underlying diseases, concomitant therapy, other risk factors and the temporal relationship of the event will be considered and investigated. The relationship of the adverse event with the study procedures will be categorized as:

Definitely:	administration of the IP is the cause, another etiology causing the adverse event is not known.
Probable:	administration of the IP is the most likely cause: however, there are alternative reasonable explanations, even though less likely.
Possible:	there is a potential association between the event and administration of the IP, however, there is an alternative etiology that is more likely.
Unlikely:	a relationship to the administration of IP is unlikely, however, it cannot be ruled out.

Not related: a relationship to the administration of the IP cannot be reasonably established; another etiology is known to have caused the adverse event or is highly likely to have caused is.

When a regulatory authority requests a binary classification (related vs. unrelated), definitely, probably and possibly related are considered to be “related”, while not related and unlikely related are considered to be “unrelated”. Thus, an intervention-related AE refers to an AE for which there is a possible, probable or definite relationship to the study intervention. The investigator will use clinical judgment to determine the relationship.

9.4.5 Follow-up of adverse events

All AEs will be followed until they have abated, or until a stable situation has been reached. AEs that result in a subject’s withdrawal from the study or that are present at the end of the study will be followed up (if the volunteer consents to this) until a satisfactory resolution or stabilization occurs, or until a non-study related causality is assigned. Depending on the event, follow-up may require additional tests or medical procedures as indicated and/or referral to the general physician or a medical specialist. AEs and SAEs will be reported until end of study within the Netherlands, defined as the last patient visit.

9.5 Safety Monitoring Committee

9.5.1 Local safety monitor

For this study, a local safety monitor will be appointed, who will be involved in the review of severe and serious adverse events and volunteer safety. He/she is independent of the sponsor and the investigator. The local safety monitor is notified of all grade III adverse events probably or definitely related to TB31F administration and persisting at grade 3 for >48 hours.

9.5.2 Safety monitoring committee (SMC)

An independent SMC composed of three independent individuals will be appointed. The SMC will include an independent local safety monitor, and two independent experts nominated by the PI. A PATH representative is allowed as an observer on the SMC, and is promptly informed of any safety concerns that have been raised by the SMC or have been raised to the SMC for their advice or review, and of their response.

The SMC will be established for the purpose of monitoring the study and to provide independent, non-binding advice on safety and ethics. The responsibilities and procedures of the SMC members are defined in the SMC Charter.

9.5.3 Review of safety data by the safety monitor and SMC

Safety Meetings

The SMC will review safety data at pre-defined time points throughout the study, specifically: upon completion of ≥7 days of post-administration follow-up in all subjects in a given dose-group (prior to commencing the first administration in the subsequent dose group). The chair of the SMC will determine for each time point whether a meeting will be held, or whether a recommendation from all members may be formalized through e-mail. The frequency of these reviews may be adapted upon SMC recommendation if deemed necessary. In addition, safety data for all participants will be assessed by the SMC at the end of the study. An ad hoc SMC meeting may be convened at any time or at the request of the PI or local safety monitor to review safety data from subjects and/or groups who meet any of the holding rules as specified in the protocol (9.5.4) and SMC charter.

Safety Reports

Safety reports will be prepared by the clinical investigator for review by the committee prior to each review. These reports will provide at a minimum the following information:

- Accrual data and subject status data with regard to completion of/discontinuation from the study.
- Summaries of solicited AEs, classified by severity.
- Unsolicited AEs (including SAEs), categorized by ICD-10 coding, severity and relatedness to study vaccine.
- Safety laboratory test results outside of normal institution reference ranges and considered clinically significant, classified by severity grading scale (irrespective of whether assessed as AEs).
- Any new or updated AEs that have met the holding rules.

The SMC will review the safety data within 2 working days. The SMC will summarize their recommendations to the study Sponsor as to whether there are safety concerns and whether the study should continue without change, be modified, or be terminated. If at any time a decision is made to permanently discontinue administration of study TB31F administrations in all subjects, the Sponsor will notify the METC and PATH expeditiously.

The advice(s) of the SMC will only be sent to the Sponsor of the study. Should the Sponsor decide not to fully implement the advice of the SMC, the Sponsor will send the SMC advice to the METC, including a note to substantiate why (part of) the advice of the SMC will not be followed.

Within-group progression

Subjects within a dosage group are to receive a single TB31F administration following a schedule, such that the first subject (sentinel) in each group will be administered mAb TB31F and observed for the occurrence of any AEs. The 2nd subject in each dose group will receive their dose of TB31F no sooner than 2 days after the first subject has received TB31F. For group 1, each remaining subject will receive their TB31F dose no sooner than 2 days after the previous subject. For groups 2-5, the remaining three

subjects in each dosage group will receive their TB31F dose no sooner than 2 days after the second subject has been administered TB31F. For the three remaining subjects in each dosage group, there will be at least a one hour interval in administration of TB31F between the completion of TB31F administration in one subject before the next subject is administered TB31F. On condition no holding rules are met (see below), administration of TB31F to all subjects within each dosage group will continue without any external (SMC) review. If a holding rule is met, administration of TB31F drug product will be held for all remaining subjects in that dosage group until the SMC has completed its review and made a recommendation on trial progress.

Inter-group progression

Progression to the next dosage group will depend in all cases upon a positive review by the SMC of safety data of the previous dosage group. The data to be reviewed before each dose group includes the safety data (solicited and unsolicited AEs through day 7 post TB31F administration) and clinically significant laboratory tests collected at 1, 2 and 7 days following TB31F administration for all subjects in the specified dose group. The SMC will review the AEs (local, general, and laboratory) and will make recommendations regarding the continuation of the study for each group.

9.5.4 Safety holding rules

The study may be placed on safety hold at any time for the following reasons:

- On advice of the safety monitor;
- On advice of the Principal/Clinical investigators;
- On advice of the SMC;
- On advice of the accredited METC;
- If holding rules are met (see below).

Holding rules for within-group progression:

If either of the first two subjects within a group meet a holding rule (see below), administration of TB31F will be held for all remaining subjects in that dosage group (and subsequent groups). The following stopping rules apply to subjects within a group:

- One or more participants experience a SAE that is determined to be at least possibly related to the administration of TB31F
- Two or more subjects experience a grade 3 adverse event (local, clinical systemic or laboratory systemic) possibly, probably or definitely related to TB31F administration and persisting at grade 3 for >48 hours

The study site member first aware of the event meeting the holding rule will notify the Principal Investigator and the Local Safety Monitor. The PI will alert the appropriate parties. The SMC will be notified within 24 hours. An ad hoc SMC review will be performed. The following considerations must be discussed:

- Relationship of the AE or SAE to the study product
- Relationship of the AE or SAE to TB31F dose
- If appropriate, additional screening or laboratory testing is provided to other subjects to identify subjects who may develop similar symptoms
- If any study related SAE is not listed on the current informed consent form (ICF), the PI will revise the ICF and subjects will be asked to provide consent on the new ICF

TB31F administration to subjects within the affected group and to the next higher dosage group may resume only if the local safety monitor, PI, SMC and the sponsor agree it is safe to resume TB31F administration. If the accredited METC has recommended safety hold, re-initiation of the study will require METC concurrence. The accredited METC and PATH REC will be informed of a safety hold by the sponsor. Following discussion, it may be decided to terminate the study.

All subjects who have received the study product will be followed for safety until resolution or stabilization (if determined to be chronic sequelae) of their AE(s). If at any time a decision is made to discontinue administration of study product in all subjects, expeditious notification will be provided by the Sponsor to the METC within 48 hours. The PI, local safety monitor, SMC, METC or PATH may stop or suspend the use of this product at any time.

10. STATISTICAL ANALYSIS

All data will be collected and verified prior to analysis. All data will be analyzed in accordance with the Statistical Analysis Plan. Final analyses of all data will occur after study completion and final verification of data according to GCP. All data analyses will be conducted using IBM SPSS and R using the latest version available. Detailed statistical procedures, listings, table shells, and figures will be provided in a SAP prior to analysis. The SAP will be finalized before study close-out and database lock. The following key statistical components will be considered and a detailed description will be documented in the SAP:

- Primary and secondary endpoints and how they will be measured
- Statistical methods and tests that will be used to analyze the endpoints
- Strategy that will be used if the statistical test assumptions are not satisfied
- Indication of whether the comparisons will be using one-tailed or two-tailed *t*-test (with justification of the choice) and the level of significance to be used
- Identification of whether any adjustments to the significance level or the overall P-value will be made to account for any planned or unplanned subgroup analyses or multiple testing

- Specification of potential adjusted analyses and a statement with which covariates or factors will be included
- Planned exploratory analyses and justification of their importance
- Any subgroup effects with biological justification and support from within and outside the study

10.1 Primary study parameter(s)

The number of subjects enrolled, completed, or withdrawn will be summarized. Reasons for withdrawal, when known, will be provided. Demographic data will be summarized by descriptive statistics and will include total number of observations (n), mean, standard deviation (SD) and range for continuous variables, and number and percentages for dichotomous variables. The primary safety, and reactogenicity outcomes will include all subjects who meet the eligibility criteria, receive study product mAb TB31F, and for whom safety data are available.

Safety Analyses

For the safety analysis, data from all subjects who received a dose of mAb TB31F and for whom safety data are available will be included. All analyses will be descriptive. Data will be presented by dose, overall/dose and overall/subject. Results will be summarized by study group. The percentage of subjects with at least one local AE (solicited and unsolicited), with at least one general AE (solicited and unsolicited) and with any AE during the solicited follow-up period will be tabulated with exact 95% CI (two-sided). No multiplicity adjustment will be implemented in analysis. The same calculations will be performed for AEs rated as grade 3. The percentage of subjects reporting each individual solicited local and general AE during the solicited follow-up period will be tabulated with exact 95% CI. The same tabulation will be performed for grade 3 AEs and for AEs with relationship to mAb TB31F administration. The reports of unsolicited AEs will be reviewed by a physician and will be categorized by ICD-10. The percentage of subjects with at least one report of unsolicited AE and reported up to 28 days after mAb TB31F administration will be tabulated with exact 95% CI. The same tabulation will be performed for grade 3 unsolicited AEs and for unsolicited AEs with a relationship to mAb TB31F administration. SAEs will be described in detail. Withdrawals due to AEs/SAEs will also be summarized. Vital signs which are outside of the normal range and clinically significant will also be listed in tables. The frequency of signs and symptoms will be compared between groups with the chi- square test or fishers exact test. Serious adverse events (SAEs) occurring at any point during the trial will be summarized and relatedness to vaccine will be assessed.

Clinical Laboratory Data Analyses

Any clinically important deviations in routine laboratory test results and/or vital signs as determined by the investigator will be listed. Isolated laboratory abnormalities will be reported as AEs if they are considered clinically relevant by the investigator. Vital signs which are considered clinically

relevant by the investigator will be summarized. All adverse events will be listed by participant and will include details of onset time, duration, severity and relationship to the study product.

10.2 Secondary study parameter(s)

Analysis of Pharmacokinetic Endpoints

MAb TB31F pharmacokinetics will be analyzed using standard non-compartmental methods using the Phoenix WinNonlin software package. The time-concentration and dosing data from all subjects who received mAb TB31F will be used to establish the half-life ($t_{1/2}$), area under the curve ($AUC_{0-\infty}$), drug clearance (CL), and Volume of Distribution (Vd). Results will be reported as geometric means and geometric coefficients of variation for all dose groups.

SMFA will be performed to determine TRA of mAb TB31F. Differences will be assessed by comparing mean values between the groups or time points using either a two-tailed student's t-test (if comparing two groups) or a one-way ANOVA (if comparing more than two groups) or non-parametric equivalents. Paired tests will be used if pre-intervention values are compared with post-intervention values, unpaired if comparisons are made between groups. For discrete variables (e.g. the number of positive assays), the chi-squared test or Fisher's exact test will be used (two-tailed).

The relationship between plasma mAb TB31F concentrations and transmission reducing activity in SMFA will be assessed using standard l empirical pk-pd models using Graphpad Prism v8.[23]

10.3 Other study parameters

Analysis of Immunogenicity Endpoints

Analysis of immunogenicity for emergence of anti-idiotypic antibodies against the study product, mAb TB31F will be measured in terms of seropositivity rates and geometric mean titers (GMTs) with exact 95% CI in samples collected at the same time points as samples drawn for pK measurements.

Non-linear mixed effects modeling

The obtained pharmacokinetic pharmacodynamic data will be further analyzed by means of non-linear mixed effects modeling according to best practice [23, 24] by the Radboud Applied Pharmacometrics group. Using an exploratory approach, we will fit single and multicompartmental methods to the obtained pharmacokinetic data and investigate both linear and non-linear elimination and disposition. Thereafter, we will investigate whether the pharmacokinetics relate with inhibition of plasmodium transmission, as assessed with the membrane feeding assay, using standard empirical pk-pd models. The developed model will be used for exploratory *in silico* analysis of the pharmacokinetics and pharmacodynamics in various dosing regimens by performing Monte Carlo simulations.

10.4 Interim analysis

There are no statistical criteria for study termination in this clinical trial. Safety and reactogenicity data will be evaluated after each mAb TB31F administration before proceeding to the next group. A summary report of AEs will be provided to the SMC after all four groups have completed follow-up.

10.5 Accounting for Missing, Unused, and Spurious Data

The SAP will be followed even if data are missing. For the safety analysis, data from all subjects who received a dose of mAb TB31F in the study will be included. Subjects with missing data will not be imputed. Subjects who miss an appointment date will not be removed from the study. Rather, their appointment and laboratory values will be recorded by appropriate missing value notation in the clinical database. Non-analyzable data will be documented in the deviations.

11. ETHICAL CONSIDERATIONS

11.1 Regulation statement

This study will be conducted in accordance with the latest Fortaleza revision of the Declaration of Helsinki (2013), the Medical research Involving Human Subjects act (WMO), the ICH Good Clinical Practice, and local regulatory requirements. The investigators are responsible for obtaining all relevant ethical approvals of the protocol and any subsequent amendments in compliance with local law before the start of the study.

11.2 Recruitment and consent

As soon as the study is approved by the accredited METC and competent authority, healthy subjects will be recruited to participate in the study. Advertisements will be placed in prominent places on university campuses and other public places as well as on the intranet of the University and social media. Furthermore, a Facebook page (link: http://www.facebook.com/malariavaccin) showing the advertisement text will be used to inform people about the trial. This brief advertisement will indicate a telephone number to call and an e-mail address for contact to request further information. It will furthermore indicate a website (www.malariavaccin.nl) which contains a form. This short questionnaire will be completed using the online form. When seemingly suitable subjects contact investigators via e-mail, telephone or the online form, they will be invited to an information meeting during which the study will be explained to them by the study investigator. Directly after the meeting they will be provided with documents to review at home (the information sheet, the informed consent form, the application form and the insurance text). During and after the meeting there will be time for questions. After this free discussion with the investigator, and any follow-up discussion if necessary, the volunteer will be given sufficient time to consider participation.

Subjects who are interested in participating will be asked to fill in the application form and will be invited to come for a screening visit. Eligible subjects may only be included in the study after providing written, METC- approved informed consent. Informed consent must be obtained before conducting any study-specific procedures (i.e. all of the procedures described in the protocol, including screening procedures). The process of obtaining informed consent should be documented in the subject source documents. During the screening visit, the questionnaire answers will be discussed and inclusion and exclusion criteria will be checked. In addition, a letter for the general practitioner will be signed and sent after screening. Again, the investigator will answer any questions the volunteer has. The possibility of withdrawal from the study, at any time, without penalty and without any declaration of the reason will be pointed out to the subjects. The investigators will be responsible for providing adequate verbal and written information regarding the objectives and procedures of the study, the potential risks involved and the obligations of the subjects. Subjects will be informed that they will not gain health benefits from this study. Trainees or other students who might be dependent on the investigators or the study group will not be included in the study.

11.3 Benefits and risks assessment, group relatedness

The compelling need for new methods of malaria interventions need to be balanced with the potential risks and discomforts for the subjects. Risks for subjects are related to administration with mAb TB31F. There are no direct benefits to participation in the trial for subjects. Subjects will be advised to take regular malaria chemoprophylaxis when travelling to malaria endemic areas in the future. All partners in this proposal are aware of and follow the relevant national and international rules and regulations as they pertain to access to material of human origin and clinical research. International agreements such as the Declaration of Helsinki will be observed and respected.

11.4 Ethical aspects concerning the use of human subjects

Testing in human subjects remains the only reliable and convincing way to obtain information on the immunological responses that are important for protection against malaria. Of course, the compelling need for a malaria vaccine and treatments needs to be balanced with the potential risks and discomforts of the subjects. Explorative studies looking for new and complementary candidate malaria interventions are of paramount importance with the potential of large-scale application in endemic countries.

The study will be undertaken in accordance with Good Clinical Practices (GCP), according to the standards defined in the EEC directive 91/507/EEC, and in the Directive on Good Clinical Practice in Clinical Trials (ICH GCP, 75/318/EEC, January 1997) and under the principles of the Declaration of Helsinki; ethical permission will be sought from an accredited METC in the Netherlands.

11.5 Compensation for injury

The sponsor/investigator has a liability insurance which is in accordance with article 7 of the WMO. The sponsor (also) has an insurance which is in accordance with the legal requirements in

the Netherlands (Article 7 WMO). This insurance provides cover for damage to research subjects through injury or death caused by the study.

1. € 650.000,-- (i.e. six hundred and fifty thousand euro) for death or injury for each subject who participates in the research;
2. € 5.000.000,-- (i.e. five million euro) for death or injury for all subjects who participate in the research;
3. € 7.500.000,-- (i.e. seven million five hundred thousand euro) for the total damage incurred by the organisation for all damage disclosed by scientific research for the sponsor as ‘verrichter’ in the meaning of said Act in each year of insurance coverage.

The insurance applies to the damage that becomes apparent during the study or within 4 years after the end of the study.

11.6 Compensation

Enrolled subjects will receive up 850,- euros (groups 1-4) or 900,- euros (group 5) in compensation for their time and for the inconveniences of taking part in this study. These amounts are based on predefined criteria:

- Screening and inclusion: 100,- euros
- Inconvenience of blood tests and/or visits: 25,- euros per blood sampling
- 1 day admission to hospital: 125,- euros
- MAb TB31F administration: 300,- euros
- Compensation length of study: 20,- euros per month (3 months)

Travel expenses will not be additionally reimbursed, and compensation will not be provided to subjects who are not enrolled i.e. screen failures. Eligible subjects who are enrolled at the inclusion visit as back-ups, will be compensated 50,- Euros for each inclusion visit. If a subject withdraws from the study prior to completion, they will receive reimbursement proportional to number of visits they attended. These compensation amounts are reasonable and in line with Dutch common practice. In case of unexpected medical complications, there will be access to state-of-the-art medical treatment with full costs covered by the insurance of Radboudumc.

12. ADMINISTRATIVE ASPECTS, MONITORING AND PUBLICATION

12.1 Handling and storage of data and documents

A data management plan will be developed prior to start of study describing data management activities from project set-up through data lock and transfer. Designated trial staff will enter the data required by the protocol into the electronic CRF (eCRF). All data should be recorded, handled and stored in a way that allows its accurate reporting, interpretation and verification. An external monitor will review the data entered into the eCRFs by investigational staff for completeness and accuracy and will instruct the site personnel to make any required corrections or additions. Queries are made during each monitoring visit. Designated investigator site staff are required to respond to the queries and confirm or correct the data. Medical history/current medical conditions and adverse events will be coded using the ICD-10 terminology.

12.1.1 Source data

Prior to the start of the trial it will be determined which documents completed by the investigative team will be considered source documents. Only authorized study staff and representatives of the sponsor and authorized regulatory agencies may have direct access to source documents containing study data. Data for this study will include biographical, medical history, clinical (signs, symptoms, prescription and non-prescription medical treatments), safety and laboratory data. All information in original records and certified copies of original records, clinical findings, or observations will be considered source data for this study. The memory aids, produced by the study subjects are also considered source data. They will be kept as source documents in the investigator's clinical file. Since all subjects will be healthy, there is no medical file for the study subjects, with exception of the medical file in case of adverse events/reactions resulting in a medical consultation or hospitalization. In this case the medical file will also be considered as the source data.

All data collected by the investigators is reported in electronic case report forms. All information on eCRFs must be traceable to the source documents in the subject's file. As with all parts of the eCRF, there is an audit trail in place to register every data entry. The investigator will also keep the original informed consent form signed by the subject (a signed copy is given to the subject). The site PIs/institutions will maintain the information collected from CRFs and in the eCRFs and all source documents that support the data collected from each subject in a secure area and treated as confidential material. Documents and data pertaining to the study will be kept in a locked cabinet under the responsibility of the investigator. Periodic monitoring visits will ensure that the data is safe and stored in this secure place and that only those authorized study staff have access to the data.

Data and biological samples from this study will be stored for 20 years for research related to malaria immunology and transmission dynamics. New immunological or molecular tests may become available in the future that could strengthen or validate this research or help find important new findings. Should material be used for research not related to malaria, permission from the METC

must be granted.

12.1.2 Confidentiality

All parties agree to adhere to the principles of medical confidentiality in relation to clinical study subjects involved in this trial and shall not disclose the identity of subjects to third parties without prior written consent of the subject.
All data will be anonymized; volunteer data will be identified by a unique study number in the CRF. Separate confidential files containing identifiable information will be stored in secured locations. All plasma samples, or other biological samples, with exception of those taken for safety diagnostics, will be labeled with the volunteer study identification number. Samples taken for safety diagnostic (processed by the central clinical laboratory) will be labeled with part of the subject identification code, study identification name and a fictitious birth date (only using the subjects actual birth year). The samples will not be labeled with volunteer names or actual birth dates. The subject identification code will be kept by the principal investigator.
The investigator will maintain and retain appropriate medical and research records and essential documents for this trial in compliance with ICH GCP Guidelines, any regulatory requirements, and institutional requirements for the length of storage and for the protection of confidentiality of subjects. The investigator will permit direct access to study records and source documents to authorized representatives of the sponsor, ethical committee(s) / institutional review board(s), regulatory agencies, authorized individuals from PATH, and the external monitor(s), for the purposes of quality assurance reviews, audits / inspections, and evaluation of the study safety and progress. Direct access includes examination, analysis, verification, and reproduction of de-identified records and reports that are important to the evaluation of the trial.

12.2 Monitoring and Quality Assurance

General considerations

Before study initiation, the protocol and eCRFs together with relevant SOPs will be reviewed by the sponsor, the investigators and their staff. During and after completion of the study, the data monitor will visit the site to check the completeness of records, the accuracy of entries on the eCRFs, the adherence to the protocol and to Good Clinical Practice and the progress of enrolment.

The investigator will maintain source documents for each subject in the study, consisting of case and visit notes containing demographic and medical information, laboratory data, subject’s diaries, and the results of any other tests or assessments. All information on eCRFs must be traceable to these source documents in the subject's file. As with all parts of the eCRF, there is an audit trail in place to register every data entry. The investigator will also keep the original informed consent form signed by the subject (a signed copy is given to the subject).

The investigator will give the external monitor access to all relevant source documents to confirm their consistency with the eCRF entries. According to the NFU risk classification system, this

clinical trial has been classified as high risk. The monitor will perform full verification for the presence of informed consent, adherence to the inclusion/exclusion criteria and documentation of SAEs. The recording of data that will be used for all primary and safety variables will be assessed for 100% of included subjects.

External monitoring

To ensure that the study is conducted in accordance with ICH-GCP and regulatory requirements, monitoring responsibilities will be provided by CRO. A site initiation visit will be conducted prior to beginning of the study, and monitoring will be conducted at initiation, during, and at closeout of the study. During the course of the study, monitors will verify compliance to the protocol; completeness, accuracy, and consistency of the data and study product accountability; and adherence to ICH-GCP and applicable regulations. As needed and when appropriate, the monitors will also provide clarifications and additional training to help the site resolve issues identified during the monitoring visit. As appropriate and informed by risk assessment, remote centralized monitoring activities may be considered in place of or to supplement onsite monitoring. These may include analysis of data quality (e.g., missing or inconsistent data), identification of data trends not easily detected by onsite monitoring, and performance metrics (e.g., screening or withdrawal rates, eligibility violations, timeliness, and accuracy of data submission).

The extent and frequencies of the monitoring visits will be described in a separate monitoring plan developed prior to study initiation. The investigator will be notified in advance of scheduled monitoring visits. The monitors should have access to all trial related sites, subject medical records, study product accountability, and other study-related records needed to conduct monitoring activities. CRO will share the findings of the monitoring visit, including any corrective actions, with the site investigator and PATH. The site PI and the monitors must cooperate to ensure that any problems detected in the course of these monitoring visits are resolved in a predefined timeframe.

To ensure the quality of clinical data for all subjects, a clinical data management review will be performed on subject data received by CRO. During this review, subject data will be checked for consistency, omissions, and any apparent discrepancies. In addition, the data will be reviewed for adherence to the protocol and ICH-GCP. To resolve any questions arising from the clinical data management review process, data queries and/or site notifications will be sent to the site for resolution as soon as possible and within the period described in the monitoring plan; all queries must be resolved prior to database lock.

Essential documents must be filed in the site study file on an ongoing basis and be available for review by CRO.

Independent Auditing

Sponsor representatives may audit the study to ensure that study procedures and data collected comply with the protocol and applicable SOPs at the clinical site and that data are correct and complete. The site PIs will permit auditors (employees of the Sponsor or employee of a company designated by PATH) to verify source data validation of the regularly monitored clinical

study. The auditors will compare the entries in the eCRFs with the source data and evaluate the study site for its adherence to the clinical study protocol and ICH-GCP guidelines and applicable regulatory requirements.

Regulatory agency auditing

The site PIs must be aware that regulatory authorities may wish to inspect the site records to verify the validity and integrity of the study data and protection of human research subjects. The site PIs will notify PATH within 24 hours following contact by a regulatory authority. The site PIs must make the relevant records available for inspection and will be available to respond to reasonable requests and audit queries made by authorized representatives of regulatory agencies. The site PIs will provide PATH with copies of all correspondence that may affect the review of the current study or his qualification as an investigator in clinical studies conducted by PATH. PATH will provide any needed assistance in responding to regulatory audits or correspondence.

12.3 Amendments

Amendments are changes made to the research and will only be made after favorable opinions/approvals by the accredited METC has been given – except where necessary to eliminate apparent immediate hazards to the subject(s).

A 'substantial amendment' is defined as an amendment to the terms of the METC application, or to the protocol or any other supporting documentation, that is likely to affect to a significant degree:

- the safety or physical or mental integrity of the subjects of the trial;
- the scientific value of the trial;
- the conduct or management of the trial; or
- the quality or safety of any intervention used in the trial.

All substantial amendments will be notified to the accredited METC and to the competent authority. Non-substantial amendments (e.g. typing errors and administrative changes like telephone numbers or contact details mentioned in the submitted study documentation) will not be notified to the accredited METC and the competent authority, but will be recorded and filed by the sponsor.

12.4 Annual progress report

The investigator will submit a summary of the progress of the trial to the accredited METC once a year. Information will be provided on the date of inclusion of the first subject, numbers of subjects included and numbers of subjects that have completed the trial, serious adverse events/ serious adverse reactions, other problems, and amendments.

12.5 Temporary halt and (prematurely) end of study report

The sponsor will notify the accredited METC and the competent authority of the end of the study within a period of 90 days. The end of the study is defined as the last patient's last visit. The sponsor will

notify the accredited METC immediately of a temporary halt of the study, including the reason of such an action. In case the study is ended prematurely, the sponsor will notify the accredited METC and the competent authority within 15 days, including the reasons for the premature termination. Within one year after the end of the study, the investigator will submit a final study report with the results of the study, including any publications/abstracts of the study, to the accredited METC and the Competent Authority.

12.6 Public disclosure and publication policy

The study is entered by the sponsor into Clinicaltrials.gov registry and registered under NCT04238689. The final report will be prepared by the investigators at the Radboud university medical center. It will be signed by the project leader or the principal investigator. The investigators will make every effort to publish the results in a peer-reviewed journal.

13. STRUCTURED RISK ANALYSIS

13.1 Potential issues of concern prior to start of study

a. Level of knowledge about mechanism of action

The human monoclonal TB31F antibody is derived from 85RF45.1, a rat monoclonal antibody isolated and characterized at the Radboudumc. MAb 85RF45.1 binds to Pfs48/45 (epitope I), a gametocyte surface protein expressed by gametocytes and early gametes. Pfs48/45 is required for male gametocyte binding to female gametocytes in the mosquito gut. MAb 85RF45.1 shows strong transmission-blocking activity in the SMFA. The functional results of TB31F show TRA of 100% at 10ug/ml and ~80% at ~3 ug/ml. Indirect immunofluorescence against gametocytes is observed at 0.08 ug/ml mAb concentration. TB31F has not yet been administered in humans. Administration of TB31F in humans may induce an immune response and the production of anti-drug antibodies will be measured throughout the study. Any clinical adverse events occurring as a result of TB31F administration will monitored closely.

b. Previous exposure of human beings with the test product(s) and/or products with a similar biological mechanism

Monoclonal antibodies are approved therapies for malignancies, transplant rejection, autoimmune and infectious diseases. Administration of mAbs carry a risk of immune reactions such as acute anaphylaxis, serum sickness and the generation of anti-idiotypic antibodies to the drug product. In addition, there are numerous adverse effects of mAbs that are related to their specific targets. It is difficult to predict AEs for this investigational product, as this is a first-in human study. However, based on the experience of other mAbs that bind to infectious disease pathogens, it is reasonable to expect both local and systemic AEs may occur.

c. Can the primary or secondary mechanism be induced in animals and/or in ex-vivo

human cell material?

The TB31F mAb was evaluated for toxicity and pharmacokinetics in Sprague Dawley rats. The rat is a standard and accepted species for pharmacokinetic and toxicity studies. The animals were assigned to a dose group using a procedure that stratifies animals across groups by body weight such that mean body weight of each group was not statistically different from any other group using analysis of variance (ANOVA). Forty male and forty female Sprague Dawley rats were allocated to one of six designated dose groups. The animals were administered one of five dose levels of TB31F (18.75, 37.5, 75, 150, 240 mg/kg) or the saline control once via intravenous injection. Serum samples from the PD subset of animals were initially tested in a qualified SMFA with a standardized IgG concentration of 2.5 mg/mL. Inhibition was calculated from the direct count of oocysts in the midgut of mosquitoes. Oocysts were 99.6 to 100% inhibited compared to a negative control ($p = 0.001$). A second experiment was conducted based on set concentrations (1.1, 3.3, and 10 µg/mL) of the recovered TB31F antibody in the spiked human serum, erythrocyte, and gametocyte culture used in the SMFA. Only samples from animals assigned to Groups 2 (18.75 mg/kg) and 6 (240 mg/kg) were evaluated to bracket the dose levels evaluated in vivo. In these assays, we see a concentration dependent inhibition of oocysts, with full (100%) inhibition observed at 10 µg/mL, high levels of inhibition (91.3 to 100%) observed at 3.3 µg/mL, and low to no inhibition (-21.7 to 79.5%) observed at 1.1 µg/mL.

d. Selectivity of the mechanism to target tissue in animals and/or human beings

The TB31F mAb targets the *P. falciparum* Pfs48/45 antigen on the sexual stage of gametocytes which is not expected to be expressed in any normal human or rat tissues. A Tissue Cross-Reactivity (TCR) study of TB31F in normal human and Sprague-Dawley rat tissues was done. Staining with TB31F was observed in the cytoplasm of squamous epithelium in the human esophagus (mucosa), skin (epidermis), thymus (Hassall's corpuscles), tonsil (mucosa, crypts), and uterus (cervix [mucosa]), as well as in the rat esophagus (mucosa). However, cytoplasmic staining in tissue cross-reactivity studies generally is considered of little to no toxicological significance due to the limited ability of antibody therapeutics to access the cytoplasmic compartment in vivo.

e. Analysis of potential effect

None.

f. Pharmacokinetic considerations

The pharmacokinetic evaluation in human subjects will be analyzed from human sera collected at several time points after TB31F administration. See time and event table for details.

g. Study population

Included subjects are healthy young adult subjects, who have been extensively screened for any evidence of co-morbidity, in particular cardiovascular risk factors. Female subjects of child-bearing age are screened for pregnancy by urine test. All subjects (male and female) are required to use

contraception throughout the study period.

h. Interaction with other products

There are no known products which TB31F reacts to other than the Pfs48/45 antigen of *P. falciparum* gametocytes.

i. Predictability of effect

The SMFA is a sensitive predictor of functional activity. When TB31F mAb is incubated in the presence of *P. falciparum* gametocytes and subsequently fed to *Anopheles* mosquitoes, there is > 80% inhibition of the development of oocyst stage parasites in the mosquito with concentrations of TB31F as low as 3.3 µg/ml at concentrations predicted to be readily achieved in human subjects.

j. Can effects be managed?

See section 6.4 for a summary of known and potential risks and benefits that includes mitigation strategies.

13.2 Update of risk assessment for amendment v1.6 (prior to group 5)

Following TB31F administration in groups 1-4, the following update of the risk assessment can be made:

a. Level of knowledge about mechanism of action

TB31F has been administered intravenously to n=20 subjects in groups 1-4. No ADA have yet been measured.

b. Previous exposure of human beings with the test product(s) and/or products with a similar biological mechanism

TB31F has been administered intravenously to n=20 subjects in groups 1-4. Administration is safe and well-tolerated at all administered doses including in the 5 volunteers at the highest dose of 10mg/kg. There have been no grade 3 AEs or SAEs for any of the dosage groups. Two out of 20 volunteers reported local solicited adverse events, both grade 1. The most common general solicited adverse event was grade 1 or 2 fatigue on day of TB31F administration in 12 out of 20 volunteers. Fatigue is a known side-effect of the pre-medication clemastine. TB31F has not previously been administered intradermally, subcutaneously or intramuscularly.

c. Can the primary or secondary mechanism be induced in animals and/or in ex-vivo human cell material?

No change.

d. Selectivity of the mechanism to target tissue in animals and/or human beings

No change.

e. Analysis of potential effect

Preliminary analysis shows that there is a TRA of approximately 50% in group 1 (0.1 mg/kg) at end of infusion. In group 2 (1 mg/kg), TRA of >95% is maintained up to day 28 after TB31F administration.

f. Pharmacokinetic considerations

The pharmacokinetic evaluation in human subjects will be analyzed from human sera collected at several time points after TB31F administration. See time and event table for details. Preliminary analysis of TB31F concentrations in serum over time indicate a half-life of approximately 20.5 days, in line with other human IgG1 molecules.

g. Study population

No change.

h. Interaction with other products

No change.

i. Predictability of effect

Preliminary analysis of the SMFA in group 1 (0.1mg/kg) show a TRA of approximately 50% at end of infusion. With the dosage of group 2 (1mg/kg), a TRA of more than 95% was maintained up to day 28 after administration.

The subcutaneous dosage of 100 mg mAb TB31F is predicted to have >80% inhibition of the development of oocyst stage parasites in the mosquito with >80% 28 days after administration.

The SMFA is a sensitive predictor of functional activity. When TB31F mAb is incubated in the presence of *P. falciparum* gametocytes and subsequently fed to *Anopheles* mosquitoes, there is >80% inhibition of the development of oocyst stage parasites in the mosquito with concentrations of TB31F as low as 3.3 µg/ml at concentrations predicted to be readily achieved in human subjects.

j. Can effects be managed?

See section 6.4 for a summary of known and potential risks and benefits that includes mitigation strategies.

13.3 Synthesis

See section 6.4 for a summary of known and potential risks and benefits that includes mitigation strategies.

14. REFERENCES

1. Organization, G.W.H., *World Malaria Report 2017.* 2017.
2. Organization, G.W.H., *World Malaria Report 2018.* 2018.
3. Mendis, K., et al., *From malaria control to eradication: The WHO perspective.* Trop Med Int Health, 2009. **14**(7): p. 802-9.
4. Bhatt, S., et al., *The effect of malaria control on Plasmodium falciparum in Africa between 2000 and 2015.* Nature, 2015. **526**: p. 207.
5. Griffin, J.T., et al., *Reducing Plasmodium falciparum malaria transmission in Africa: a model-based evaluation of intervention strategies.* PLoS Med, 2010. **7**(8).
6. Sauerwein, R.W. and T. Bousema, *Transmission blocking malaria vaccines: Assays and candidates in clinical development.* Vaccine, 2015. **33**(52): p. 7476-7482.
7. Bousema, T. and C. Drakeley, *Epidemiology and infectivity of Plasmodium falciparum and Plasmodium vivax gametocytes in relation to malaria control and elimination.* Clin Microbiol Rev, 2011. **24**(2): p. 377-410.
8. Roeffen, W., et al., *Plasmodium falciparum: production and characterization of rat monoclonal antibodies specific for the sexual-stage Pfs48/45 antigen.* Exp Parasitol, 2001. **97**(1): p. 45-9.
9. Blagborough, A.M., et al., *Transmission-blocking interventions eliminate malaria from laboratory populations.* Nature communications, 2013. **4**: p. 1812-1812.
10. M. Pinder, V.S.M., K. Mendis, G.V. Brown on behalf of the WHO and M. committee, *MALVAC 2010: Measures of Efficacy of Anti-malarial Interventions against Malaria Transmission.* 2010.
11. UNDP, W.B., WHO Special Programme for Research and Training in Tropical Diseases (TDR), *Malaria transmission blocking vaccines: an ideal public good.* 2000.
12. The MalERA Consultative Group on Vaccines, *A research agenda for malaria eradication: vaccines.* PLoS Med, 2011. **8**(1): p. e1000398.
13. Targett, G.A. and B.M. Greenwood, *Malaria vaccines and their potential role in the elimination of malaria.* Malaria Journal, 2008. **7**(1): p. S10.
14. Riddle, V., et al., *Phase I study evaluating the safety and pharmacokinetics of MDX-1303, a fully human monoclonal antibody against Bacillus anthracis protective antigen, in healthy volunteers.* Clin Vaccine Immunol, 2011. **18**(12): p. 2136-42.
15. Ishida, J.H., et al., *Phase 1 Randomized, Double-Blind, Placebo-Controlled Study of RG7667, an Anticytomegalovirus Combination Monoclonal Antibody Therapy, in Healthy Adults.* Antimicrob Agents Chemother, 2015. **59**(8): p. 4919-29.
16. Doessegger, L. and M.L. Banholzer, *Clinical development methodology for infusion-related reactions with monoclonal antibodies.* Clinical & translational immunology, 2015. **4**(7): p. e39-e39.
17. Ledgerwood, J.E., et al., *Safety, pharmacokinetics and neutralization of the broadly neutralizing HIV-1 human monoclonal antibody VRC01 in healthy adults.* Clin Exp Immunol, 2015. **182**(3): p. 289-301.
18. Lowy, I., et al., *Treatment with monoclonal antibodies against Clostridium difficile toxins.* N Engl J Med, 2010. **362**(3): p. 197-205.
19. Chung, R.T., et al., *Human monoclonal antibody MBL-HCV1 delays HCV viral rebound following liver transplantation: a randomized controlled study.* Am J Transplant, 2013. **13**(4): p. 1047-1054.
20. Pharmacopoeia, E., *Parenteral Preparations.* European Pharmacopoeia 2015. **10.0**: p. 923-925.
21. Fathallah, A.M., et al., *Effects of hypertonic buffer composition on lymph node uptake and bioavailability of rituximab, after subcutaneous administration.* Biopharmaceutics & drug disposition, 2015. **36**(2): p. 115-125.

22. Nony, P., et al., *Impact of osmolality on burning sensations during and immediately after intramuscular injection of 0.5 ml of vaccine suspensions in healthy adults.* Vaccine, 2001. **19**(27): p. 3645-51.
23. Mouton, J.W., et al., *Standardization of pharmacokinetic/pharmacodynamic (PK/PD) terminology for anti-infective drugs: an update.* Journal of Antimicrobial Chemotherapy, 2005. **55**(5): p. 601-607.
24. Mould, D.R. and R.N. Upton, *Basic concepts in population modeling, simulation, and model-based drug development.* CPT: pharmacometrics & systems pharmacology, 2012. **1**(9): p. e6-e6.

15. Appendix 1 – Classification of Adverse Events

The following tables are adapted from the Food and Drug Administration's Toxicity Grading Scale for Healthy Adult and Adolescent Subjects Enrolled in Preventive Vaccine Trials.

Table A1 Grading of local **solicited** adverse events following mAb TB31F Administration

**Local Reaction to mAb TB31F administration**	**Mild (Grade 1)**	**Moderate (Grade 2)**	**Severe (Grade 3)**	**Potentially Life-threatening (Grade 4)**
Pain	Does not interfere with activity	Repeated use of non-narcotic pain reliever > 24 hours or interferes with activity	Any use of narcotic pain reliever or prevents daily activity	Emergency room (ER) visit or hospitalization
Erythema/Swelling	2.5 - 5 cm and does not interfere with activity	5.1 – 10 cm or interferes with activity	> 10 cm or prevents daily activity	Necrosis or exfoliative dermatitis

Table A2 Grading of systemic **solicited** adverse events following mAb TB31F Administration

**Systemic AE**	**Mild (Grade 1)**	**Moderate (Grade 2)**	**Severe (Grade 3)**	**Potentially Life-threatening (Grade 4)**
Fever (measured orally)	38.0 – 38.4 (°C)<br>100.4 – 101.1 (°F)	38.5 – 38.9 (°C)<br>101.2 – 102.0 (°F)	39.0 – 40 (°C)<br>102.1 – 104 (°F)	>40 (°C)
Chills	No interference with activity	Some interference with activity	Significant; prevents daily activity	ER visit or hospitalization
Fatigue	No interference with activity	Some interference with activity	Significant; prevents daily activity	ER visit or hospitalization
Headache	No interference with activity	Repeated use of non-narcotic	Significant; any use of narcotic pain reliever or [illegible]	ER visit or hospitalization
Myalgia	No interference with activity	Repeated use of non-narcotic	Significant; prevents daily activity	ER visit or hospitalization

Vital Signs

Table A3. Vital signs

Vital Signs	Mild (Grade 1)	Moderate (Grade 2)	Severe (Grade 3)	Potentially Life Threatening (Grade 4)
Fever (°C)	38.0 – 38.4	38.5 – 38.9	39.0 – 40	> 40
Tachycardia (beats/minute)	101 – 115	116 – 130	> 130	ER visit or hospitalization for arrhythmia
Bradycardia (beats/minute)	50 – 54	45 – 49	< 45	ER visit or hospitalization for arrhythmia
Hypertension (systolic) (mmHg)	141 – 150	151 – 155	> 155	ER visit or hospitalization for malignant hypertension
Hypertension (diastolic) (mmHg)	91 – 95	96 – 100	> 100	ER visit or hospitalization for malignant hypertension
Hypotension (systolic) (mmHg)	85 – 89	80 – 84	< 80	ER visit or hospitalization for hypotensive shock
Respiratory Rate (breaths/minute)	17 – 20	21 – 25	> 25	Intubation

Laboratory abnormalities

Table A4. Laboratory abnormalities

**Adverse Event**	**Intensity Grade**	**VALUE TB31F**
Hemoglobin , male (mmol/L)	1	7.8-8.3
	2	6.5-7.7
	3	5.2-6.4
Hemoglobin, female (mmol/L)	1	6.8-7.3
	2	5.9-6.7
	3	5.0-5.8
Increase in Leukocytes (WBC) (cells/mm^3)	1	11,000 – 15,000
	2	15,001 - 20,000
	3	20,001 - 25,000
Decrease in Leukocytes (cells/mm^3)	1	2,500 - 3,500
	2	1,500 - 2499
	3	1,000 -1,499
Decrease in Platelets (cells/mm^3)	1	125,000 – 140.000
	2	100,000 - 124,000
	3	25,000 – 99,000
Creatinine, mg/dL	1	1.5-1.7
	2	1.8-2.0
	3	2.1-2.5
Transaminases (AST) (U/L)	1	1.1-2.5 x ULN
	2	2.6-5 x ULN
	3	5.1-10 x ULN
Transaminases (ALT)	1	1.1- 2.5 x ULN

**Adverse Event**	**Intensity Grade**	**VALUE TB31F**
(U/L)	2	2.6 - 5 x ULN
	3	5.1-10 x ULN

Appendix 2

Group 1 – 0.1 mg/kg

	mg/hour
start	50

Total: +/- 30 min

Group 2 – 1 mg/kg

	mg/hour
start	50
30 min	100
60 min	100

Total: +/- 1h

Group 3 – 3 mg/kg

	mg/hour
start	100
30 min	200
60 min	300
90 min	300

Total: <90min

Group 4 – 10 mg/kg

	mg/hour
start	200
30 min	300
60 min	400
90 min	500
120 min	500

Total: +/- 120min
